# Conformational and Tautomeric Control by Supramolecular Approach in Ureido-*N*-*iso*-propyl,*N*’-4-(3-pyridin-2-one)*pyrimidine*

**DOI:** 10.3390/molecules24132491

**Published:** 2019-07-08

**Authors:** Adam Kwiatkowski, Erkki Kolehmainen, Borys Ośmiałowski

**Affiliations:** 1 Faculty of Chemistry, Nicolaus Copernicus University in Toruń, 7 Gagarin Street, 87-100 Toruń, Poland; 2 Department of Chemistry, University of Jyväskylä, P.O. Box 35, FI-40014 Jyväskylä, Finland

**Keywords:** tautomerism, intermolecular interactions, hydrogen bonding, molecular switch

## Abstract

Ureido-*N*-iso-propyl,*N*’-4-(3-pyridin-2-one)pyrimidine (**1**) and its 2-methoxy pyridine derivative (**1Me**) has been designed and prepared. The conformational equilibrium in urea moiety and tautomerism in the pyrimidine part have been investigated by variable temperature and ^1^H NMR titrations as well as DFT quantum chemical calculations. The studied compounds readily associate by triple hydrogen bonding with 2-aminonaphthyridine (**A**) and/or 2,6-bis(acetylamino)pyridine (**B**). In **1**, the proton is forced to 1,3-tautomeric shift upon stimuli and keeps it position, even when one of the partners in the complex was replaced by another molecule. The observed tautomerism controlled by conformational state (kinetic trapping effect) opens new possibilities in molecular sensing that are based on the fact that reverse reaction is not preferred.

## 1. Introduction

It is known that proton transfer reactions are quite hard to control. Those reactions drive the tautomeric equilibria, proton-transfer in conducting materials, acidity, and many others. Thus, the way to control the position of proton in the specific place in molecule is important in the light of intermolecular interactions, as, for example, in ureidopyrimidones [1]. Thus, the rotamerism in heterocyclic urea derivatives [2] and proton transfer may be used in the design of molecules. On the other hand, such property is even more interesting if one is able to use it for pushing proton towards specific location. However, this is not easy, owing to protons mobility and molecular flexibility. 

Our interest is focused on molecules comprising several flexible moieties that are capable of forming several tautomers and rotamers, although they are very challenging targets of investigation. Various nitrogen heterocycles are worth to be studied in spite of this difficulty, owing to their biochemical importance. We selected it instead of more complex purine moiety containing four nitrogen atoms while taking into account the fact that pyrimidine moiety is a structural component in biopolymers, such as DNA and RNA. In addition, the substituent effects on the tautomerism of pyridine derivatives is well known [3]. We designed and prepared ureido-*N*-iso-propyl,*N*’-4-(3-pyridin-2-one)pyrimidine (**1**) and its 2-methoxy pyridine derivative (**1Me**) where the aromatic pyridine moiety is present as, so called, “fixed tautomer” based on our earlier experiences on syntheses of urea and pyridine derivatives [4].

In our opinion, the structures of **1** and **1Me** can be considered to be molecular machines that perform some action upon their exposition to external stimuli, such as solvent, temperature, and guest molecules. They can also act as molecular switches, which can change their structures upon interaction by locking respective conformation with intramolecular hydrogen bonding,, as it was shown for other molecules, both in solution [5,6] or at the surface [7]. In this context, the change must take place between two thermodynamically stable states. The switching mechanism usually relies on (*a*) *trans*/*cis* photoisomerization [8], (*b*) conformational change [9], (*c*) bond formation [10], (*d*) photo-switching receptor molecules [11], and other. The switching between various states requires the stimulus—the delivery of energy or interactions with another molecules [12]. The orbital energy levels of the host are changing if the latter is true, which can be detected by spectral methods [13]. One of the most common topics is monitoring the cation/anion binding. Among them are molecules that fit the guest by changing geometry of the sensor on its binding site [14,15,16]. However, so far, the reports on the switches exhibiting tautomerism are relatively rare [17,18]. One reason can be in difficulties to study fast proton transfer reactions [19]. The tautomerism itself can be influenced by a variety of factors, such as benzannulation (qualitative influence) [3,20] and by the attached substituent (quantitative influence) that forces the percentage of one form from insignificant to nearly 100% [3]. The intramolecular hydrogen bonding (also the resonance assisted hydrogen bond–RAHB [21]) is responsible for the stabilization of number of structures. It is known that RAHB is an important factor that influences stability of respective forms, although some discussion persists [22]. Nevertheless, the intramolecular hydrogen bonding (IMHB), resonance assisted or not, is a factor that can be utilized in design of molecular switches.

From the supramolecular chemistry point of view, the urea that was capable to interact via two hydrogen bonds is a very useful moiety for sensing [23], catalysis [24], or in self-organization of molecules in crystal [25,26], and also in non-covalent polymerization [27,28]. It is also possible to tune the properties of urea by proper substitution or by supramolecular approach [4], id est. by interaction with designed hosts. Thus, the intramolecular hydrogen bond (IMHB) in heterocyclic urea derivatives can be broken by interaction with anions [29] or neutral molecules [4]. The break two intramolecular hydrogen bonds is also possible [30]. while some of those processes are still under discussion [31]. However, the NMR [32,33,34,35] and DFT [36,37,38] methods are one of the most often used in studies on tautomerism in solution especially when experimental data are difficult to interpret.

**Figure 1:**
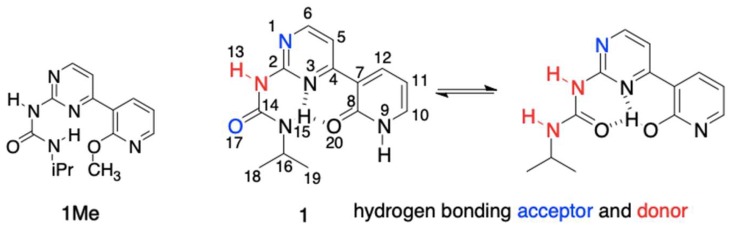
The structures of **1** and **1Me** and their atomic numbering.

In the current study, we have designed and synthesized compounds **1** and **1Me** (Figure 1) joining the topology of urea capable to form intramolecular hydrogen bond(s) with biochemically important pyridine and pyrimidine moieties.

The pyridine part possesses well-known tautomeric preferences being in **1Me** completely as imino tautomer, while pyrimidine allows for the formation of two different IMHBs.

## 2. Results and Discussion

**Figure 2:**
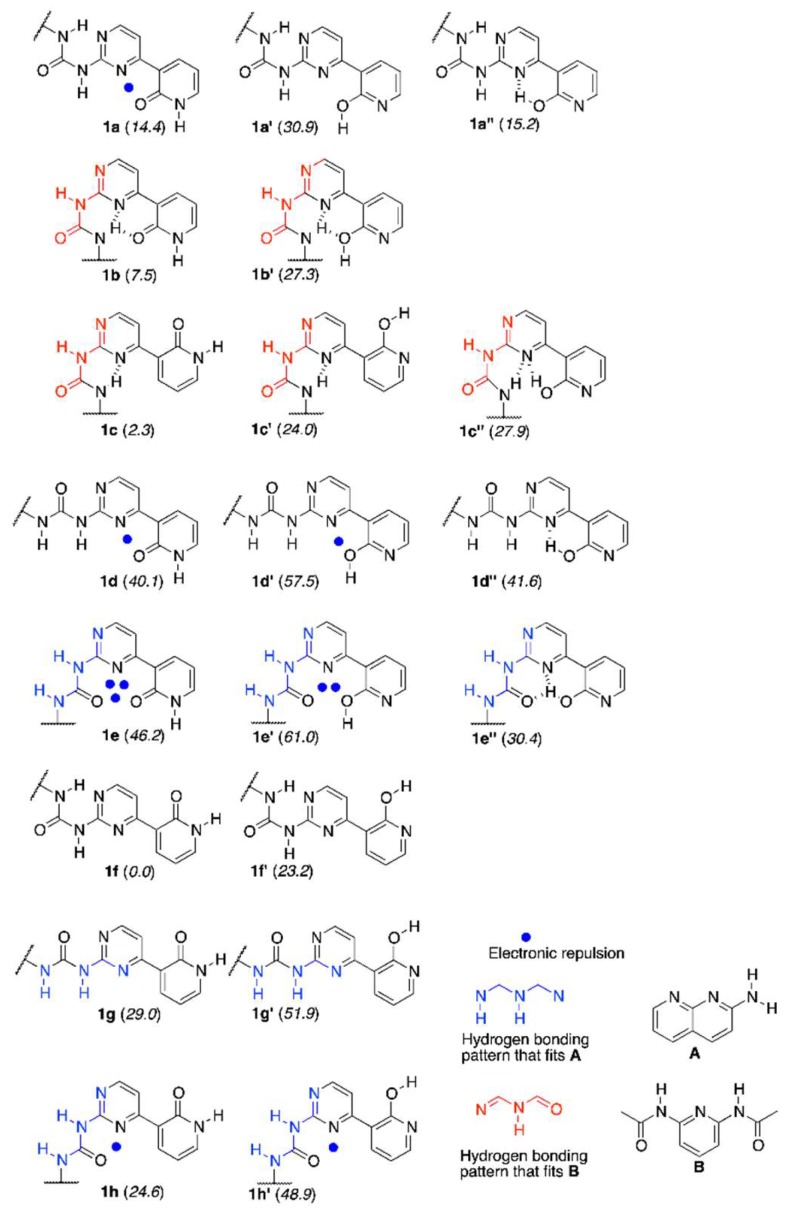
The tautomeric forms of the rotamers of **1**, their DFT-based relative energies (kJ/mol) and structures of guest molecules **A** and **B**.

The synthesis of **1** and its methoxy derivative, **1Me**, which is also called the “fixed-tautomer” [39] is described in Section 3. Figure 1 shows the structure and atom numbering of **1**/**1Me**, while in Figure 2 the multiple equilibrium in **1** is shown with the relative energies [kJ/mol] obtained by DFT calculations (in parentheses close to each form). Another set of data from DFT were also placed in the Supplementary Materials. The labels from **a** to **h** represent various series of rotamers (still tautomerism is possible within each series), with the emphasis on the possible intermolecular interaction with **A** (in blue) and **B** (in red).

It is known that pyrid-2-one is more stable tautomeric form than 2-hydroxypyridine, but the equilibrium in solvent-dependent [40] (polar solvents favor keto form). In the current study, chloroform was selected (logK_T_ = 0.78 for NH/OH forms in that solvent [40]), owing to its polarity and use as a model solvent for other hydrogen bonded complexes [41]. It is also worth reminding that the intramolecular hydrogen bond is more probable than intermolecular one [42]. Taking this into account, it is reasonable to mention that the urea part is able to form IMHB with N1 or N3 in pyrimidine, while the labile proton is located at N9 of pyridine part. That makes the existence of two forms (**1a** and **1b** and their rotamers **1c** and **1f**) probable.

### 2.1. NMR Measurements

The ^1^H NMR dilution and titration experiments were conducted to investigate the possible dimerization of **1** and its association with suitable counterparts (2-aminonaphthyridine (**A**) and 2,6-bis(acetylamino)pyridine (**B**)). The dimerization (K_dim_) and association (K_assoc_) constants have been calculated by the chemical shift changes of chosen protons. The K_dim_ was found to be 27 M^−1^ for **1** and 22 M^−1^ for **1Me** (the dilution data are almost linear with concentration, so the interaction is weak), which is lower than similar compounds [4] due to the higher flexibility of **1** and **1Me** than the previously studied molecules. The titration of **1** with **A** and **B** and **1Me** with **B** gave association constants (K_assoc_) values that are collected in Table 1.

**Figure 3:**
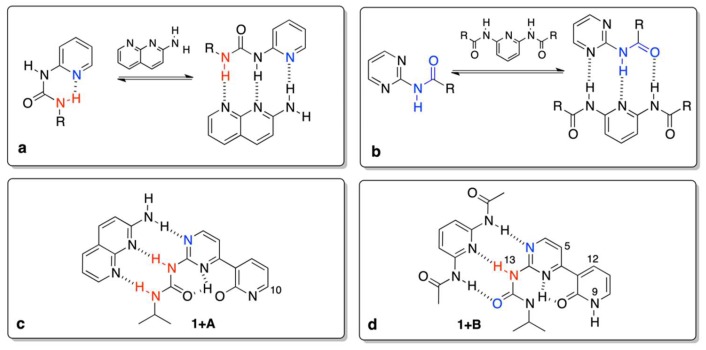
The previously (**a**,**b**) and currently (**c**,**d**) studied complexes.

Now, it is reasonable to compare the values in Table 1 with those that were reported earlier. The association of a simple *N*-pyridin-2-yl-*N*’-ethyl-urea (Figure 3a) with **A** gave the K_assoc_ = 11 M^−1^ [4], while for similar triply hydrogen–bonded complexes (Figure 3b) in chloroform the association constant is ca. 30 M^−1^ [44]. Currently, we have observed order of magnitude larger values for association, which can be explained by the higher rigidity of respective rotameric forms in the complexes caused by intramolecular hydrogen bonding (for example in **1e’’**) of the pyridine moiety (Figure 3c,d). The stabilization by intramolecular hydrogen bonding is seen in the change of chemical shift of H9 (labile proton that changes it position form H9 to H20 due to tautomerism) and H10 (the closest proton to the tautomerization site, see Figure 1 for atom numbering). The said changes are equal to ca. 4 and 0.75 ppm for H9/H20 and H10, respectively. It is important to note that the resulting δ(H10), equal to 8.3 ppm (Figure 3c), is very close to the value that was observed for 2-methoxypyridine, so both of the protons have a similar chemical environment (OH and OMe groups) [45]. The relatively high value of the chemical shift for H9/H20 at the final step of titration (δ = 14.9 ppm) is explained by (a) its bonding to oxygen atom and (b) formation of strong intramolecular hydrogen bonds, as in similar pyridine derivatives [3]. That keeps the form **1e’’** rigid. On the other hand, **1** also readily associates with **B**, but differences are evident, *id est.* (a) the chemical shift of H9 is constant (ca. 9 ppm) during the course of titration, (b) as opposite to **1 + A** complex, shift for H13 proton increases in **1 + B**, (c) protons H5 and H12 changes slightly, which is most probably due to the rotation of the pyridone moiety and the effect of anisotropy of C=O and N on those shifts (rotational equilibrium between **1f**, **1c**, and **1b** supported by DFT calculations).

To have a further insight into the hydrogen bonding in **1,** its “fixed-tautomer” **1Me** was also studied. The results show that **1Me** does not associate with **A**, but it forms complex with **B**. That means the H15···N3 and H15···O20Me bifurcation in hydrogen bonding (Figure 1, left hand-side structure with O20-Me moiety) plays a key role in stabilization. Beside the association itself, one of the criteria showing the formation of hydrogen bond is the complexation-induced shift (CIS). This value is a difference between δ(ppm) for a nucleus in the free molecule and for the same nucleus when the concentration of guest is extrapolated to infinity. It is worth mentioning that the CIS values (H5, H13 and H15) in **1Me + B** (−0.21, 4.25 and 0.40) are slightly higher than that in **1 + B** complex (−0.17, 2.20 and 0.18). That suggests the OMe group attached to pyridyl (a) rigidifies molecule by hydrogen bonding with H15 and (b) prove the tautomerism in **1** is the *condicio sine qua non* of its binding with both counterparts. It is also worth mentioning that, for **1** and **1Me**, the structural change (tautomerism) is crucial (keto vs. *fixed* enol form). During an additional experiment **1** was titrated with the use of tetrabutylammonium benzoate as before [29]. An existence of a multiple equilibrium is manifested[46] by the deviation of the titration curve from Benesi–Hildebrand equation for H10 and H11 with the largest Δδ = 0.40 (CIS) ppm being observed during titration for aromatic CH (H10, doublet) excluding the data form calculation of the association constant. We concluded that deprotonation takes place, since the H9 proton is not visible when benzoate salt was added and the CIS for H10 is the largest. This shows the conformational freedom and proton mobility in **1** and it confirms a fast exchange in the NMR time-scale.

### 2.2. ^1^H VT (Variable Temperature) NMR

**Figure 4:**
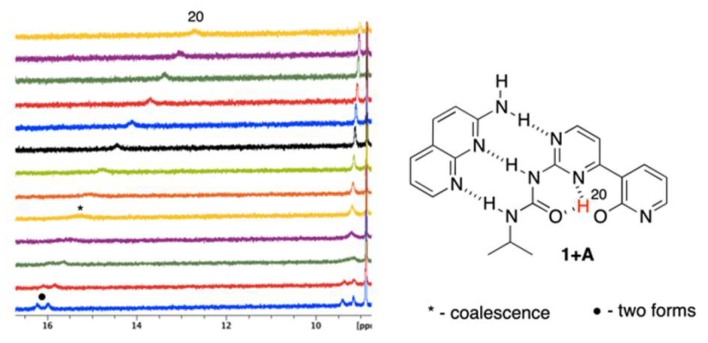
The VT spectra for **1 + A** complex (+20 to −40 °C, from top to bottom).

**Figure 5:**
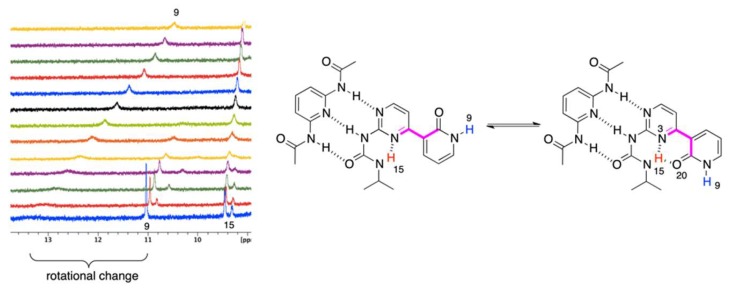
The VT spectra for **1 + B** complex (+20 to −40 °C, from top to bottom).

The temperature influences chemical shifts weakening non-covalent interactions at higher temperatures in structures that are stabilized by intramolecular hydrogen bonding. In pure **1,** the temperature effect on the NMR data was insignificant (also excluding its possible strong dimerization), but it was visible for the complexes of **1**. The mixtures (*ca.* 1:1) of **1 + A** and **1 + B** were measured in the range of +20 °C to −40 °C by 5 °C steps. Figure 4 and Figure 5 show the partial ^1^H NMR spectra at the deshielded regime.

It is easy to notice (Figure 4) that the H20 proton shifts from ca. 13 to more than 16 ppm with simultaneously splitting into two signals (as with other protons). Those may come from (*a*) ^1^J_NH_ coupling or (*b*) multiple equilibrium within IMHB interactions (NH···O and NH···N ones). As similar splitting was observed for other protons (at ca. 9.4 ppm) in **1 + A,** we concluded that it was due to an equilibrium. Thus, lowering the temperature limits the rotation around single bonds, making molecule more rigid, since the geometry of **1** is most probably fixed by bifurcated intramolecular hydrogen bonding. This is concluded based on relatively small differences between chemical shits of the respective protons that spitted into two signals (Figure 4, H20). Similar behavior was observed in case of **1 + B** complex (Figure 5) for the signal at ca. 9.2–9.5 ppm. At the same time, one signal is getting sharper (*ca.* 11 ppm at −40 °C) and another is getting broader (*ca.* 13.2 ppm at −40 °C). All of the signals are deshielded upon cooling the sample. At low temperatures rotation around single bonds is slowed down and the hydrogen bonding freezes molecular geometry, which makes those forces stronger. Thus, as in the case **1 + A** complex, the rotation of the 2-pyridone moiety causes appearance of additional signals. The signal of H15 splits due to the coexistence of two rotameric forms (N3-C4-C7-C8 dihedral equal to 0 or 180°, Figure 5 in pink). Again, the difference in chemical shifts at ca. 9.5 ppm is small. The change of chemical shift of H9 is more complex. At first, its signal is visible at ca. 10.6 ppm, while, the sample it becomes deshielded upon cooling, broadens, and a new signal appears at ca. 11 ppm being sharp at −40 °C. Two effects may explain this. First, the rotational equilibrium (suggested by observation of H15) and, secondly, different sensitivity of H9 chemical shift to the temperature in two rotameric forms. In the case of the structure stabilized by bifurcated hydrogen bonding between H15 and N3/O20 (Figure 5, right-hand side complex), the chemical shift of H9 is less sensitive to temperature and interaction with solvent due to the steric reasons. In another rotameric form that is characterized with a N3-C4-C7-C8 dihedral angle equal to 180°, the potential accessibility of H9 to solute-solvent interaction is much higher (no *i*Pr group in close proximity), thus its chemical shift may more evidently change.

To sum up, decreasing the temperature causes several effects. The most important is that the rotational and geometrical movements of molecular moieties are limited at lowered temperature. In the half-way between the coalescence causes signals broadening. In fact, this is evidently only seen for **1 + A** complex with gentle split of signals at low temperature, while for **1 + B,** two much separated signals are observed to be caused by extreme dihedral angle change.

### 2.3. Guest Replacement

The final experiment was the substitution of **A** by **B** in the complex **1 + A**. At the first step, the ^1^H NMR titration of **1** by **A** was conducted, and the resulting complex was then titrated in the same NMR-tube with **B**. For the urea NH protons in **1,** the following was observed (see Figure S8 in Supplementary Materials). Upon **1 + A** titration chemical shift of H15 increased and the same was observed (further increase) during the addition of **B** to the solution. However, the curve became a little steeper in **1 + A + B** part than that in **1 + A** part. The above-described changes are much more dramatic in the case of H13. When **B** was stepwise added to the solution of **1 + A** the chemical shift of H13 increased by 1.7 ppm. Unfortunately, at some point, the H13 signal overlapped by NH_2_ signal of **B**. Anyway, the substitution of **A** by **B** in the complex was confirmed. The most surprising was that the chemical shift of H9 increased when **1** was titrated by **A** and did not change its position when **B** was added to **1+A** mixture. That suggests that the OH⋅⋅⋅N and NH⋅⋅⋅N hydrogen bonds keep the geometry in **1c’’** conformation. Thus, the guest replacement is only realized with the change of the rotational state, but not by the change of the tautomeric one. This is interpreted as the kinetic trapping effect [47], which allows for producing kinetic product instead of the thermodynamic one. The prolonged time after the experiment and temperature increase did not influence the equilibrium, meaning that the energy barrier of the proton transfer is high enough to allow the rotation of the urea moiety.

Unfortunately, all of the attempts to get proper crystals of **1** and its complexes for X-ray diffraction structural analysis failed.

### 2.4. DFT Calculations

A series of DFT calculations were carried out to have a further insight into the complexes of **1**. Additionally, the QTAIM-based [48] Espinosa’s [49,50] approach was used in calculation of the energy of hydrogen bonds to have a full picture of the hydrogen bonding.

The calculation of relative energy gave the set of data shown in Figure 2. The intermolecular energy calculations for dimers of **1b**, **1c,** and **1f** showed that the most probable dimeric structures are those that are built from **1f** and **1c** forms. Two **1f_2_** dimers (see Supplementary Materials) and one **1c_2_** dimer lie within ca. 6 kJ/mol relative energy (E_rel_). The intermolecular interactions (E_int_) have relatively higher values (ca. −45 kJ/mol) than that for remaining dimers (ca. −22 to −36 kJ/mol) for the same dimers. Regarding the complexes of **1** the most stable one with **A** is **1h + A**, while **1e’’ + A** is only 3 kJ/mol higher in energy. On the other hand, the E_int_ for **1e’’ + A** is slightly higher than that for **1h + A**. The highest E_int_ within **1 + A** complexes was shown by **1e’’ + A(PT)** (N3 protonated, see Supplementary Materials), but this tautomeric form of the complex has E_rel_ equal to 27.9 kJ/mol, so its existence is not probable. In the complexes with **B,** the most stable is **1c + B** and **1b + B** is 3 kJ/mol higher in energy. This shows that multiple-equilibrium is possible in **1** and its complexes.

The hydrogen bonding in **1** and complexes is, in general, in agreement with Etter’s rules [42] (more polar groups form stronger interactions). The strongest HB (OH···N3) was found in **1a’’**, **1d’’**, and **1e’’** (−55.9, −57.2, and −51.3 kJ/mol, respectively). As opposite to **1e’’ + A** complex (vide infra), no hydrogen bond between H20 and O17 was found for isolated **1e’’**. The intermolecular hydrogen bonding (E_HB_) in the dimer and complexes have values that range from −36.8 to −4.6 kJ/mol for weak CH···O contact for dimers and from −27.6 to −12.2 kJ/mol for complexes. For the intramolecular HB’ing in complexes E_HB_ varies between −50.7 and −10.1 kJ/mol. It might be concluded that such a large variability is caused by multiple forms that are present in the studied molecule while taking into account the given ranges of E_HB_. 

The path leading from the most stable **1f** form to the one that is able to interact by triple hydrogen bonding with **A** was considered in the order of forms, as follows: **1f** → **1c** → **1b** → **1b’** → **1e’** → **1e’’** and rationalized in the light of DFT computations, including transition states. The most important is that the highest energy barrier to overcome in the mentioned path has a value of 143.2 kJ/mol and it is related to the proton shift. That kind of barrier should be taken into account for the explanation of previous results in tautomerizable switches [51]. More detailed discussion is collected in the Supplementary Materials.

## 3. Materials and Methods

The structure of synthesized compounds was confirmed and the chemical shifts were assigned by the analysis of ^1^H, ^13^C, ^1^H, ^1^H COSY, ^1^H,^13^C HSQC, ^1^H, and ^13^C HMBC spectra. All of the spectra were recorded at 400MHz spectrometer at room temperature in CDCl_3_ or DMSO-*d*_6_ for structure conformation and in CDCl_3_ for variable temperatures experiments. The NMR titration (in CDCl_3_, r.t.) data were fitted with the use of Bindfit (Nelder–Mead algorithm) while using the 1:1 stoichiometry model. The following concentrations for the host molecule (**1** or **1Me**) were used: 0.00317 mol/dm^3^ (titration of **1**), 0.00325 mol/dm^3^ (titration of **1Me**). The initial concentration of **1** and **1Me** during dilution studies was 0.00317 mol/dm^3^ and 0.01100 mol/dm^3^, respectively. In all titrations, the solvent used was dried with the molecular sieves before use.

### 3.1. Synthesis

**Figure 6:**
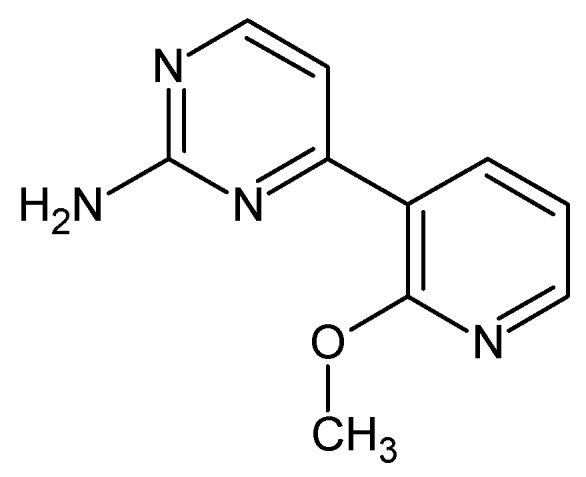
The structure of 4-(2-methoxypyridin-3-yl)pyrimidin-2-amine.

*4-(2-Methoxypyridin-3-yl)pyrimidin-2-amine (Figure 6).* A 100 mL round bottom flask was sequentially charged with Pd(OAc)_2_ (0.12 g, 2 %mol), PtBu_3_·HBF_4_ (0.18 g, 2.4 %mol), 2-Amino-4-chloropyrimidine (4 g), 2-methoxy-3-pyridinylboronic acid (5.65 g, 1.2 eq.), and 40 mL of *n*-butanol. Mixture was stirred at 80 °C under nitrogen for 15 min and then NaOH (2.09 g, 1.7 eq.) was added to initiate the reaction. After 30 min, the heating was stopped and the reaction was stirred overnight. After 15 h, the reaction mixture was poured over 300 mL Et_2_O, the precipitate separated, and the organic phase was concentrated on rotary evaporator. Afterwards, resulting residue was recrystalized twice from MeOH giving 4.07 g of white-yellow crystals (61% yield). M.p. 151–154 °C (dec.). ^1^H NMR (TMS, DMSO, 400.13 MHz, 295 K) δ: 8.30–8.28 (m, 3H), 7.18–7.14 (m, 2H), 6,67 (bs, 2H), and 3.96 (s, 3H). ^13^C NMR (TMS, DMSO, 100.61 MHz, 295 K) δ: 164.14, 161.43, 161,27, 158.97, 148.61, 139.43, 120.96, 117.81, 110.38, and 53.96. Elemental for C, 59.40; H, 4.98; N, 27.71; found C, 59.46; H, 5.08; N, 27.65.

**Figure 7:**
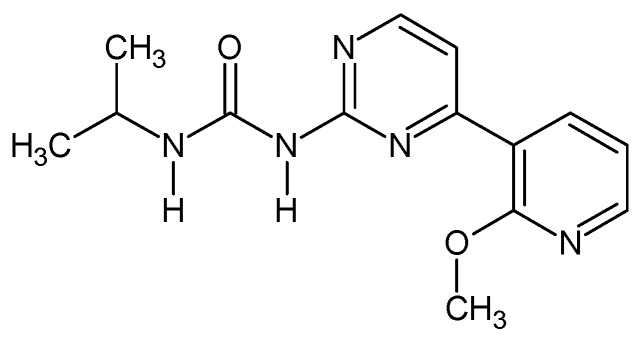
The structure of 1-[4-(2-methoxypyridin-3-yl)pyrimidin-2-yl]-3-propan-2-yl urea.

*1-[4-(2-Methoxypyridin-3-yl)pyrimidin-2-yl]-3-propan-2-yl urea (Figure 7).* In 10 mL round bottom flask 0.35 g of 4-(2-methoxypyridin-3-yl)pyrimidin-2-amine was dissolved in 5 mL of THF and 0.083 g NaH was added (2 eq). The mixture was stirred at r.t. for 15 min and then 0.147 g of isopropyl isocyanate was added and the resulting mixture was heated for three days at b.p. After that the reaction was quenched with ca. 1 mL or water, solvent evaporated while the residue was recrystalized from methanol giving 0.293 g (59%) of white powder. M.p. 165–167 °C. ^1^H NMR (TMS, CDCl_3_, 400.13 MHz, 295 K) δ: 8.99 (d, ^3^*J*_HH_ = 8.0 Hz, 1H), 8.54 (d, ^3^*J*_HH_ = 5.6 Hz, 1H), 8.26–8.32 (m, 2H), 7.75 (bs, 1H), 7.64 (d, ^3^*J*_HH_ = 5.2 Hz, 1H), 7.06–7.08 (dd, ^3^*J*_HH_ = 7.6 Hz, 1H), 4.11 (m, 1H), 4.07 (s, 3H), 1.26–1.28 (d, ^3^*J*_HH_ = 6.4 Hz, 6H). ^13^C NMR (TMS, CDCl_3_, 100.61 MHz, 295 K) δ: 161.71, 158.07, 157.87, 153.50, 149.22, 139.03, 119.78, 117.30, 114.43, 53.80, 42.05, 23.16. Elemental for C, 58.52; H, 5.96; N, 24.37; found C, 58.45; H, 6.06; N, 24.20.

**Figure 8:**
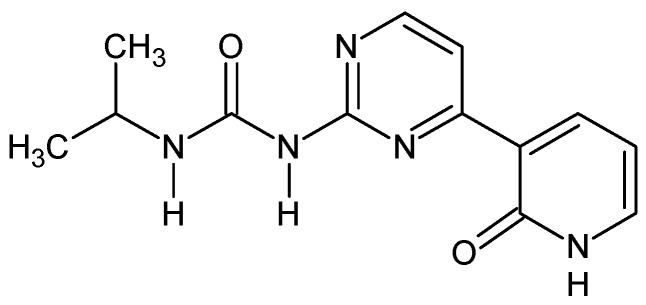
The structure of ureido-*N*-iso-propyl,*N*’-4-(3-pyridin-2-on)pyrimidine.

*Ureido-N-iso-propyl,N’-4-(3-pyridin-2-on)pyrimidine (Figure 8) [52].* To the solution of 0.25 g of 1-[4-(2-methoxypyridin-3-yl)pyrimidin-2-yl]-3-propan-2-yl urea in anhydrous DCM (5 mL) at −20 °C, was slowly added a solution of 1.2 eq. of boron tribromide (0.26 g, 3 mL in anhydrous DCM). After one hour of stirring at r.t., the reaction mixture was poured onto 1 M HCl (10 mL). The solution was stirred for 15 min. and evaporated. Residue was recrystalized from methanol then purified by column chromatography (silca gel, acetone and then MeOH) giving 0.11 g (45%) of white yellow powder. M.p. 240 °C (dec.). ^1^H NMR (TMS, DMSO, 400.13 MHz, 295 K) δ: 12.17 (bs, 1H), 9.56 (s, 1H),9.05 (d, ^3^*J*_HH_ = 7.2 Hz, 1H), 8.57 (d, ^3^*J*_HH_ = 5.3 Hz, 1H), 8.40–8,42 (dd, ^3^*J*_HH_ = 7.2 Hz, 1H), 8.11 (d, ^3^*J*_HH_ = 5.3 Hz, 1H),7.68–7.69 (dd, ^3^*J*_HH_ = 6.2 Hz, 1H), 6.47 (t, 1H), 3.85–3.93 (m, 1H), 1.20 (d, ^3^*J*_HH_ = 6.6 Hz, 6H) ^13^C NMR (TMS, DMSO, 100.61 MHz, 295 K) δ: 161.41, 161.00, 158.97, 158.35, 153.69, 142.09, 139.82, 124.40, 112.84, 105.84, 41.64, 23.35. Elemental for C, 57.13; H, 5.53; N, 25.63; found C, 57.25; H, 5.62; N, 25.47.

### 3.2. Calculations

The structures of all the rotamers shown in Figure 2 and their complexes with **A** and **B** were optimized at the M05/6-311+G(2d,2p) level in chloroform as a solvent (PCM model of solvation [53]), only yielding positive frequencies confirming stable geometry. Moreover, path transition states were also optimized with the use of the STQN [54] method to study the conformational change, as implemented in Gaussian [55]. That gave one imaginary frequency for every transition state. All of the intermolecular interactions were corrected to basis set superposition error by counterpoise method and zero-point energy.

## 4. Conclusions

Ureido-*N*-*iso*-propyl,*N*’-4-(3-pyridn-2-one)pyrimidine **1** and its 2-methoxy pyridine derivative **1Me** are designed and synthetized as the model compounds for an investigation of simultaneous multiple equilibria. The DFT calculations are crucial and supporting NMR data to figure out the most significant features, owing to the complex equilibria the results that were obtained by ^1^H NMR titration, as well as variable temperature and complex formation effects on ^1^H NMR. It was shown that in **1** the tautomeric equilibrium can be controlled by conformational states, making its use possible in many applications (molecular switch, etc.). The structural complexes of **1** with 2-aminonaphthyridine (**A**) and 2,6-bis(acetylamino)pyridine (**B**) are stabilized by multiple intermolecular interactions. The tautomeric state stabilized by the interaction with one guest molecule was preserved, even when another competing guest was added. That is possible due to the relatively high energy barrier of the prototropic reaction.

## Figures and Tables

**Table 1:** The association constants ^a^ (M^−1^) for **1 + A**, **1 + B** and **1Me + B** complexes.

Comp./Probe ^b^	H5	H6	H9	H13	H15
**1 + A**		608 (7 ^c^)	448 (4)	767 (10)	699 (11)
**1 + B**	1656 (15)			1253 (10)	932(14)
**1Me + B**	162 (4)			110 (4)	125 (4)

^a^—all constants were calculated using Bindfit [43], ^b^—in the first row the protons in **1**/**1Me** that were used as probes in titration experiments are given, ^c^—error of estimation.

## Supplementary Materials

The following are available online. Cartesian coordinates for optimized structures, additional discussion, Table S1. The relative energy of monomeric forms of **1**, Figure S1. Investigated dimers of **1**, Table S2. The relative and interaction energy obtained for dimers of **1**, Figure S2. The structure of **1h + A** complex, Figure S3. The energy diagram for **1f**→**1e’** transition, Table S3. The relative and interaction energy obtained for complexes of **1** and **A**/**B**, Figure S4. **1 + A** ^1^H NMR titration, Figure S5. **1 + B** 1H NMR titration, Figure S6. The dilution experiments for **1** (top) and **1Me** (bottom), Figure S7. The stacked spectra for titration of **1 + A** by **B**, Figure S8. ^1^H NMR titration of **1 + A** with **B**, Figure S9. **1Me + B** ^1^H NMR titration, NMR spectra.
