# Supplementary material for: Conformational and Tautomeric Control by Supramolecular Approach in Ureido-N-iso-propyl,N’-4-(3-pyridin-2-one)pyrimidine
Source: Molecules. 2019 Jul 8;24(13):2491. doi: 10.3390/molecules24132491 (PMC6651695; doi:10.3390/molecules24132491)
Supplement: Supplementary file 1 [file molecules-24-02491-s001.pdf]

Supplementary Information

Conformational and tautomeric control by supramolecular approach in ureido-N-iso-propyl,N'-4-(3-pyridin-2-one)pyrimidine

Adam Kwiatkowski, Erkki Kolehmainen and Borys Ośmiałowski*

Faculty of Chemistry, Nicolaus Copernicus University in Toruń, 7 Gagarin Street, 87-100 Toruń, Poland
and
Department of Chemistry, P.O. Box 35, FI-40014 University of Jyväskylä, Finland

1 CARTESIANS FOR OPTIMIZED STRUCTURES 2
2 ADDITIONAL DISCUSSION 50
2.1 THE STABILITY OF TAUTOMERIC/ROTAMERIC FORMS 50
2.2 STABILITY OF DIMERS 52
2.3 COMPLEXES AND THEIR PROPERTIES 54
3 FIGURES FOR TITRATION OF 1 57
4 NMR SPECTRA 64

1 Cartesians for optimized structures

1a

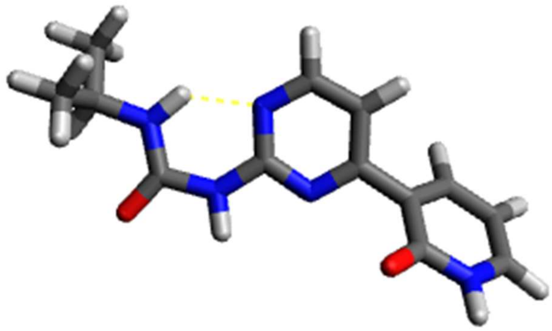

E(a.u.)= -928.0442433

N	-1.5659893999	2.3678131516	0.0515371105
C	-1.6030829888	1.0383151252	-0.0478934062
C	-0.3509057165	2.9018559565	0.1675087396
C	0.8046064340	2.1490912799	0.1863970906
C	0.6513759700	0.7642747071	0.0768650353
N	-0.5542032841	0.2107615082	-0.0394323013
C	1.8293485973	-0.1271057474	0.0502544840
C	1.8736294476	-1.2945778005	0.9127585762
N	3.0246264139	-2.0644784451	0.7471692278
C	4.0506173984	-1.7938892489	-0.0897718173
C	4.0183192622	-0.6811846685	-0.8702100943
C	2.8855622701	0.1525550022	-0.7785854165
N	-2.8194921384	0.4023269533	-0.1755569024
C	-4.1410391458	0.8667541282	-0.2593911764
N	-4.3251319543	2.1951594740	-0.2159603245
C	-5.6450244498	2.8140482120	-0.2532359906
O	-5.0349313640	0.0361153346	-0.3767228923
H	-0.3095100371	3.9819067373	0.2549715310
H	1.7734447823	2.6103573808	0.2980673684
H	4.8666644127	-2.5011942768	-0.0889592340
H	4.8318100997	-0.4574085686	-1.5412819765
H	2.8403782765	1.0349398215	-1.4050168990
H	-2.7578501965	-0.5995709898	-0.2329885082
H	-3.5024509282	2.7658856153	-0.0769089711
H	-6.2822752998	2.1432633456	-0.8283587553
C	-6.2294735631	2.9561520500	1.1458003329
C	-5.5528235849	4.1462013669	-0.9785160297
O	1.0449547340	-1.6265456227	1.7534655459
H	3.0726204943	-2.8819543561	1.3353670778
H	-5.6131234014	3.6173394049	1.7577663873
H	-6.2897566975	1.9875072662	1.6413387193

H	-7.2345119712	3.3768142388	1.0976898286
H	-5.1727757686	4.0166993464	-1.9916944462
H	-4.8917887353	4.8368167019	-0.4501307320
H	-6.5368179678	4.6099556160	-1.0393411816

1a'

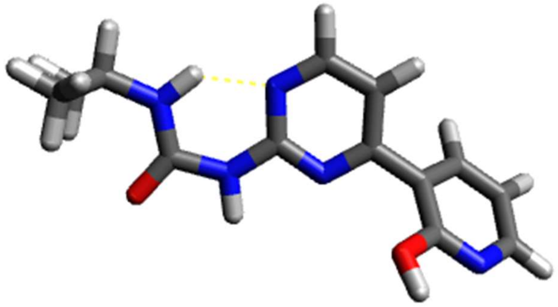

E(a.u.)= -928.0345671

N	-2.6649372973	0.5920012953	0.3368158930
C	-1.5897746391	-0.1334466398	0.0246259327
C	-2.4425904487	1.8858245425	0.5618985075
C	-1.1910363550	2.4603589979	0.4837775698
C	-0.1338790869	1.6092467937	0.1520152616
N	-0.3340052052	0.3137254676	-0.0780315277
C	1.2409464255	2.1353787450	0.0114925907
C	2.3636865442	1.5290351902	0.6065908859
N	3.5993932728	1.9783128042	0.4688125341
C	3.8001126610	3.0707265268	-0.2714534038
C	2.7762138466	3.7674366357	-0.8851258460
C	1.4835313090	3.2850556097	-0.7280969207
N	-1.7310359500	-1.4794205204	-0.2291974175
C	-2.8412353346	-2.3435583267	-0.2669259696
N	-4.0360295554	-1.7991878233	0.0108718386
C	-5.3114708556	-2.5210578149	0.0150704451
O	-2.6371206051	-3.5179852521	-0.5478353728
H	-3.3103846617	2.4821433652	0.8207583381
H	-1.0411764653	3.5093383439	0.6860143324
H	4.8291458107	3.3974444623	-0.3694459869
H	2.9798876341	4.6513220869	-1.4711745227
H	0.6546647561	3.7938929537	-1.2029765670
H	-0.8711847998	-1.9502397998	-0.4526725525
H	-4.0323647877	-0.8122337337	0.2239507318
H	-6.0418131731	-1.7521093407	0.2702995883
C	-5.6750492351	-3.0621634531	-1.3612639113
C	-5.3683871471	-3.5878805528	1.1002294380
O	2.1890983651	0.4559225579	1.3924452824
H	3.0565752031	0.2118706863	1.7349020635
H	-4.9857907992	-3.8456972022	-1.6722835457

H	-5.6556396498	-2.2675846947	-2.1072966994
H	-6.6816486218	-3.4806906354	-1.3389439556
H	-5.1368877966	-3.1604694727	2.0760624272
H	-4.6638400972	-4.3932593186	0.8991736578
H	-6.3709734218	-4.0140525029	1.1469168805

1a"

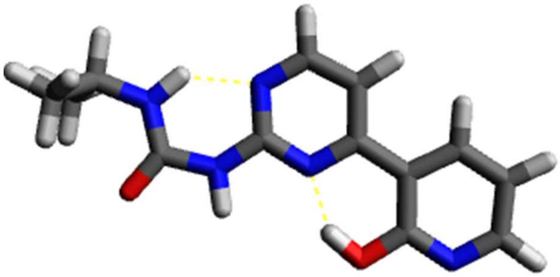

E(a.u.)= -928.0404691

N	-2.7680722852	0.4740240444	0.0206127883
C	-1.6609914983	-0.2626468421	0.0132454206
C	-2.5799380873	1.7949223371	0.0320044633
C	-1.3388062155	2.3900333144	0.0361679923
C	-0.2262650075	1.5401659572	0.0278050663
N	-0.4097209497	0.2131392437	0.0163577104
C	1.1603371263	2.0374098331	0.0309896779
C	2.2903741853	1.1730442123	0.0225055747
N	3.5423384492	1.6133089364	0.0253090540
C	3.7554088454	2.9251303582	0.0365804791
C	2.7396066397	3.8683498280	0.0456543641
C	1.4378401831	3.4041573561	0.0426854976
N	-1.7528538177	-1.6324467927	0.0013461175
C	-2.8457334356	-2.5258085195	-0.0055532621
N	-4.0698579094	-1.9789700206	0.0002522466
C	-5.3278254109	-2.7325330183	-0.0052893403
O	-2.5927414635	-3.7227374234	-0.0162320369
H	-3.4772551492	2.4026854792	0.0380702942
H	-1.2587168901	3.4637763858	0.0454901565
H	4.7939230836	3.2387182369	0.0384551812
H	2.9611604417	4.9251683765	0.0546918319
H	0.6296505233	4.1205580882	0.0496075300
H	-0.8732399309	-2.1195305147	-0.0036498508
H	-4.1049333833	-0.9704196516	0.0091004418
H	-6.0934960916	-1.9558250426	0.0021449136
C	-5.5063325764	-3.5452659722	-1.2804892581
C	-5.5042146539	-3.5674539345	1.2557928219
O	2.1718320187	-0.1516757745	0.0110404421
H	1.2086375978	-0.3624944527	0.0099943009
H	-4.7664150213	-4.3412454422	-1.3460942729

H	-5.4110895414	-2.9099177750	-2.1612069073
H	-6.4985272160	-3.9970898164	-1.2947591831
H	-5.4074900641	-2.9476102055	2.1473309315
H	-4.7642013443	-4.3644495787	1.3062253631
H	-6.4963913238	-4.0194713032	1.2638134501

1b

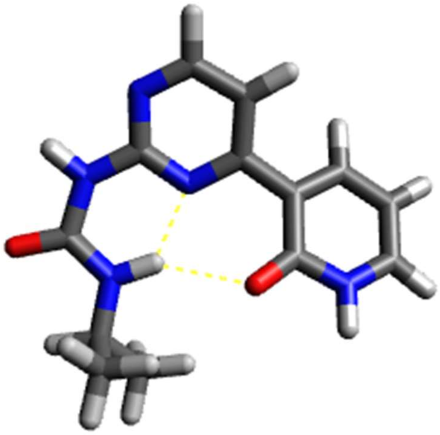

E(a.u.)= -928.0468564

N	1.0310522408	-1.0032044746	0.5202839343
C	0.5039101745	0.0531747074	-0.1140604223
C	0.1483717745	-1.8936832650	0.9543169287
C	-1.2204573014	-1.7654962604	0.7816614750
C	-1.6575878849	-0.6454648818	0.0747581239
N	-0.7826495678	0.2556451859	-0.3747847511
C	-3.0828911046	-0.4204762663	-0.2294248516
C	-3.6103636346	0.9369569829	-0.2958723695
N	-4.9742726263	0.9877329267	-0.6059916156
C	-5.7814432271	-0.0678301642	-0.8416466511
C	-5.2837141652	-1.3315843901	-0.7788698442
C	-3.9193029214	-1.4835272312	-0.4658541426
N	1.4184842761	0.9926620410	-0.5303311422
C	1.2763651733	2.3061658206	-1.0187553054
N	0.0263989791	2.7533639313	-1.2180953727
C	-0.2577021553	4.1231948224	-1.6247321683
O	2.2988589341	2.9454253825	-1.2294379747
H	0.5540294728	-2.7527285645	1.4787802308
H	-1.9005955280	-2.4969068042	1.1900178065
H	-6.8111281260	0.1587011782	-1.0775892594
H	-5.9172021015	-2.1819168228	-0.9738715317
H	-3.5106956841	-2.4855380524	-0.4341996775
H	2.3762793627	0.7608990382	-0.3328388362
H	-0.7408400610	2.1481508022	-0.9513714353
H	0.6558560729	4.4969074861	-2.0844527101
C	-1.3731349541	4.1277122856	-2.6583886553
C	-0.5868794981	4.9903645933	-0.4162662252
O	-3.0134916533	1.9855602549	-0.0970283069
H	-5.3521089994	1.9212661637	-0.6497288689
H	-2.2924852235	3.7162470029	-2.2374392520

H	-1.0988170878	3.5345010792	-3.5308008309
H	-1.5787093538	5.1461015486	-2.9888507649
H	0.2365557666	4.9850484039	0.2978908526
H	-1.4822469437	4.6222936647	0.0880783528
H	-0.7654424245	6.0222818755	-0.7221047392

1b'

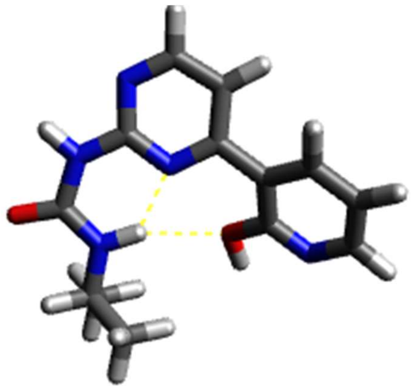

E(a.u.)= -928.0443276

N	-2.6321250615	0.3442851571	-0.3043135470
C	-1.4691410173	-0.2828985143	-0.0832475820
C	-2.5447967510	1.6613723468	-0.4416534242
C	-1.3508664271	2.3614708877	-0.3688758539
C	-0.2041658697	1.6128957602	-0.1170706356
N	-0.2745178423	0.2866959830	0.0298712448
C	1.1193359078	2.2523058510	0.0231216824
C	2.3180337527	1.6707719525	-0.4411231232
N	3.5060767389	2.2367294151	-0.3127440223
C	3.5867010927	3.4229337969	0.2910176315
C	2.4839765412	4.0979746279	0.7808324319
C	1.2434234422	3.4950083414	0.6350771363
N	-1.5714545011	-1.6515430766	0.0384414416
C	-0.6121190653	-2.6723304365	0.1445169785
N	0.6740876875	-2.2962309718	0.2058360746
C	1.7773903358	-3.2478016037	0.2517069683
O	-1.0116161810	-3.8310633768	0.1806707047
H	-3.4750155450	2.1872497828	-0.6273505549
H	-1.3246435766	3.4299630977	-0.5152974694
H	4.5810500108	3.8450374769	0.3827353043
H	2.5904147490	5.0569004016	1.2661088212
H	0.3597416037	3.9854117785	1.0206893327
H	-2.5081721416	-2.0077518695	-0.0394928659
H	0.8688036475	-1.3069209084	0.1198430221
H	1.3799616583	-4.1564077432	0.7023098663
C	2.2762017049	-3.5779965512	-1.1491264466
C	2.8794405297	-2.6974268765	1.1421747735
O	2.2826837165	0.4921110363	-1.0832470863

H	3.1865592900	0.3002179718	-1.3603664108
H	2.6694556116	-2.6855756729	-1.6403606528
H	1.4703354617	-3.9792852457	-1.7634452268
H	3.0738750447	-4.3205319382	-1.1057661464
H	2.5104801414	-2.5064793732	2.1497539965
H	3.2777205246	-1.7628605203	0.7407163864
H	3.7018848739	-3.4092309836	1.2080572503

1c

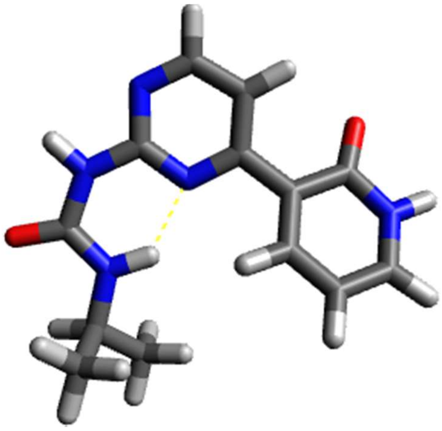

E(a.u.)= -928.0488424

N	-1.7989877893	2.2110831349	0.2468832824
C	-1.6412993119	0.8836903412	0.1519554577
C	-0.6727561048	2.9127343823	0.2303928698
C	0.5859849361	2.3453397529	0.1175245526
C	0.6401634528	0.9561344962	0.0372041932
N	-0.4899111655	0.2306904269	0.0540102102
C	1.9053741421	0.2025771411	-0.0274858115
C	3.1093598835	0.8145216366	-0.5626900996
N	4.2226720060	-0.0265359633	-0.5464022309
C	4.2584928557	-1.2924941942	-0.0840419779
C	3.1302969612	-1.8641856632	0.4197994437
C	1.9563754719	-1.0930490209	0.4341867401
N	-2.8271242592	0.1771243195	0.1632554279
C	-3.1343664407	-1.1856328761	0.0416685646
N	-2.1051890429	-2.0231748986	-0.1587304440
C	-2.2575341895	-3.4701653186	-0.2473211447
O	-4.3118308050	-1.5202242123	0.1100451278
H	-0.7783084783	3.9893757167	0.3110748346
H	1.4766516005	2.9483278193	0.0971072412
H	5.2125028779	-1.7956771679	-0.1391220829
H	3.1492685318	-2.8733595987	0.7988968320
H	1.0531527759	-1.5268582803	0.8412421513
H	-3.6466030047	0.7510843512	0.2617213078
H	-1.1827443525	-1.6118081341	-0.1552274609
H	-3.2573923008	-3.6496090272	-0.6404254872

C	-1.2354797568	-4.0217720754	-1.2273876339
C	-2.1500028068	-4.1203012679	1.1258752565
O	3.2312648236	1.9462049077	-1.0225385141
H	5.0635242362	0.3861902304	-0.9195637403
H	-0.2170707784	-3.8248563897	-0.8842623154
H	-1.3562468962	-3.5749668068	-2.2141082796
H	-1.3500351402	-5.1010092736	-1.3236975821
H	-2.8934788072	-3.7101472771	1.8089379724
H	-1.1602130372	-3.9538961164	1.5557262130
H	-2.3115100874	-5.1963550947	1.0524971261

1c'

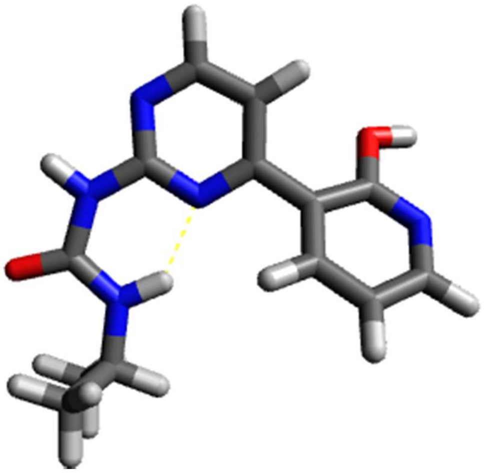

E(a.u.)= -928.0368795

N	-2.6620674974	0.3941581039	0.2302049623
C	-1.4654972583	-0.2053091542	0.1598381710
C	-2.6287480080	1.7198289292	0.2573892182
C	-1.4583362048	2.4597380618	0.2079959641
C	-0.2698589431	1.7403279882	0.1423331236
N	-0.2827127425	0.4001178115	0.1214223381
C	1.0630874290	2.3769726817	0.1444386696
C	1.3435088625	3.6221928602	-0.4518833433
N	2.5356166925	4.1934627375	-0.4410211449
C	3.5369462784	3.5628942064	0.1733916424
C	3.3916053365	2.3321247737	0.7879835963
C	2.1372539055	1.7422982107	0.7599761760
N	-1.5258487366	-1.5814660509	0.1278881112
C	-0.5526132024	-2.5908483235	0.0137737407
N	0.7232835533	-2.1857887545	-0.0835265169
C	1.8835906481	-3.0705402966	-0.2202528307
O	-0.9369152088	-3.7537303780	0.0088388447
H	-3.5876459503	2.2225011784	0.3236525057
H	-1.4776620546	3.5358637304	0.2335175787
H	4.4947506764	4.0709733818	0.1690315160
H	4.2284916074	1.8541352324	1.2754988914

H	1.9736305662	0.7838664539	1.2327464974
H	-2.4591604401	-1.9530902708	0.1702357909
H	0.8700299541	-1.1877520906	-0.0643192506
H	2.7275913297	-2.3816375265	-0.2742787319
C	2.0852364357	-3.9518846338	1.0047919148
C	1.8543900719	-3.8645614516	-1.5192665259
O	0.3674455669	4.2769501720	-1.1013210477
H	0.7560283053	5.0848962179	-1.4562643792
H	1.2730403496	-4.6692658870	1.1116534298
H	2.1364509618	-3.3490161648	1.9116998941
H	3.0207071888	-4.5047023455	0.9127704008
H	1.7441368245	-3.1999919592	-2.3762466853
H	1.0315985159	-4.5773356815	-1.5262233506
H	2.7876450966	-4.4163817596	-1.6364691710

1c''

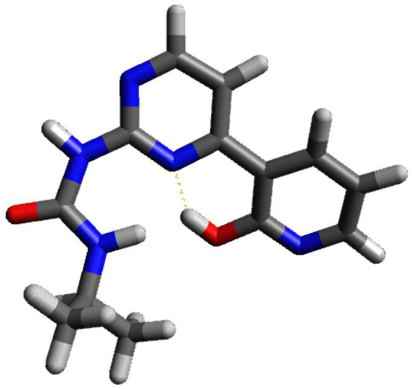

E(a.u.)= -928.319694

N	1.5033206516	3.7355131778	0.1646121525
C	1.2431888811	2.4304542414	0.0288950427
C	2.7400819838	4.0351809068	0.5369550248
C	3.7175413188	3.0870686815	0.7880623399
C	3.3603598396	1.754496532	0.6040425262
N	2.1132340662	1.435583977	0.2115326631
C	4.3189819162	0.6526299756	0.791345358
C	4.2014045043	-0.5909652649	0.1176643476
N	5.0873097924	-1.5675396299	0.2374894998
C	6.1242284266	-1.386594902	1.0492993841
C	6.3358820787	-0.2287153544	1.7813740679
C	5.4178834937	0.7955805207	1.6351980138
N	-0.0466754126	2.1505474986	-0.3504261919
C	-0.8166534423	0.9733081611	-0.4164241516
N	-0.2434616573	-0.1627608682	0.0204035347
C	-0.9621983369	-1.4331919238	0.0805539432
O	-1.9511064434	1.0579620782	-0.8569363078
H	2.9671520996	5.0913280589	0.6341733317
H	4.7148272966	3.385561797	1.0681844819
H	6.8199242315	-2.2158477668	1.1227674981
H	7.1860799541	-0.1353485682	2.4409836173

H	5.545168256	1.7112774045	2.1969977318
H	-0.5939853083	2.9687587447	-0.5575716447
H	0.6606941715	-0.0954917209	0.4546854738
H	-1.6607389867	-1.4212551799	-0.7550039773
C	0.0231739979	-2.5726755885	-0.1129239508
C	-1.7546277809	-1.5615069452	1.3742515341
O	3.1921445839	-0.8584268181	-0.7144313356
H	2.5511423229	-0.1265622029	-0.6606256014
H	0.7616219151	-2.5973416601	0.6917609135
H	0.5574580594	-2.4777258289	-1.0577515149
H	-0.5023348235	-3.5270562413	-0.1093455144
H	-2.4543927028	-0.7337596148	1.4839067085
H	-1.0882815728	-1.5681408889	2.2391731707
H	-2.324593006	-2.491263175	1.3798302234

1d

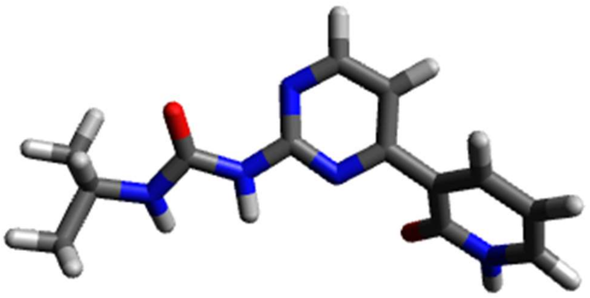

E(a.u.)= -928.0435752

N	-1.6531702696	2.3830318345	-0.9226264860
C	-1.7182934187	1.1625364346	-0.3951551439
C	-0.4225224812	2.8277409579	-1.1651784980
C	0.7194243867	2.0890614688	-0.9186222811
C	0.5305519395	0.8107118797	-0.3942170489
N	-0.6910425050	0.3511437462	-0.1260471724
C	1.6806247543	-0.0626185145	-0.0776621750
C	1.7128387931	-1.4225676778	-0.5836466001
N	2.8302722124	-2.1445370097	-0.1676797189
C	3.8366152283	-1.6782879396	0.6057527783
C	3.8161998588	-0.3936882654	1.0493388998
C	2.7159493254	0.4098380517	0.6861350803
N	-2.9522322693	0.6015229856	-0.1182841552
C	-4.1760387939	1.2445077901	0.1144154886
N	-5.2074067577	0.3668254538	0.2158660594
C	-6.5848083108	0.7925759139	0.4397855709
O	-4.2931342491	2.4480191011	0.2493275936
H	-0.3494483295	3.8217920336	-1.5938782672
H	1.7006713881	2.4752969095	-1.1479460381
H	4.6268162579	-2.3782299860	0.8332053927
H	4.6139351963	-0.0137888302	1.6672211185

H	2.6780422794	1.4311252712	1.0448220674
H	-2.8884432172	-0.3697287028	0.1332104532
H	-5.0652186789	-0.5831346830	-0.0807012928
H	-6.5179262874	1.7159534885	1.0136724588
C	-7.2973976060	1.0867867481	-0.8739097979
C	-7.3094223221	-0.2534528907	1.2696107180
O	0.8972499975	-1.9525772093	-1.3317768524
H	2.8697830416	-3.0932882821	-0.5064779428
H	-7.3747766044	0.1846166859	-1.4843252689
H	-6.7604648438	1.8429740680	-1.4461940464
H	-8.3067186967	1.4548154792	-0.6858887270
H	-6.8065962909	-0.4156510641	2.2226101518
H	-7.3600356818	-1.2081801132	0.7412485136
H	-8.3308770454	0.0678548667	1.4709951683

1d'

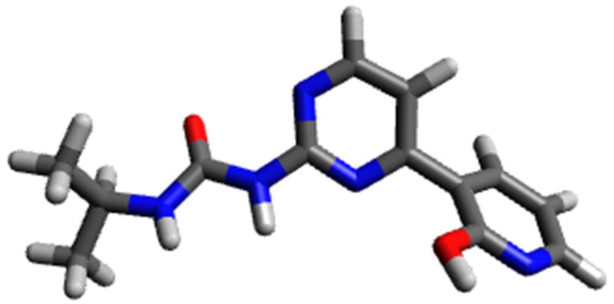

E(a.u.)= -928.0277896

N	-2.6088029678	0.3581129199	0.9460417269
C	-1.5987749673	-0.2981624583	0.3794850502
C	-2.3778030293	1.6443786976	1.1960688217
C	-1.1788715625	2.2736398653	0.9176282220
C	-0.1796464242	1.4805504057	0.3553935543
N	-0.3928439207	0.1950152541	0.0794744018
C	1.1383438237	2.0557445696	0.0072117695
C	2.3595817485	1.4791709376	0.4035044987
N	3.5431702705	1.9714290585	0.0805336316
C	3.5888952740	3.0810123604	-0.6605562042
C	2.4588536688	3.7505698307	-1.0902520165
C	1.2231346145	3.2219940854	-0.7394243265
N	-1.7335136473	-1.6436264894	0.0904151740
C	-2.9078099978	-2.3809972300	-0.1210444606
N	-2.6648015792	-3.7112092855	-0.2422994245
C	-3.7257534311	-4.6896355476	-0.4571543213
O	-4.0116495229	-1.8800070975	-0.2236354161
H	-3.1909743047	2.1954367451	1.6566909184
H	-1.0216961534	3.3154883351	1.1508555491
H	4.5781364422	3.4446565778	-0.9140388320
H	2.5386760979	4.6490972086	-1.6842350412

H	0.3130463617	3.7083591117	-1.0661014120
H	-0.8675536709	-2.0684405764	-0.1931141217
H	-1.7633992991	-4.0652753801	0.0272887493
H	-4.4997361475	-4.1671074500	-1.0179665399
C	-3.1932860927	-5.8354428425	-1.3005432975
C	-4.3193371664	-5.1670075775	0.8618633700
O	2.3438527997	0.3875333389	1.1847604737
H	3.2618002706	0.1641927777	1.3770539862
H	-2.3853187340	-6.3599962537	-0.7854913207
H	-2.8136144733	-5.4758016583	-2.2564955259
H	-3.9851208066	-6.5579067303	-1.4958352880
H	-4.6978348987	-4.3272023617	1.4440927588
H	-3.5688838668	-5.6889325122	1.4591973204
H	-5.1454648293	-5.8556306216	0.6806275720

1d"

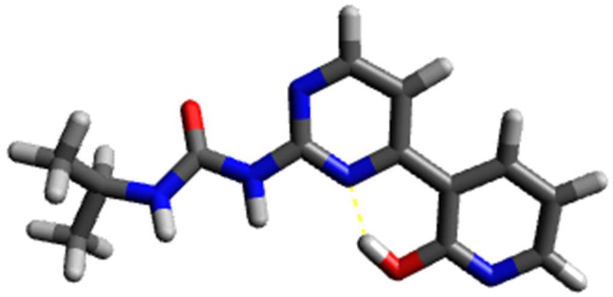

E(a.u.)= -928.0338617

N	-2.7931610318	0.4586003453	0.5827357550
C	-1.7569549470	-0.2586105248	0.1679258795
C	-2.5421926624	1.7546934828	0.7677239908
C	-1.3088001064	2.3411092207	0.5735939702
C	-0.2702447541	1.5037893010	0.1562312074
N	-0.5191808482	0.2033204896	-0.0512850528
C	1.1043587580	1.9815797094	-0.0793512045
C	2.1407136306	1.1370986233	-0.5667219434
N	3.3788378908	1.5592176420	-0.7897355178
C	3.6711085548	2.8318514839	-0.5420451097
C	2.7524512298	3.7507218602	-0.0603068920
C	1.4629018242	3.3063974177	0.1667028270
N	-1.8907611962	-1.6154154213	-0.0299785655
C	-3.0691767227	-2.3639101853	-0.1985520836
N	-2.8417830890	-3.6986553451	-0.1269539856
C	-3.9040677034	-4.6888985803	-0.2754366114
O	-4.1545054820	-1.8665834290	-0.4250423322
H	-3.3801230596	2.3574215330	1.1006939658
H	-1.1811640020	3.3966540140	0.7461504972
H	4.6949973486	3.1325931719	-0.7382286283
H	3.0374454228	4.7749964619	0.1291477456

H 0.7297017351 4.0045488499 0.5429727814
H -1.0199330997 -2.0897788275 -0.1925930090
H -1.9614872427 -4.0218908322 0.2348578786
H -4.6300833902 -4.2438101208 -0.9542275641
C -3.3357566063 -5.9446075133 -0.9143196031
C -4.5901266286 -4.9724532375 1.0539334771
O 1.9394559077 -0.1493635942 -0.8404109476
H 0.9991859514 -0.3536812524 -0.6228532563
H -2.5713172331 -6.3969383350 -0.2788150087
H -2.8894780171 -5.7248180668 -1.8839143063
H -4.1244632524 -6.6820657968 -1.0595845605
H -4.9949901977 -4.0571171502 1.4848481975
H -3.8879687232 -5.4076816356 1.7676363966
H -5.4114383886 -5.6763138449 0.9152016129

1e

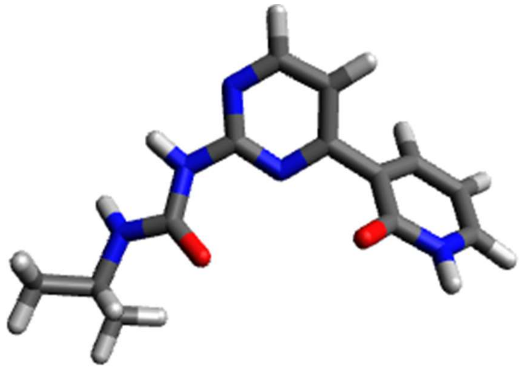

E(a.u.)= -928.0431625
N -1.7306471542 1.7952770703 0.8209181520
C -1.5602424884 0.5020207723 0.5074962342
C -0.6217533653 2.5211380053 0.8740752688
C 0.6373069935 1.9962014815 0.6374363355
C 0.6930904384 0.6441097818 0.3119673117
N -0.4108988682 -0.1052978811 0.2396893352
C 1.9823039960 -0.0016904098 -0.0181696084
C 2.3813550530 -1.2113113282 0.6768240131
N 3.5977309905 -1.7305784183 0.2358289931
C 4.3936337769 -1.1946797611 -0.7171332988

C 4.0293106678 -0.0432037093 -1.3404997557
C 2.8051696357 0.5480517641 -0.9661374666
N -2.7476709340 -0.2009047080 0.4208186296
C -2.9634059266 -1.5856773308 0.4283692372
N -4.2490731173 -1.9061298559 0.1295753288
C -4.7246974304 -3.2839096614 0.0655532849
O -2.1079998781 -2.4034476501 0.7099703477
H -0.7437413584 3.5697794923 1.1237464533
H 1.5240469190 2.6070465914 0.7089542195
H 5.3054566433 -1.7310275212 -0.9342480478
H 4.6573704060 0.3910460756 -2.1016513387
H 2.4952796620 1.4592910023 -1.4627075222
H -3.5551860808 0.3921943406 0.5037073700
H -4.8460499771 -1.1978882131 -0.2608441869
H -4.1094768357 -3.8407321591 0.7706897399
C -6.1730559726 -3.3419580706 0.5211767464
C -4.5305065064 -3.8770630319 -1.3235740886
O 1.7850564879 -1.7679776218 1.5934877760
H 3.8918513473 -2.5725080536 0.7058379218
H -6.8171979928 -2.7599136021 -0.1416967121
H -6.2824689937 -2.9533817702 1.5333768935
H -6.5297853446 -4.3713448339 0.5087163630
H -3.4842959777 -3.8286834030 -1.6241995597
H -5.1258507734 -3.3375946687 -2.0628330731
H -4.8409580414 -4.9222527142 -1.3375212966

1e'

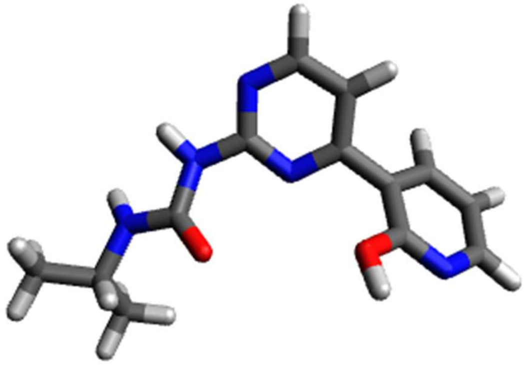

E(a.u.)= -928.0263874
N -2.4368774617 0.1996315664 0.6190205687
C -1.2741131631 -0.3989190265 0.3200360848
C -2.3878774283 1.5197782883 0.7356855740
C -1.2226250440 2.2504476966 0.5737659548
C -0.0785810444 1.5245241427 0.2580384957
N -0.1038364796 0.1958166239 0.1231411764
C 1.2065667550 2.2158293941 0.0084581630
C 2.4020192815 1.8795003858 0.6696978049

N	3.5596651631	2.4759611606	0.4433235598
C	3.6019128165	3.4572644632	-0.4611861722
C	2.4904246435	3.8922536610	-1.1574006805
C	1.2809609504	3.2571923836	-0.9045797347
N	-1.3870157839	-1.7674375172	0.1661419440
C	-0.3853765263	-2.7484627309	0.1511761588
N	-0.8559686041	-3.9565920687	-0.2492686350
C	-0.0658524777	-5.1811429450	-0.2107102257
O	0.7692551661	-2.5398840904	0.4740031412
H	-3.3216204926	2.0174109913	0.9752225084
H	-1.2089961262	3.3229148399	0.6927062016
H	4.5720551377	3.9118946078	-0.6268160753
H	2.5655115047	4.6942862706	-1.8768913379
H	0.3876392000	3.5599886536	-1.4355618101
H	-2.3417866453	-2.0805395365	0.1943708660
H	-1.8164588450	-4.0387777477	-0.5331366597
H	0.9573398539	-4.8574310975	-0.0296311613
C	-0.5103596836	-6.0767802295	0.9374209940
C	-0.1304571210	-5.8865943803	-1.5562164474
O	2.3779803518	0.9266357171	1.6153836233
H	3.2713134321	0.8581762298	1.9712652933
H	-1.5467448072	-6.3941471942	0.8045800282
H	-0.4328749670	-5.5539454347	1.8907223509
H	0.1114223427	-6.9710973176	0.9867504736
H	0.2298488231	-5.2383672394	-2.3545130796
H	-1.1539836581	-6.1854537116	-1.7933343666
H	0.4834909963	-6.7869348638	-1.5416645796

1e"

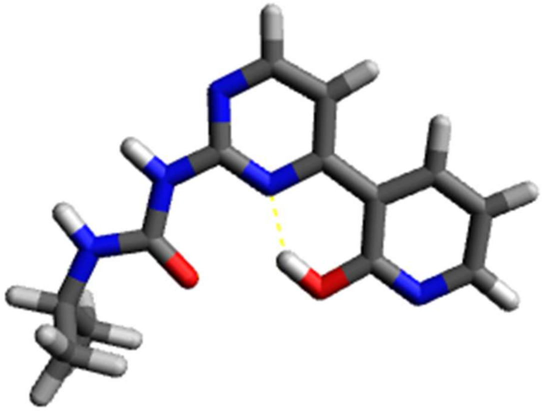

E(a.u.)= -928.0350201

N	-2.6322969504	0.2793270401	0.0000000000
C	-1.4465448726	-0.3378275333	0.0000000000
C	-2.5762225442	1.6077312934	0.0000000000
C	-1.3977092526	2.3300684044	0.0000000000
C	-0.2112089511	1.5948493459	0.0000000000

N	-0.2598669723	0.2551448626	0.0000000000
C	1.1274843930	2.2075607989	0.0000000000
C	2.3254894890	1.4374481353	0.0000000000
N	3.5359180155	1.9890599976	0.0000000000
C	3.6356771170	3.3137657324	0.0000000000
C	2.5436864979	4.1677853958	0.0000000000
C	1.2867951533	3.5926146834	0.0000000000
N	-1.5269500533	-1.7131125196	0.0000000000
C	-0.4921976717	-2.6610573246	0.0000000000
N	-0.9558768042	-3.9339737184	0.0000000000
C	-0.1155369914	-5.1384845967	0.0000000000
O	0.6876763505	-2.3592933907	0.0000000000
H	-3.5290151549	2.1256312006	0.0000000000
H	-1.4275010425	3.4068284710	0.0000000000
H	4.6437368229	3.7154490189	0.0000000000
H	2.6747650791	5.2398456584	0.0000000000
H	0.4181320060	4.2351390353	0.0000000000
H	-2.4765413972	-2.0425870297	0.0000000000
H	-1.9474670785	-4.0841937208	0.0000000000
H	-0.8379802675	-5.9549111595	0.0000000000
C	0.7182717597	-5.2595361863	-1.2681331886
C	0.7182717597	-5.2595361863	1.2681331886
O	2.3341537763	0.1105408633	0.0000000000
H	1.4044995441	-0.2300183083	0.0000000000
H	1.4703993904	-4.4744304035	-1.3214148853
H	0.0872934520	-5.1972755458	-2.1547237286
H	1.2274862763	-6.2234230681	-1.2822877712
H	0.0872934520	-5.1972755458	2.1547237286
H	1.4703993904	-4.4744304035	1.3214148853
H	1.2274862763	-6.2234230681	1.2822877712

1f

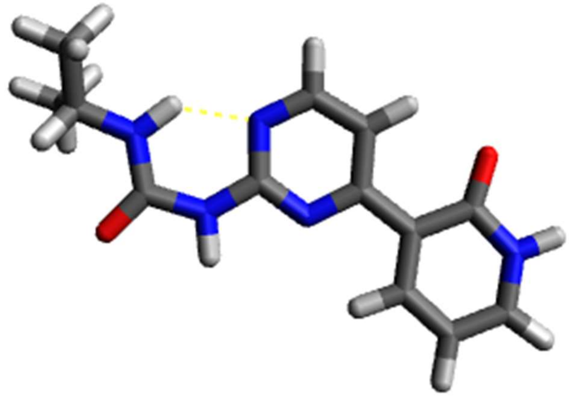

E(a.u.)= -928.0496972

N	-1.5658353571	2.4417705530	0.2197134683
C	-1.5468182843	1.1147715513	0.0751865135

C	-0.3662131838	3.0151536560	0.3021636071
C	0.8231315716	2.3174848526	0.2485408578
C	0.7320598964	0.9284995460	0.0954499257
N	-0.4673047425	0.3412303125	0.0102272306
C	1.9056094066	0.0363348369	0.0080068465
C	3.2583297233	0.5292573239	0.2043305074
N	4.2412540100	-0.4561641528	0.0830638030
C	4.0385294350	-1.7588818973	-0.1908638675
C	2.7707176764	-2.2219088835	-0.3754038090
C	1.7178811472	-1.3014408122	-0.2687522062
N	-2.7412838798	0.4280037980	-0.0208775835
C	-4.0835328490	0.8317559420	-0.0153961560
N	-4.3271038177	2.1421727302	0.1439043548
C	-5.6701614799	2.7086686644	0.1089873674
O	-4.9447129347	-0.0318386968	-0.1444814748
H	-0.3618751973	4.0933563302	0.4180554694
H	1.7741149099	2.8135085547	0.3227811195
H	4.9209218629	-2.3790289253	-0.2505038562
H	2.5933452229	-3.2620688553	-0.5968608139
H	0.7021124257	-1.6431727782	-0.4095770092
H	-2.6380298845	-0.5656150344	-0.1326642479
H	-3.5224250147	2.7515915867	0.1992091704
H	-6.3390868205	1.9376355791	0.4895274336
C	-5.7287222943	3.9118213916	1.0351368966
C	-6.0882872460	3.0537009333	-1.3144289772
O	3.6172647163	1.6756113931	0.4606741930
H	5.1821491114	-0.1208543678	0.2194242643
H	-5.0438325306	4.6953931686	0.7035126430
H	-5.4640401962	3.6353831373	2.0555722317
H	-6.7341382901	4.3314236275	1.0444950211
H	-6.0399849523	2.1746236656	-1.9565685275
H	-5.4356900495	3.8215406567	-1.7340863347
H	-7.1113421108	3.4312806126	-1.3304980610

1f'

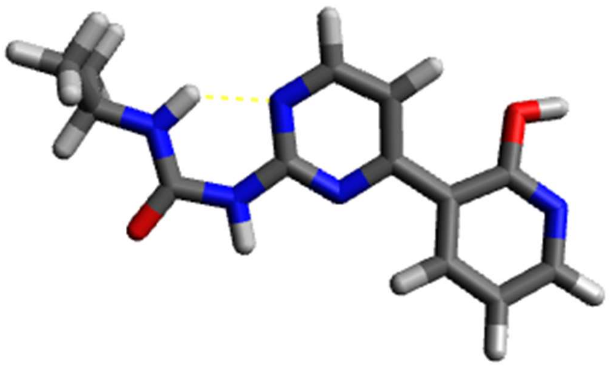

E(a.u.)= -928.0451945

N	-2.7060891565	0.6257955763	0.3337286815
C	-1.6180981925	-0.0543932983	-0.0297423805
C	-2.5080993159	1.9160373375	0.6001180932
C	-1.2783335407	2.5334854890	0.5053637167
C	-0.2034644927	1.7302481681	0.1126808110
N	-0.3829845199	0.4346117638	-0.1509659206
C	1.1861584836	2.2243560637	0.0121858670
C	1.5414285366	3.5515226988	-0.2998196780
N	2.7895111703	3.9828582472	-0.3754579714
C	3.7766772935	3.1179963154	-0.1422433231
C	3.5585826960	1.7856990125	0.1609166090
C	2.2455085108	1.3481484151	0.2288929429
N	-1.7243999006	-1.3971847249	-0.3244736001
C	-2.7961037782	-2.3024740473	-0.2992704580
N	-3.9909496903	-1.8240652672	0.0802842440
C	-5.1955714659	-2.6446138813	0.1242798482
O	-2.5720790048	-3.4661238689	-0.6132987979
H	-3.3824093001	2.4790982721	0.9065814599
H	-1.1622256394	3.5787291764	0.7342824473
H	4.7834248590	3.5155261132	-0.2033241003
H	4.3864725789	1.1156879343	0.3406445859
H	2.0193820553	0.3169370371	0.4605279722
H	-0.8595885730	-1.8308738548	-0.5976447308
H	-4.0440761797	-0.8348918922	0.2811052083
H	-4.8681894046	-3.6594913763	0.3483551865
C	-6.0909754206	-2.1574492482	1.2511148761
C	-5.9113045580	-2.6518886226	-1.2201383804
O	0.5799132708	4.4499958551	-0.5678354355
H	1.0201991422	5.2826250972	-0.7751029804
H	-6.4110319279	-1.1272019341	1.0804569259
H	-5.5753129084	-2.2014598582	2.2102037876
H	-6.9851183164	-2.7766492116	1.3148463068
H	-5.2505556263	-3.0071276181	-2.0104325550
H	-6.2525147586	-1.6485972072	-1.4827961400
H	-6.7817830634	-3.3078727257	-1.1840231182

1g

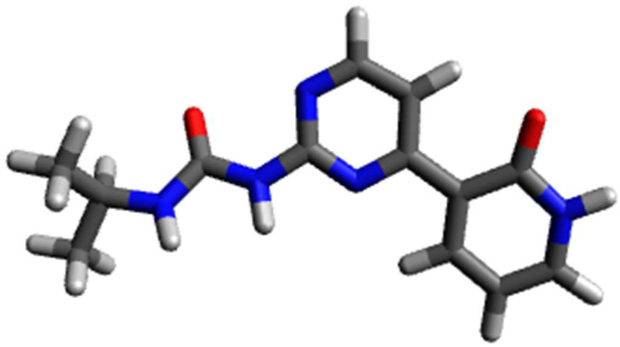

E(a.u.)= -928.0386484

N	-1.6557658244	2.6432157154	0.3900207450
C	-1.6881963359	1.3359489913	0.1379835841
C	-0.4312225262	3.1547103415	0.4940040397
C	0.7313515484	2.4161585718	0.3784119033
C	0.5814019033	1.0476355876	0.1363935833
N	-0.6428768450	0.5211366014	0.0116840551
C	1.7174964247	0.1165856171	-0.0217905584
C	3.0722452768	0.5029359295	0.3345110669
N	4.0208437329	-0.4978083256	0.1150955518
C	3.7850957078	-1.7283815407	-0.3803306231
C	2.5153447505	-2.0913325404	-0.7136874948
C	1.4940915220	-1.1476773785	-0.5224957297
N	-2.9085657081	0.6878081744	0.0347429788
C	-4.1775768876	1.2250485222	-0.2104482794
N	-5.1614181603	0.2933694276	-0.1011151772
C	-6.5730561819	0.6153949674	-0.2797831666
O	-4.3760282838	2.3841760591	-0.5231037275
H	-0.3769685112	4.2202415620	0.6922940641
H	1.7017236297	2.8675132475	0.4825821788
H	4.6439760412	-2.3744026333	-0.4889572089
H	2.3127711554	-3.0718598662	-1.1136032030
H	0.4778331031	-1.4115166562	-0.7803912389
H	-2.8121496897	-0.3110865164	-0.0231588758
H	-4.9456544100	-0.5890719588	0.3289983036
H	-6.6007058084	1.4295134565	-1.0024340100
C	-7.2956259837	-0.5870598888	-0.8631192455
C	-7.2048177603	1.0973649549	1.0194673851
O	3.4548520490	1.5738241341	0.7992776369
H	4.9632354944	-0.2369086552	0.3605311653
H	-7.2537787912	-1.4393755400	-0.1813175270
H	-6.8548735765	-0.8869137968	-1.8136159279
H	-8.3455954673	-0.3499208723	-1.0321040102
H	-6.6722756750	1.9632876961	1.4121443808
H	-7.1855341560	0.3099411116	1.7754766905
H	-8.2445757566	1.3825055002	0.8548366907

1g'

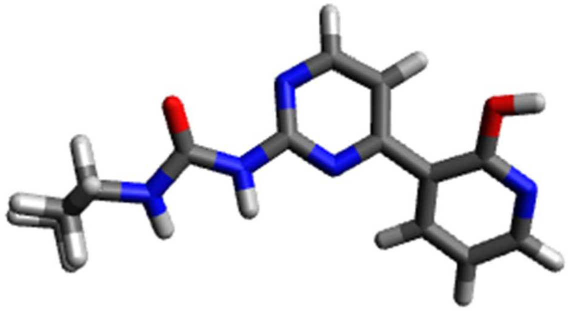

E(a.u.)= -928.0293342

N	-2.9005809769	0.6195123613	-0.3683624289
C	-1.8058228592	-0.1204669075	-0.2167330354
C	-2.6883839119	1.9342740456	-0.3746732526
C	-1.4432260323	2.5173354218	-0.2414981720
C	-0.3606227368	1.6458864485	-0.1083059072
N	-0.5523391436	0.3254138422	-0.0935490128
C	1.0447604015	2.0995585122	-0.0282804930
C	1.4563498206	3.3288505712	0.5231166161
N	2.7158292740	3.7286395605	0.5782214983
C	3.6597046948	2.9282398875	0.0823417581
C	3.3853911780	1.6917310775	-0.4740299277
C	2.0610924638	1.2846878535	-0.5167712262
N	-1.8912103132	-1.5014430716	-0.2091937109
C	-3.0107519096	-2.3335139845	-0.0954267625
N	-2.7047618111	-3.6432211673	-0.2895658896
C	-3.6636501884	-4.7204925302	-0.0692551125
O	-4.1334227298	-1.9326621585	0.1490845386
H	-3.5675175788	2.5569470179	-0.5033611636
H	-1.3229044736	3.5869488528	-0.2604403176
H	4.6769986626	3.2989603925	0.1368176848
H	4.1790557019	1.0711904783	-0.8633225445
H	1.7930626794	0.3268347424	-0.9402229160
H	-0.9855125530	-1.9370693965	-0.1987737561
H	-1.7496906172	-3.9075500845	-0.4559410878
H	-4.6427270984	-4.2926005548	-0.2801299672
C	-3.3957622257	-5.8458146458	-1.0542288316
C	-3.6414767252	-5.1978473260	1.3768077730
O	0.5413459930	4.1555087162	1.0563327547
H	1.0167230922	4.9229152427	1.3955654596
H	-2.4042792320	-6.2777214944	-0.8986069902
H	-3.4575912258	-5.4910160104	-2.0827681303
H	-4.1273646920	-6.6422733305	-0.9220380058
H	-3.8434498863	-4.3738734464	2.0608847979
H	-2.6688301401	-5.6241153063	1.6302927739
H	-4.3994349392	-5.9657536723	1.5360129871

1h

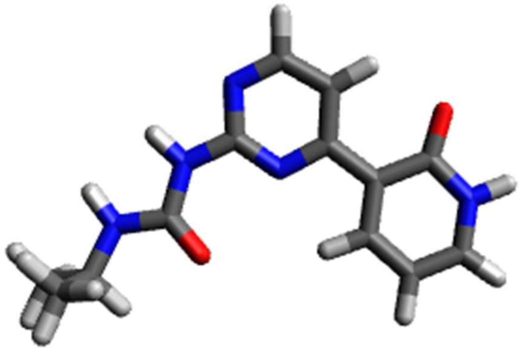

E(a.u.)= -928.0402074

N	-1.8479462907	1.9711766624	-0.4759493145
C	-1.6129096412	0.6651989148	-0.2850501308
C	-0.7650730755	2.7360778879	-0.4519916701
C	0.5205699602	2.2514631420	-0.2644928926
C	0.6377059113	0.8747388822	-0.0749535056
N	-0.4480663368	0.0911986241	-0.0689005273
C	1.9219104383	0.1699915010	0.1183190213
C	3.1708626016	0.8961423465	0.2952270296
N	4.2847022364	0.0558539941	0.4663904293
C	4.2762665414	-1.2947368837	0.4630150117
C	3.1059310348	-1.9631281941	0.2888477171
C	1.9308944711	-1.2045111802	0.1186166353
N	-2.7649778609	-0.1085236590	-0.2872273112
C	-2.8788059898	-1.4962572820	-0.4402528431
N	-4.1832496332	-1.9135966430	-0.3193762527
C	-4.5234176857	-3.3309610091	-0.3451691081
O	-1.9598368002	-2.2402710023	-0.6851242305
H	-0.9301441380	3.7994792654	-0.5956882410
H	1.3811847583	2.8977996314	-0.2550527700
H	5.2306821204	-1.7836954536	0.6017895549
H	3.0862680784	-3.0416414962	0.2804406190
H	0.9799589383	-1.7033105056	-0.0219486074
H	-3.5932007746	0.4456312445	-0.4130156236
H	-4.8255324666	-1.3084395319	0.1613682488
H	-3.8356222364	-3.7781610249	-1.0616283334
C	-4.3025011312	-3.9929870794	1.0087116870
C	-5.9469317725	-3.5000402831	-0.8488124807
O	3.3510619538	2.1025133827	0.3153965250
H	5.1482139784	0.5582503669	0.5920186814
H	-4.9630919127	-3.5651559175	1.7666979658
H	-3.2715250861	-3.8617761918	1.3339691787
H	-4.5080060423	-5.0627673138	0.9520537716
H	-6.0687424852	-3.0585571177	-1.8375671213
H	-6.6646313271	-3.0272105616	-0.1730367563

H -6.2060003362 -4.5567875156 -0.9106243564

1h'

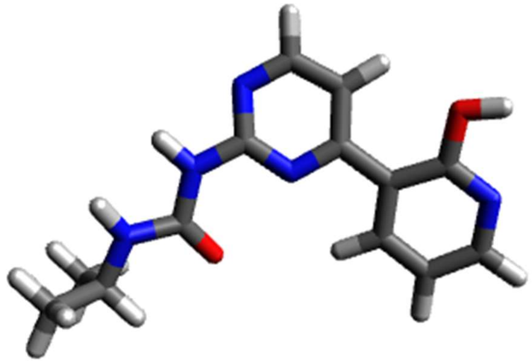

E(a.u.)= -928.0310542

N	-2.5258547000	0.2019425623	-0.1931930552
C	-1.3088908674	-0.3493492731	-0.0599978095
C	-2.5401145507	1.5240755610	-0.2923067084
C	-1.3974993084	2.3054380709	-0.2544906127
C	-0.1893497307	1.6287244401	-0.1104052951
N	-0.1518374299	0.2922013473	-0.0163088340
C	1.1244867283	2.3082878469	-0.0979901389
C	1.3430182196	3.6176108114	0.3742887684
N	2.5230865018	4.2150741922	0.3745822037
C	3.5738798658	3.5484408060	-0.1038714654
C	3.4913718793	2.2549624273	-0.5875542490
C	2.2493709752	1.6399518594	-0.5710036495
N	-1.3575292260	-1.7282551160	0.0447394282
C	-0.3317118158	-2.6722464369	0.1567730442
N	-0.8061247423	-3.9402605530	0.2851030388
C	0.0680844970	-5.1081706717	0.3121811878
O	0.8531249078	-2.3956959195	0.1553767116
H	-3.5143845453	1.9866460413	-0.4101003497
H	-1.4532096162	3.3767615276	-0.3424690214
H	4.5194194135	4.0787405761	-0.0947548428
H	4.3671682963	1.7479390257	-0.9653407977
H	2.1295080475	0.6285136620	-0.9325866697
H	-2.3032257666	-2.0684265819	0.0311763822
H	-1.7910434194	-4.1126873443	0.1859176970
H	1.0009210451	-4.7686414558	0.7601627369
C	-0.5461234101	-6.1763643523	1.2009714079
C	0.3593919217	-5.6199566881	-1.0922371911
O	0.3175865638	4.3186938758	0.8869044481
H	0.6731148024	5.1673495087	1.1755280174
H	-1.5059168121	-6.5133072796	0.8029708889
H	-0.7066218910	-5.8020229163	2.2117027709
H	0.1116293017	-7.0430953449	1.2568292383

H	0.8038412010	-4.8370977182	-1.7065284616
H	-0.5569308713	-5.9601138232	-1.5790348630
H	1.0543647347	-6.4596627104	-1.0550339556

A

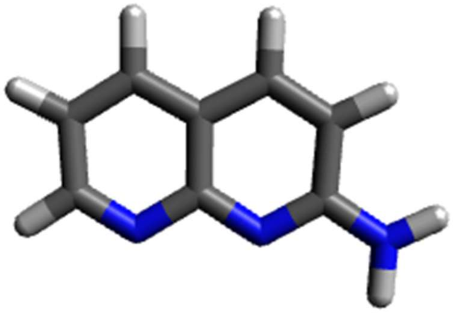

E(a.u.)= -472.9809009

C	-1.8472203635	-1.1050477245	0.0000000000
C	-0.5839008245	-1.7800797992	0.0000000000
C	-1.8537630939	0.2503055296	0.0000000000
C	-0.6266379097	0.9623228076	0.0000000000
C	0.5758976080	0.2030957238	0.0000000000
N	0.5739108039	-1.1498348128	0.0000000000
C	-0.5391254649	2.3617740475	0.0000000000
C	0.6979147270	2.9564119139	0.0000000000
C	1.8268012304	2.1208681452	0.0000000000
N	1.7899937066	0.8045726025	0.0000000000
N	-0.5625335868	-3.1350810096	0.0000000000
H	-2.7669735749	-1.6746492495	0.0000000000
H	-2.7879753317	0.7988895289	0.0000000000
H	-1.4467639873	2.9542119766	0.0000000000
H	0.8147654774	4.0309841155	0.0000000000
H	2.8163041479	2.5697477951	0.0000000000
H	-1.4039422530	-3.6755621560	0.0000000000
H	0.3202486891	-3.6089294344	0.0000000000

B

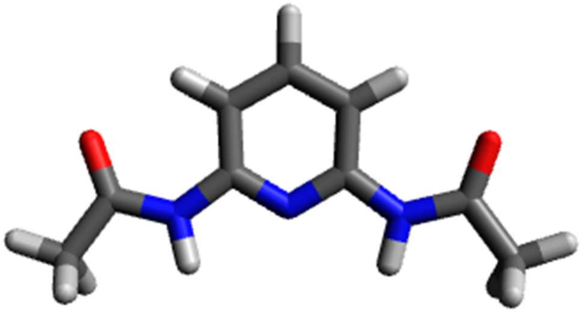

E(a.u.)= -663.9004953

C	-3.4360693399	-0.2153374099	0.0000000000
C	-2.2063671202	-0.8721357100	0.0000000000
C	-3.4034864611	1.1698083727	0.0000000000
C	-2.2040107404	1.8633134015	0.0000000000
C	-1.0375331144	1.0998475414	0.0000000000
N	-1.0399722533	-0.2310941981	0.0000000000
N	-2.0629263209	-2.2625999777	0.0000000000
C	-3.0212885700	-3.2485032028	0.0000000000
C	-2.4477123094	-4.6426123887	0.0000000000
O	-4.2155163979	-3.0246631349	0.0000000000
N	0.2511056526	1.6414896300	0.0000000000
C	0.6559356076	2.9554819073	0.0000000000
C	2.1542528910	3.1215406925	0.0000000000
O	-0.1137037864	3.8956625967	0.0000000000
H	-4.3566971566	-0.7723759974	0.0000000000
H	-4.3344158012	1.7215892465	0.0000000000
H	-2.1572856779	2.9383317594	0.0000000000
H	-1.1004692624	-2.5547297396	0.0000000000
H	-3.2607936058	-5.3608886901	0.0000000000
H	-1.8253002203	-4.8022278186	-0.8809363921
H	-1.8253002203	-4.8022278186	0.8809363921
H	0.9693918309	0.9374099407	0.0000000000
H	2.3940479134	4.1796145886	0.0000000000
H	2.5930572356	2.6521532106	0.8809363995
H	2.5930572356	2.6521532106	-0.8809363995

Complexes

1e" + A

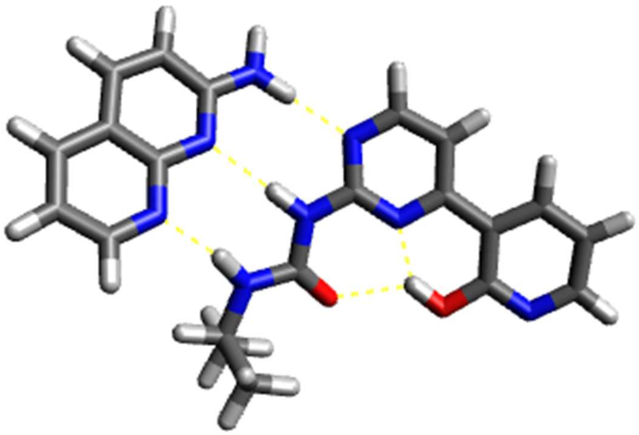

E(a.u.)= -1401.45791

N	-2.5255382245	0.2231987862	-0.9296514761
C	-1.3904001473	-0.4213289149	-0.6191292937
C	-2.4839380055	1.5475380986	-0.8122027164
C	-1.3659630132	2.2519153374	-0.4158015222

C	-0.2275711609	1.4967376299	-0.1145035416
N	-0.2662501130	0.1645944843	-0.2154860047
C	1.0473707014	2.0913637672	0.3176845245
C	2.2496574499	1.3335532273	0.4378775721
N	3.4056940567	1.8793118933	0.8073273807
C	3.4425147451	3.1747613340	1.0882456785
C	2.3381035614	4.0113831468	1.0224455956
C	1.1391705500	3.4468613817	0.6303957388
N	-1.4481911172	-1.7860659046	-0.7107158327
C	-0.3586571768	-2.6838397927	-0.6895789259
N	-0.7622409762	-3.9694718953	-0.6160847156
C	0.1409513935	-5.1209364159	-0.6362387074
O	0.8021799727	-2.3186840015	-0.7544260301
H	-3.3985812069	2.0764368408	-1.0606044421
H	-1.3934248892	3.3278164029	-0.3646875159
H	4.4091017291	3.5686104463	1.3870699038
H	2.4164559885	5.0597995605	1.2704700011
H	0.2584838797	4.0712095230	0.5760302321
H	-2.3758917685	-2.1665191572	-0.8868369460
H	-1.7469392686	-4.1780979723	-0.4803248488
H	-0.5347257329	-5.9781299143	-0.5945015283
C	0.9255983681	-5.2216323873	-1.9380786087
C	1.0381344340	-5.1781525759	0.5943355405
O	2.3217733449	0.0373593399	0.1830667372
H	1.4300776228	-0.3194478433	-0.0424252992
H	1.6269416434	-4.3954536542	-2.0391176845
H	0.2531531786	-5.2119426898	-2.7962054915
H	1.4894952028	-6.1549427738	-1.9597551572
H	0.4439046368	-5.1450115605	1.5079852346
H	1.7368661292	-4.3434593704	0.6075240022
H	1.6112038411	-6.1062578622	0.5959437522
N	-4.3153794134	-3.0046606264	-1.2489182353
C	-5.2662578951	-2.2952535925	-1.8381171914
C	-6.5661542532	-2.8275004320	-2.1254255825
C	-6.8471470462	-4.1014047285	-1.7640732136
C	-5.8549969781	-4.8851429232	-1.1182804940
C	-6.0534148033	-6.2070564902	-0.6993623182
C	-5.0264982585	-6.8812375119	-0.0833417993
C	-3.8118435073	-6.2051457229	0.0903615198
N	-3.5852879019	-4.9629964917	-0.2914058192
C	-4.5898286363	-4.2806511865	-0.8894374808
N	-4.9808786151	-1.0252348951	-2.1985173769
H	-5.7252647098	-0.4277116727	-2.5025906689
H	-4.1464805309	-0.5890259004	-1.8166427282
H	-7.3009173098	-2.2056841573	-2.6186439144
H	-7.8219110537	-4.5308580207	-1.9597794750
H	-7.0133686633	-6.6811267342	-0.8660106112
H	-5.1364963531	-7.9006468643	0.2576754418

H	-2.9813937002	-6.7167385634	0.5684643426

1h + A

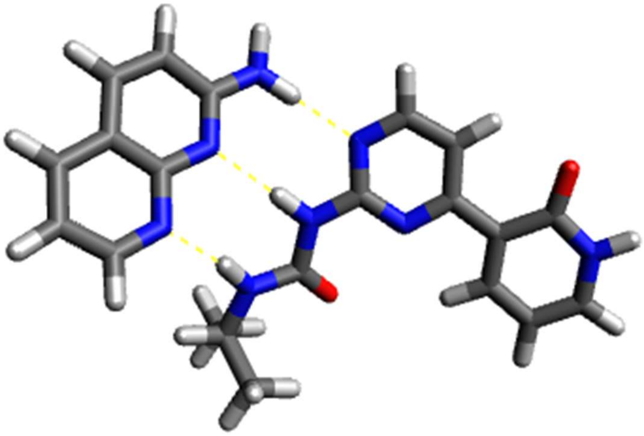

E(a.u.)= -1401.459175

N	-2.5411714726	0.2709078294	-1.1946239690
C	-1.4393209215	-0.3575122975	-0.7437786318
C	-2.5185363558	1.5949384822	-1.1053937687
C	-1.4470499599	2.3146869257	-0.6088092063
C	-0.3569544313	1.5665415039	-0.1610520461
N	-0.3743402101	0.2324276214	-0.2171418456
C	0.8923003967	2.1562623628	0.3714819609
C	1.0235906747	3.4563133435	0.8978646441
N	2.1580469083	3.9577643292	1.3574034328
C	3.2506120721	3.1956753627	1.3230424088
C	3.2513055813	1.8993039509	0.8402862555
C	2.0536509306	1.3870429544	0.3682215144
N	-1.4866033626	-1.7285758615	-0.8116705563
C	-0.3860598607	-2.6093609818	-0.8452380194
N	-0.7643924119	-3.8899882556	-0.6247587478
C	0.1213031380	-5.0484299708	-0.7230315238
O	0.7527068338	-2.2472360059	-1.0779606283
H	-3.4035546380	2.1140595409	-1.4608262783
H	-1.4683965174	3.3896421088	-0.5750709886
H	4.1600780181	3.6495413016	1.7014238129
H	4.1575155323	1.3112398930	0.8324603751
H	1.9875661193	0.3784065836	-0.0156661671
H	-2.4090329216	-2.1227673141	-0.9823179459
H	-1.7334984547	-4.0851199073	-0.3965527930
H	-0.5389529301	-5.8963010783	-0.5266873624
C	0.6931248304	-5.2303431467	-2.1236103368
C	1.2015552358	-5.0467542218	0.3519167068
O	-0.0507521029	4.2622239224	0.9758219826
H	0.2511418921	5.0819292418	1.3839615746
H	1.3723042577	-4.4184262307	-2.3776117912

H	-0.1048143024	-5.2582497367	-2.8660145547
H	1.2446122247	-6.1697733118	-2.1824738446
H	0.7593184277	-4.9531179740	1.3440892583
H	1.8944301366	-4.2199376391	0.2051143449
H	1.7655802199	-5.9800527743	0.3180090023
N	-4.3142193311	-2.9811691050	-1.2782383139
C	-5.2508427583	-2.3347471009	-1.9556101518
C	-6.5414112292	-2.8994212830	-2.2236570634
C	-6.8274507699	-4.1338636780	-1.7468411254
C	-5.8502147620	-4.8466124557	-1.0033418853
C	-6.0568151218	-6.1217290731	-0.4620146552
C	-5.0444618245	-6.7282477797	0.2417390820
C	-3.8344681537	-6.0341490085	0.3764445109
N	-3.6002032694	-4.8343293021	-0.1189645947
C	-4.5924920205	-4.2175581669	-0.8040379945
N	-4.9585200307	-1.1027675841	-2.4253858314
H	-5.6952866958	-0.5402901993	-2.8054485078
H	-4.1324050955	-0.6285409605	-2.0696323180
H	-7.2651026856	-2.3324144218	-2.7936091600
H	-7.7957017027	-4.5854611797	-1.9246422461
H	-7.0119213962	-6.6134737210	-0.6040130121
H	-5.1613380598	-7.7094944732	0.6787235414
H	-3.0144576700	-6.4936910583	0.9207234571

1h’ + A

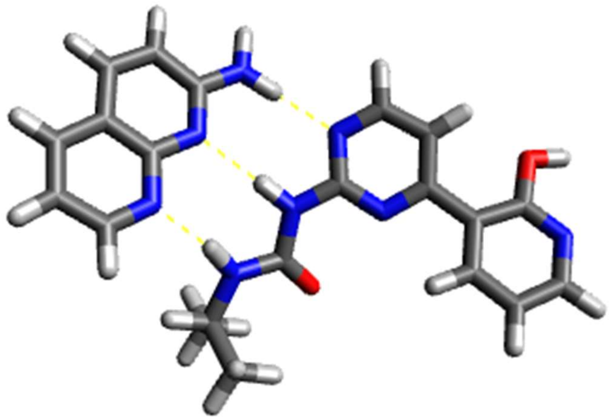

E(a.u.)= -1401.449063

N	-2.5134594520	0.2373245444	-1.0780824280
C	-1.3826314874	-0.3864923747	-0.6958079223
C	-2.5101503663	1.5566251857	-0.9390510363
C	-1.4269569875	2.2791887891	-0.4731231974
C	-0.2997048726	1.5383928266	-0.1127339320
N	-0.3005034052	0.2059782149	-0.2102364698
C	0.9420494219	2.1318996301	0.4370563454
C	1.3111342949	3.4887032163	0.3395705447
N	2.4282769617	3.9909207144	0.8398601186

C	3.2645497342	3.1744676095	1.4782944206
C	3.0235869955	1.8210270656	1.6336344122
C	1.8523603544	1.3105760006	1.0988016608
N	-1.4230948641	-1.7562231324	-0.7963840390
C	-0.3255690098	-2.6386940016	-0.8701260889
N	-0.7089969340	-3.9265217753	-0.7035907676
C	0.1680211035	-5.0836674295	-0.8716039763
O	0.8141677637	-2.2723490223	-1.0873191346
H	-3.4231836065	2.0707889439	-1.2234716503
H	-1.4712730386	3.3502388530	-0.3895260391
H	4.1644779981	3.6299766883	1.8768043052
H	3.7286311307	1.1879053952	2.1524550856
H	1.6155586706	0.2591827092	1.1773756103
H	-2.3487149146	-2.1467142903	-0.9563432658
H	-1.6749460568	-4.1284118399	-0.4681038629
H	-0.4967062753	-5.9361363560	-0.7145351704
C	0.7250826339	-5.1936255699	-2.2858130991
C	1.2594582390	-5.1490184847	0.1900747505
O	0.5184282571	4.3595778940	-0.3103691640
H	0.9657776251	5.2131529525	-0.2789423602
H	1.4054847088	-4.3721225170	-2.5031772536
H	-0.0800508292	-5.1794281002	-3.0207546602
H	1.2714001518	-6.1308806116	-2.3997013939
H	0.8291975495	-5.0969692846	1.1905637533
H	1.9643543104	-4.3272876475	0.0755300432
H	1.8085821354	-6.0879827177	0.1055100549
N	-4.2735046703	-3.0055244490	-1.2286916421
C	-5.2303319639	-2.3394132531	-1.8570435858
C	-6.5312728627	-2.8932746789	-2.0955964969
C	-6.8065139401	-4.1378430719	-1.6396572725
C	-5.8077195847	-4.8720400374	-0.9470714881
C	-6.0014677952	-6.1589931442	-0.4288782264
C	-4.9676891422	-6.7862040476	0.2236904508
C	-3.7502292114	-6.1006376615	0.3315926775
N	-3.5280058691	-4.8899255107	-0.1425184008
C	-4.5410337440	-4.2525500767	-0.7763061598
N	-4.9491792627	-1.0965601908	-2.3053710165
H	-5.6974934153	-0.5220125596	-2.6424630411
H	-4.1142217896	-0.6328765606	-1.9562846628
H	-7.2712731537	-2.3102182256	-2.6270109144
H	-7.7821733762	-4.5815864512	-1.7953811668
H	-6.9634023603	-6.6434436015	-0.5485837306
H	-5.0738313663	-7.7773192214	0.6406891805
H	-2.9132944326	-6.5769793362	0.8341513025

1b + B

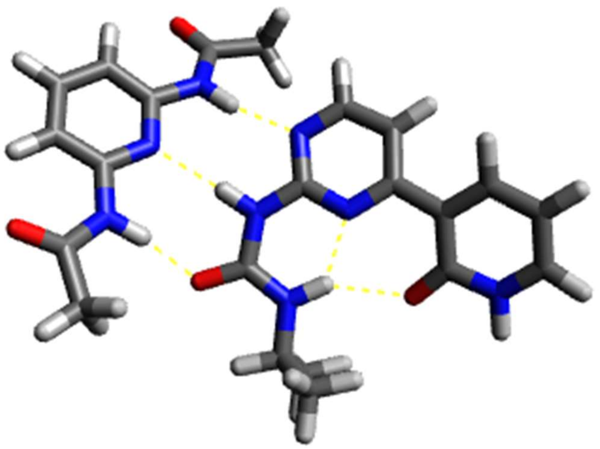

E(a.u.)= -1592.443535

N	-2.6570770473	0.2569458838	-0.4292586123
C	-1.4879331724	-0.3897633693	-0.3081088215
C	-2.5953182926	1.5719229468	-0.2436563829
C	-1.4251718016	2.2536677133	0.0342032639
C	-0.2651713136	1.4841254962	0.1473371157
N	-0.3165711411	0.1623114878	-0.0163823445
C	1.0430218619	2.0791706765	0.4721406486
C	2.2702047751	1.4623638344	-0.0170707678
N	3.4290503141	2.1351532046	0.3859487720
C	3.4854439300	3.2411591855	1.1556463596
C	2.3377674048	3.8127303238	1.6103753343
C	1.1189645752	3.2094433973	1.2504097377
N	-1.5497520971	-1.7480998504	-0.5006257894
C	-0.5394774640	-2.7048071049	-0.6700124203
N	0.7315371893	-2.2877619445	-0.6700620360
C	1.8621683330	-3.1813624450	-0.8907589485
O	-0.8904783272	-3.8757044262	-0.8216894538
H	-3.5304060923	2.1138068861	-0.3392740044
H	-1.4248440446	3.3278400958	0.1314812040
H	4.4717986708	3.6216632011	1.3774929629
H	2.3712444317	4.6916860970	2.2336720178
H	0.2034909765	3.6468334757	1.6274452865
H	-2.4824569830	-2.1352578903	-0.6076262720
H	0.9108699771	-1.3004610712	-0.5274948596
H	1.4495570731	-4.1880532620	-0.9123960651
C	2.5155985110	-2.8848424097	-2.2338399239
C	2.8453614442	-3.0719270539	0.2656018971
O	2.3853061327	0.4733920104	-0.7276997528
H	4.2839944861	1.7198086948	0.0499733029
H	2.8994390006	-1.8634157776	-2.2575258392
H	1.7992061249	-3.0018306725	-3.0472237241
H	3.3471422290	-3.5679426376	-2.4115982387
H	2.3633155182	-3.3199886971	1.2113054415
H	3.2452361906	-2.0589452510	0.3379620807

H	3.6810664093	-3.7567771812	0.1183606544
C	-5.8524987808	-4.9379401011	-0.0196907734
C	-4.6062775397	-4.3253207378	-0.1600641346
N	-4.4625161218	-3.0758246939	-0.6157677259
C	-5.5542722980	-2.3908673709	-0.9654718000
C	-6.8425671163	-2.9132780068	-0.8694469793
C	-6.9663210994	-4.2037996916	-0.3846416599
N	-5.2970026223	-1.0890809586	-1.4120803300
C	-6.1160637658	-0.2711422055	-2.1477517719
C	-5.5224790587	1.0786094131	-2.4647932631
O	-7.2361824660	-0.5772276188	-2.5135042586
N	-3.4037978492	-4.9706032743	0.1294261482
C	-3.2075131461	-6.1561705528	0.7941715936
C	-1.7569079774	-6.5487014938	0.9162206027
O	-4.1048623460	-6.8335908324	1.2625700433
H	-5.9253313623	-5.9424163876	0.3577496777
H	-7.6942081730	-2.3276077533	-1.1672263167
H	-7.9498838203	-4.6462435986	-0.2912922208
H	-4.3801954472	-0.7263967188	-1.1705865252
H	-5.9752395991	1.4517797280	-3.3788495585
H	-5.7738030672	1.7695255917	-1.6574350025
H	-4.4401801493	1.0530860250	-2.5708136389
H	-2.5672608689	-4.5002355048	-0.2092229177
H	-1.6973034942	-7.5439226380	1.3448119025
H	-1.2568656857	-6.5257421417	-0.0508013389
H	-1.2335939274	-5.8449720435	1.5644384255

1b’ + B

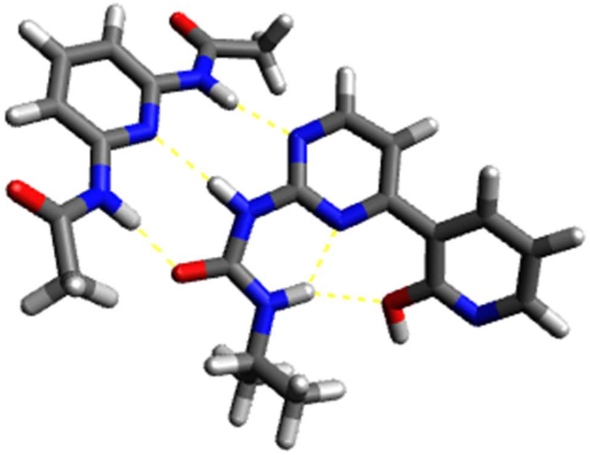

E(a.u.)= -1592.43515

N	-2.6519236391	0.2128694389	-0.2947880569
C	-1.4730756654	-0.4255574192	-0.2348881597
C	-2.6045592888	1.5186646741	-0.0590333454
C	-1.4341471835	2.2051245096	0.2126770470
C	-0.2621489801	1.4517631801	0.2321965906

N	-0.2944240750	0.1358024578	0.0144454027
C	1.0475454929	2.0699183897	0.5173246583
C	2.2431223435	1.6844170650	-0.1240521641
N	3.4203228387	2.2292468050	0.1284499851
C	3.4933281279	3.1944976037	1.0443042911
C	2.3924358292	3.6644026059	1.7371090079
C	1.1619624833	3.0889435318	1.4566333178
N	-1.5408162299	-1.7800644209	-0.4519017837
C	-0.5417969608	-2.7522183743	-0.5840834459
N	0.7348749030	-2.3549023358	-0.5204519529
C	1.8577479706	-3.2667116454	-0.7057937008
O	-0.8975546120	-3.9179218360	-0.7533622228
H	-3.5498715982	2.0492269065	-0.1037376618
H	-1.4397100424	3.2725725860	0.3678167249
H	4.4801977290	3.6045485638	1.2265956626
H	2.4933251652	4.4462559294	2.4752229773
H	0.2805644588	3.4186239393	1.9905937936
H	-2.4775656600	-2.1597196881	-0.5574532233
H	0.9053237113	-1.3666620410	-0.3894047739
H	1.4912539297	-4.2580379321	-0.4429475365
C	2.3085384121	-3.2851723562	-2.1602920553
C	2.9802021184	-2.8849015522	0.2450875930
O	2.2134134292	0.7356593269	-1.0735643486
H	3.1107481676	0.6668761074	-1.4204243080
H	2.6678110961	-2.3006782559	-2.4674845464
H	1.4856864419	-3.5685367962	-2.8160548062
H	3.1186761538	-4.0016281459	-2.3008720925
H	2.6448195849	-2.9170071665	1.2814641363
H	3.3468076068	-1.8775000163	0.0361274172
H	3.8178525110	-3.5733235302	0.1355031907
C	-5.8827773958	-4.9531468027	-0.1606622762
C	-4.6314596841	-4.3385344575	-0.2036570706
N	-4.4541293544	-3.0861876378	-0.6383874517
C	-5.5179577051	-2.3976227090	-1.0595831905
C	-6.8094169230	-2.9212395524	-1.0610577829
C	-6.9679464974	-4.2161578768	-0.5995095115
N	-5.2316461607	-1.0909194237	-1.4717844267
C	-5.9917946080	-0.2711984185	-2.2674664443
C	-5.3859299188	1.0876753672	-2.5142094207
O	-7.0722597028	-0.5838536136	-2.7326756166
N	-3.4511963330	-4.9890193135	0.1647446247
C	-3.3085447684	-6.1461820335	0.8878259328
C	-1.8815910879	-6.5866167570	1.0904409239
O	-4.2398169415	-6.7831194552	1.3479332926
H	-5.9834079575	-5.9613210188	0.2001575392
H	-7.6382159006	-2.3328245336	-1.4127383078
H	-7.9552777177	-4.6595602298	-0.5831767904
H	-4.3405178627	-0.7270135525	-1.1499123134

H	-5.7913334630	1.4877449680	-3.4387461905
H	-5.6766116537	1.7547160510	-1.7001339059
H	-4.2995357642	1.0639840136	-2.5659424692
H	-2.5959528299	-4.5400700080	-0.1518261797
H	-1.7793029063	-7.5976820321	0.6974375177
H	-1.1510242706	-5.9367008296	0.6170430906
H	-1.6893191629	-6.6350202528	2.1619248166

1c + B

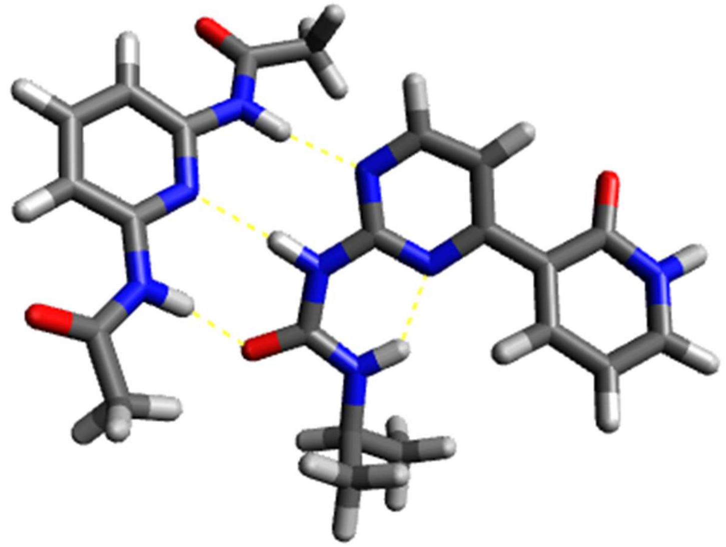

E(a.u.)= -1592.445608

N	-2.6835441230	0.2250565638	-0.2994079743
C	-1.4879385700	-0.3806582591	-0.3596464821
C	-2.6510058146	1.5125970701	0.0234487225
C	-1.4846767857	2.2162768331	0.2655963193
C	-0.2932261348	1.5019519476	0.1599275239
N	-0.3104675313	0.1969890451	-0.1543086003
C	1.0300492637	2.0979445205	0.4158881496
C	1.2360667307	3.5336284001	0.3013154590
N	2.5487777089	3.9344204822	0.5725111444
C	3.5706492880	3.1314530311	0.9302285627
C	3.3720844842	1.7899246716	1.0474034010
C	2.0851380877	1.2934341298	0.7792707417
N	-1.5505165475	-1.7230867647	-0.6619977256
C	-0.5565654532	-2.6748366683	-0.8992958963
N	0.7149224999	-2.2527646889	-0.9393233308
C	1.8472952181	-3.1549841637	-1.1144922534
O	-0.9019033219	-3.8415472751	-1.0786515203
H	-3.6107701847	2.0144295361	0.0866872865
H	-1.4976205119	3.2633845024	0.5106553350
H	4.5195076333	3.6148655217	1.1119472972
H	4.1802174331	1.1395222601	1.3415349480

H	1.9043909084	0.2313756439	0.8758160555
H	-2.4879176653	-2.1131332773	-0.7055897192
H	0.8715843260	-1.2817908000	-0.7110300728
H	1.4942001956	-3.9730722461	-1.7412882130
C	2.9597308610	-2.4187955091	-1.8414698329
C	2.3075115395	-3.7277804356	0.2194980625
O	0.4146577779	4.3861293867	-0.0090682534
H	2.7023704399	4.9272187671	0.4891747691
H	3.3142455773	-1.5663541613	-1.2565419672
H	2.6213020906	-2.0512293505	-2.8096826041
H	3.8075299065	-3.0831582523	-2.0049876152
H	1.4930720148	-4.2525454184	0.7182145280
H	2.6608274120	-2.9338310217	0.8811483851
H	3.1254919482	-4.4334089158	0.0702777630
C	-5.7967204198	-4.9782907364	-0.0056360922
C	-4.5628923157	-4.3533608320	-0.1860960638
N	-4.4460798308	-3.0864490948	-0.5998897712
C	-5.5570391300	-2.3942671676	-0.8644749290
C	-6.8363757943	-2.9278566465	-0.7222317890
C	-6.9310765963	-4.2364390517	-0.2831358728
N	-5.3317266706	-1.0725443685	-1.2657569251
C	-6.1789511663	-0.2483038372	-1.9624928637
C	-5.6257934728	1.1301174770	-2.2227934206
O	-7.2913847510	-0.5733121670	-2.3347942260
N	-3.3422548194	-5.0025151303	0.0133558704
C	-3.1071881152	-6.2081125933	0.6244916886
C	-1.6622238409	-6.6374226205	0.6292051491
O	-3.9742662986	-6.8981316049	1.1313570000
H	-5.8456001085	-5.9971513929	0.3355435427
H	-7.7047286077	-2.3352054346	-0.9500896441
H	-7.9068486315	-4.6872987186	-0.1556892774
H	-4.4174730056	-0.7018024066	-1.0253465515
H	-6.1151947438	1.5430831508	-3.0999242865
H	-5.8631224532	1.7697036203	-1.3703968873
H	-4.5470708475	1.1350010209	-2.3631760875
H	-2.5326994630	-4.5188264600	-0.3655509736
H	-1.3727602893	-6.8505070574	1.6574714480
H	-1.5855641654	-7.5707909549	0.0713019375
H	-0.9854351664	-5.9049421003	0.1979866265

1c' + B

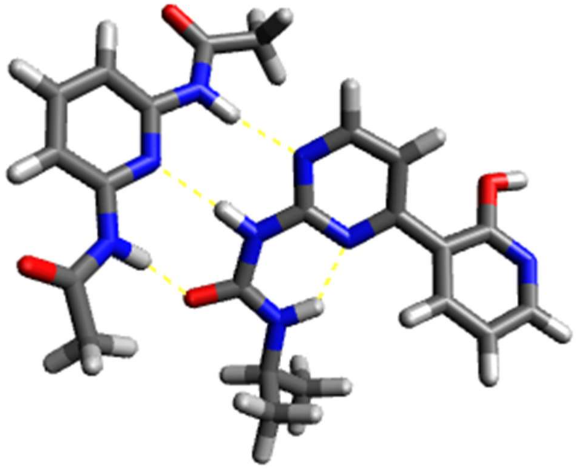

E(a.u.)= -1592.436485

N	-2.6980833742	0.2284189113	-0.2222819636
C	-1.5009133097	-0.3707623228	-0.3039060605
C	-2.6709641847	1.5105810930	0.1206411716
C	-1.5053061492	2.2175778345	0.3571147686
C	-0.3130631452	1.5124061762	0.2206437442
N	-0.3233594118	0.2131994041	-0.1048224952
C	1.0159965646	2.1072567619	0.4646066848
C	1.3360136765	3.4564063178	0.2136265075
N	2.5280856373	3.9844349592	0.4313518705
C	3.4882628462	3.2048453554	0.9256021833
C	3.3017404194	1.8633519402	1.2108339710
C	2.0495575150	1.3223034325	0.9671725514
N	-1.5551144029	-1.7080561693	-0.6204766668
C	-0.5531848538	-2.6500391892	-0.8739282122
N	0.7154892842	-2.2206542626	-0.9035065664
C	1.8524574480	-3.1117315681	-1.1066185422
O	-0.8935541811	-3.8144078715	-1.0743430080
H	-3.6332463182	2.0037928225	0.2069654107
H	-1.5298199065	3.2582079112	0.6311007496
H	4.4466988335	3.6811063053	1.0984226143
H	4.1056955812	1.2656733413	1.6147455589
H	1.8529139586	0.2807059940	1.1815453229
H	-2.4898288474	-2.1042400984	-0.6696116239
H	0.8694513134	-1.2528306591	-0.6591872310
H	1.4938171300	-3.9283847393	-1.7319493108
C	2.9442475079	-2.3608933801	-1.8497637637
C	2.3421268658	-3.6893939146	0.2147348520
O	0.4035102467	4.2724919195	-0.3026728134

H	0.8221184883	5.1321082560	-0.4270620807
H	3.2985869324	-1.5056689406	-1.2690613955
H	2.5851093903	-1.9949927658	-2.8110757491
H	3.7966321751	-3.0151429722	-2.0294173460
H	1.5412933067	-4.2228826099	0.7262616063
H	2.7036563269	-2.8975275355	0.8744128901
H	3.1617313392	-4.3884939966	0.0451795563
C	-5.7884807743	-5.0174323361	-0.1157723740
C	-4.5591664385	-4.3706263418	-0.2419679977
N	-4.4487189730	-3.0900574297	-0.6130840994
C	-5.5620428466	-2.4042288745	-0.8862410738
C	-6.8370315684	-2.9596987406	-0.7980172937
C	-6.9248233299	-4.2829125668	-0.4031204757
N	-5.3417323521	-1.0679661637	-1.2392898700
C	-6.1936482539	-0.2136019050	-1.8930467717
C	-5.6376578379	1.1720693464	-2.1052411567
O	-7.3116521299	-0.5182157190	-2.2653433739
N	-3.3362806721	-5.0110957115	-0.0288073566
C	-3.1020920844	-6.2209549548	0.5745047988
C	-1.6533647924	-6.6361516650	0.6057322549
O	-3.9727632599	-6.9237567923	1.0567315743
H	-5.8326757627	-6.0470269023	0.1921645723
H	-7.7070612046	-2.3723997178	-1.0327484636
H	-7.8972029737	-4.7508601223	-0.3189676332
H	-4.4294040924	-0.7040209652	-0.9837807098
H	-6.1356924211	1.6202817411	-2.9599878501
H	-5.8627641641	1.7788579367	-1.2260141469
H	-4.5607726302	1.1783598448	-2.2586084953
H	-2.5240881003	-4.5146935021	-0.3845116833
H	-1.3797360150	-6.8424701362	1.6397963249
H	-1.5577778667	-7.5707383480	0.0530648686
H	-0.9761241582	-5.8984257144	0.1842792467

1c’’ + B

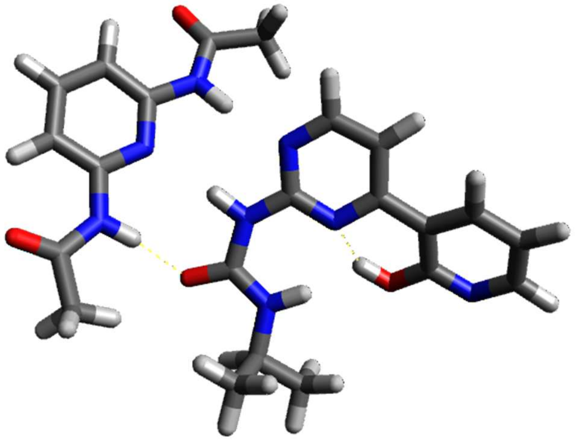

E(a.u.)= -1592.4341237

N	1.518686353	3.7417217763	0.0840147128
C	1.2333654796	2.436995134	-0.0124745861
C	2.7489706268	4.0338660845	0.4888843654
C	3.7034659681	3.0800195924	0.787764295
C	3.3399375165	1.7496587609	0.5951228168
N	2.0966287104	1.4389345629	0.1868661153
C	4.2915157446	0.6452173601	0.793223937
C	4.2052520398	-0.5809848698	0.0851800641
N	5.0855107581	-1.5597485225	0.2203237569
C	6.0866229621	-1.3979470695	1.0797630802
C	6.2674237592	-0.2561009276	1.8449385988
C	5.3553054877	0.7708000253	1.683951028
N	-0.0650846154	2.1660812637	-0.3614204341
C	-0.817845395	0.9837751111	-0.3817097517
N	-0.2257883008	-0.1551856397	0.0028606537
C	-0.9441083041	-1.4259341299	0.0926329688
O	-1.9866769555	1.0590448182	-0.7473529831
H	2.9895626538	5.0886487817	0.5663039727
H	4.6950887547	3.3708389749	1.0941662967
H	6.7784385416	-2.2292059563	1.1643736367
H	7.0894497199	-0.177245598	2.5409755202
H	5.4580059923	1.6738053387	2.2707298942
H	-0.6394063184	2.9871213178	-0.5336088905
H	0.7098295263	-0.093370943	0.3669703899
H	-1.6856330459	-1.4092837434	-0.704816547
C	0.0283028494	-2.5648662636	-0.1589182167
C	-1.6665228873	-1.559221016	1.4259215634
O	3.2360113816	-0.828323173	-0.8005518286
H	2.5895395633	-0.1039507611	-0.7606654296
H	0.8107682914	-2.5926600133	0.6025143061
H	0.5066209443	-2.4699347658	-1.1332183298
H	-0.4982709957	-3.5180471685	-0.1274412354
H	-2.3576012515	-0.7306067353	1.5779394515
H	-0.955684494	-1.5733623559	2.2542427822
H	-2.2379266927	-2.4874676374	1.455216419
H	-4.3717287574	7.7660914489	-0.9799641366
C	-3.7145816434	6.9087840379	-0.9121963599
H	-5.2738492996	5.5429865282	-0.3118817792

O	-5.8290184976	3.6568325568	0.7413295362
C	-4.2319378996	5.6813348616	-0.5402130663
C	-2.3693540191	7.0559905352	-1.2001883121
H	-1.947022761	7.9995284944	-1.4974524743
C	-4.9357402417	2.8992754118	0.4068596737
C	-3.3451138963	4.6068122516	-0.4695359089
H	-5.9499787216	1.0752818304	0.0561775895
C	-1.56737797	5.9215278614	-1.0957543783
N	-3.7503846602	3.3083823379	-0.1515270208
C	-5.0620521946	1.4088149607	0.5923286406
N	-2.0397777758	4.7244486249	-0.7371568969
N	-0.1872716776	5.9490948858	-1.3293708386
H	2.4862251388	7.0514876517	-1.2037642152
H	0.3257509964	5.1680284311	-0.9362102422
H	-5.2302928807	1.2103061187	1.6506162583
H	-3.0741786869	2.583401485	-0.3692441999
C	0.5337497783	6.88205245	-2.0319645936
C	2.0159404599	6.6086860776	-2.0839488174
H	-4.198040388	0.8479577284	0.2471111605
O	0.0448926214	7.8618555225	-2.5625819655
H	2.2507999535	5.5466244276	-2.1005939503
H	2.4284686547	7.0912018671	-2.9651360958

Dimer 1

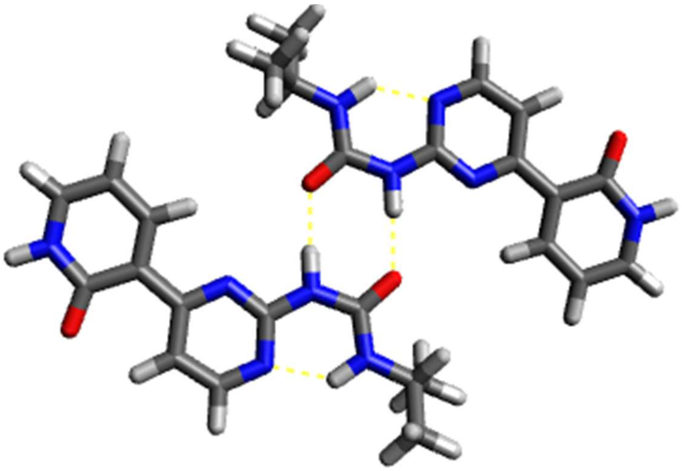

E(a.u.)= -1856.110935

N	1.8707199437	2.4349931412	-0.7272300042
C	1.7246315885	1.1050653429	-0.7031642546
C	3.1298640465	2.8664054023	-0.7192515443
C	4.2294832716	2.0328389504	-0.6876119268
C	3.9680492379	0.6577791799	-0.6634612462
N	2.7096054703	0.2114513571	-0.6706406482
C	5.0232789866	-0.3779307608	-0.6371673926
C	6.4333343357	-0.0324066174	-0.5557514397
N	7.2850417000	-1.1434244434	-0.5445557375
C	6.9129707075	-2.4374269471	-0.6020400043
C	5.5922200548	-2.7584002587	-0.6777882241
C	4.6620110641	-1.7060819146	-0.6931686036

N	0.4602398914	0.5558108273	-0.7091892845
C	-0.8066924801	1.1360225229	-0.7505621576
N	-0.8824212423	2.4763496490	-0.8027847369
C	-2.1496428615	3.1940896540	-0.8190350624
O	-1.7925249854	0.3989505196	-0.7466815441
H	3.2629093524	3.9430614994	-0.7392949256
H	5.2339585975	2.4163157090	-0.6801458888
H	7.7092140247	-3.1678098150	-0.5846956202
H	5.2801289411	-3.7895635917	-0.7254464143
H	3.6054956799	-1.9304431681	-0.7522073319
H	0.4330134168	-0.4624342964	-0.6841104032
H	-0.0073514704	2.9815557584	-0.7722854723
H	-2.8568822281	2.5596125829	-1.3534758196
C	-2.6786854132	3.4214877986	0.5912520541
C	-1.9761341697	4.4940385968	-1.5862312322
O	6.9395292525	1.0824717440	-0.4953557211
H	8.2634988515	-0.9087457021	-0.4885592174
H	-1.9964875897	4.0521929146	1.1647560027
H	-2.7942435293	2.4740985974	1.1166739040
H	-3.6513076053	3.9151914739	0.5599999098
H	-1.6368754170	4.3074162615	-2.6050424654
H	-1.2478861663	5.1449484695	-1.0967705644
H	-2.9217883476	5.0335054207	-1.6344225908
C	-5.7416829172	-3.9975539640	-0.7055312925
C	-5.4802494222	-2.6224940887	-0.7296784585
N	-4.2218057225	-2.1761658532	-0.7224989097
C	-3.2368315704	-3.0697795365	-0.6899785863
N	-3.3829194419	-4.3997075341	-0.6659162238
C	-4.6420633546	-4.8311201642	-0.6738948668
N	-1.9724399934	-2.5205247293	-0.6839531061
C	-0.7055074429	-3.1007361978	-0.6425819142
N	-0.6297782985	-4.4410634264	-0.5903626186
C	0.6374435021	-5.1588031391	-0.5741146045
C	0.4639358136	-6.4587535432	0.1930793394
C	1.1664849557	-5.3861985476	-1.9844025729
C	-6.5354794928	-1.5867844514	-0.7559693977
C	-6.1742115386	-0.2586330882	-0.6999733578
C	-7.1044207636	0.7936850624	-0.7153518507
C	-8.4251717416	0.4727113344	-0.7910924896
N	-8.7972428162	-0.8212913944	-0.8485714169
C	-7.9455352511	-1.9323090502	-0.8373770440
O	0.2803248240	-2.3636638772	-0.6464608692
O	-8.4517303671	-3.0471876632	-0.8977667067
H	-1.5048479492	-4.9462696799	-0.6208630584
H	-1.9452137073	-1.5022795777	-0.7090305869
H	-9.7757002448	-1.0559704189	-0.9045619067
H	-6.7461581232	-4.3810310035	-0.7129971659
H	-4.7751083539	-5.9077763535	-0.6538543624

H	1.3446830844	-4.5243268257	-0.0396732716
H	-0.2643124931	-7.1096626807	-0.2963818266
H	0.1246779554	-6.2721331372	1.2118912301
H	1.4095901686	-6.9982202440	0.2412688301
H	1.2820425619	-4.4388083395	-2.5098227250
H	0.4842867415	-6.0169026085	-2.5579071988
H	2.1391072249	-5.8799021871	-1.9531521480
H	-5.1176958998	-0.0342715414	-0.6409403401
H	-6.7923295652	1.8248485715	-0.6676980594
H	-9.2214152708	1.2030940199	-0.8084348554

Dimer 2

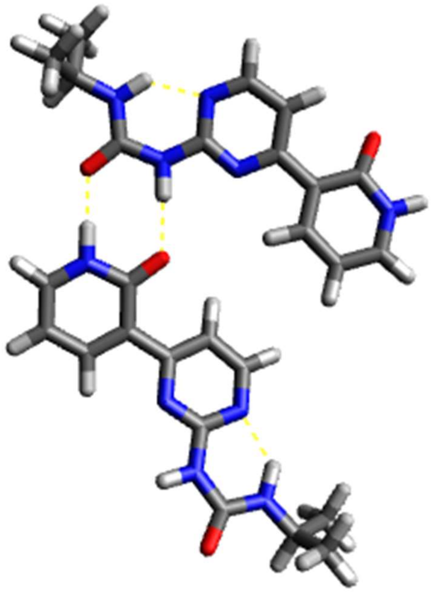

E(a.u.)= -1856.114871

N	2.1626660463	2.6505252329	-0.6668084739
C	2.0041067139	1.3234508218	-0.6493960217
C	3.4273959206	3.0683521853	-0.6727703345
C	4.5173246424	2.2218162021	-0.6628681825
C	4.2407977839	0.8498233904	-0.6375808343
N	2.9775244733	0.4179338904	-0.6343835618
C	5.2822095642	-0.1976442141	-0.5902000591
C	6.6895570460	0.1157134484	-0.7760357392
N	7.5295782285	-1.0013206862	-0.6922922160
C	7.1484507830	-2.2723235588	-0.4569185446
C	5.8297222370	-2.5625014665	-0.2818715022
C	4.9104665538	-1.5031148957	-0.3566563962
N	0.7333679674	0.7835430386	-0.6449300073
C	-0.5261669770	1.3740706494	-0.6312666683
N	-0.5944659183	2.7124749990	-0.6607426898
C	-1.8537377684	3.4449235411	-0.6080662906
O	-1.5217406462	0.6438281968	-0.6015010085

H	3.5720283721	4.1434186007	-0.6860982354
H	5.5259909713	2.5944402874	-0.6751479854
H	7.9365148502	-3.0105317109	-0.4175714344
H	5.5121232925	-3.5742618964	-0.0845879360
H	3.8554413036	-1.7026972531	-0.2237039788
H	0.7081950008	-0.2346742859	-0.6480314339
H	0.2863479022	3.2095897846	-0.6540422449
H	-2.5961380463	2.8168360665	-1.0996725815
C	-2.2969678035	3.6813093805	0.8296833989
C	-1.7090107363	4.7405399096	-1.3882928524
O	7.1990985124	1.2081442498	-0.9964791221
H	8.5066001094	-0.7909551255	-0.8225989698
H	-1.5751443770	4.3050000959	1.3606112393
H	-2.3935405126	2.7368094307	1.3641708847
H	-3.2637161754	4.1861311366	0.8530326240
H	-1.4290648910	4.5473332220	-2.4237009638
H	-0.9480791535	5.3850935473	-0.9422053006
H	-2.6504415436	5.2892262522	-1.3854937947
C	1.9097803292	-4.6439726070	-1.2421341248
C	0.6626767773	-5.1308707206	-0.8286934848
N	0.5030752452	-6.4316476232	-0.5649007312
C	1.5572389178	-7.2315048680	-0.7100815166
N	2.7809667440	-6.8680376667	-1.1016422743
C	2.9245200088	-5.5696562429	-1.3639725935
N	1.3080670472	-8.5550644767	-0.4114304412
C	2.1004539929	-9.7139452146	-0.4391345826
N	3.3742917478	-9.5706940420	-0.8434349872
C	4.3146659738	-10.6830390132	-0.8804602658
C	5.3099725366	-10.4593102462	-2.0063204768
C	4.9986933685	-10.8774409441	0.4663764832
C	-0.5365408688	-4.2816863584	-0.6914668025
C	-1.7775358688	-4.8741478848	-0.5869350271
C	-2.9656664584	-4.1392481976	-0.4770545657
C	-2.8690776132	-2.7777692631	-0.4729361970
N	-1.6695751485	-2.1805698005	-0.5644649106
C	-0.4477172988	-2.8344360347	-0.6712782636
O	1.5848075497	-10.7724272286	-0.1071834406
O	0.5795721669	-2.1530763686	-0.7280705049
H	3.6832798082	-8.6326231854	-1.0573019092
H	0.3659151437	-8.7422651927	-0.1158636778
H	-1.6134802615	-1.1556915357	-0.5644043941
H	2.0641287151	-3.6010045408	-1.4547456993
H	3.9108171919	-5.2579743220	-1.6924657377
H	3.7264856614	-11.5737840445	-1.0998230140
H	5.8981653047	-9.5542190591	-1.8369539727
H	4.8030818053	-10.3627027935	-2.9663187580
H	6.0035088204	-11.2974724896	-2.0690195372
H	4.2623725212	-11.0379027600	1.2531651711

H	5.5968860007	-10.0021436064	0.7277453301
H	5.6601372536	-11.7444753971	0.4376648872
H	-1.8182990036	-5.9546533677	-0.5975833476
H	-3.9268368180	-4.6229940379	-0.4051503668
H	-3.7231250181	-2.1188513327	-0.4003090492

Dimer 3

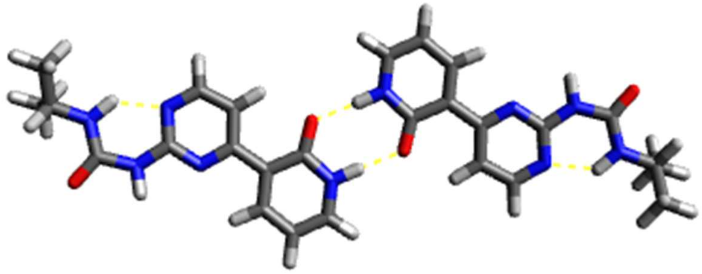

E(a.u.)= -1856.117065

N	-4.0035963089	4.9105502617	0.0960942627
C	-2.7326452943	5.3133554588	0.0127914278
C	-4.1750913759	3.5905713481	0.0915779941
C	-3.1410346769	2.6802575580	0.0074133651
C	-1.8462885189	3.2065046895	-0.0741494716
N	-1.6601171247	4.5307162368	-0.0700975985
C	-0.6253376029	2.3773273063	-0.1627374393
C	-0.6754561998	0.9313715092	-0.2294014246
N	0.5629574557	0.3096771808	-0.3093122821
C	1.7526939910	0.9338437835	-0.3267358130
C	1.8165565100	2.2950054447	-0.2640251587
C	0.6078969548	2.9972476358	-0.1830660615
N	-2.4509237962	6.6632754761	0.0098393423
C	-3.2522894533	7.8151314590	0.0763678754
N	-4.5817750527	7.6292171140	0.1387251838
C	-5.5241336468	8.7341802387	0.2598744936
O	-2.6951925110	8.9040106010	0.0627725140
H	-5.2011808368	3.2449772988	0.1593781398
H	-3.3187699113	1.6203576417	0.0041430582
H	2.6225879722	0.2954183031	-0.3918517845
H	2.7676870282	2.8030401339	-0.2770077765
H	0.6214890314	4.0769537442	-0.1332625542
H	-1.4717844296	6.8821204553	-0.0481399180
H	-4.9085712929	6.6738183654	0.1788643608
H	-5.0753365368	9.5785929683	-0.2626784751
C	-6.8241342921	8.3602085573	-0.4318955521
C	-5.7352422482	9.1263309145	1.7163674675
O	-1.6884887444	0.2190135667	-0.2241572662
H	0.5375873348	-0.7145542710	-0.3587241175
H	-7.2875194095	7.4927732239	0.0441273927
H	-6.6568197587	8.1243566707	-1.4827221914

H	-7.5326677743	9.1861905245	-0.3757405055
H	-4.7884231180	9.3930606503	2.1848610141
H	-6.1786054442	8.3028679729	2.2799380528
H	-6.4037788048	9.9853899207	1.7879582659
C	1.9624882390	-5.0000125488	-0.5936271153
C	0.6632311439	-5.5186837617	-0.6471745546
N	0.4728054138	-6.8357503721	-0.7786508508
C	1.5455461081	-7.6187465460	-0.8551741525
N	2.8207163486	-7.2230273576	-0.8121038466
C	2.9964516735	-5.9101697388	-0.6805848290
N	1.2592208460	-8.9609347691	-0.9901126142
C	2.0589366279	-10.1100198906	-1.1060574944
N	3.3903101956	-9.9272763240	-1.1118668143
C	4.3345125011	-11.0353359850	-1.1777923976
C	5.5870254018	-10.5816103505	-1.9083721484
C	4.6405333603	-11.5868884255	0.2085199459
C	-0.5578082208	-4.6893577661	-0.5612363614
C	-1.7919680217	-5.3077241776	-0.5686199046
C	-3.0007093138	-4.6053446507	-0.4901095114
C	-2.9360834193	-3.2454649946	-0.4045353608
N	-1.7455024137	-2.6227166608	-0.3966658822
C	-0.5068424640	-3.2448246898	-0.4691993215
O	1.4981127942	-11.1926398944	-1.2040889988
O	0.5069458015	-2.5337359542	-0.4518085534
H	3.7226118586	-8.9811236604	-0.9871092726
H	0.2775840942	-9.1755854959	-1.0069344025
H	-1.7196553892	-1.5992860954	-0.3329708599
H	2.1435630691	-3.9457213215	-0.4903301917
H	4.0260598491	-5.5704852146	-0.6429933759
H	3.8488186672	-11.8177820063	-1.7603668851
H	6.0842668882	-9.7710355556	-1.3703321169
H	5.3510916349	-10.2305148945	-2.9128243028
H	6.2946402903	-11.4060496274	-1.9920402033
H	3.7259720073	-11.9077939291	0.7062092136
H	5.1234545154	-10.8298616402	0.8297860296
H	5.3091431256	-12.4460704319	0.1391224692
H	-1.8062439259	-6.3862818711	-0.6389044448
H	-3.9524857304	-5.1122945722	-0.4964724810
H	-3.8059956711	-2.6070087686	-0.3399672299

Geometry of transition states

1a-TS-1a'

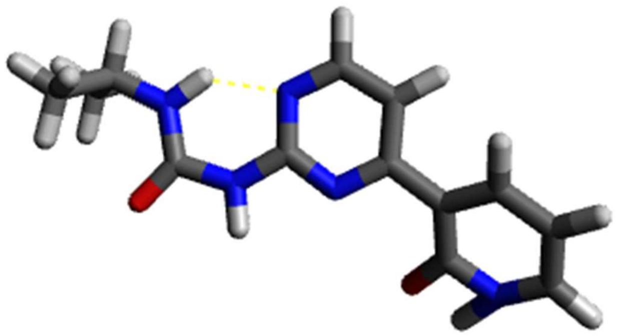

E(a.u.)= -927.9849649

N	-2.5364209239	1.4835886697	-0.1643780418
C	-1.5836651266	0.5622935747	-0.0021515162
C	-2.1044258235	2.7363434999	-0.3011140783
C	-0.7714027681	3.0849370221	-0.2776130239
C	0.1441489105	2.0419855142	-0.0921933154
N	-0.2662711110	0.7846715318	0.0438821949
C	1.5928033998	2.2965254515	-0.0277358831
C	2.5549209362	1.3695892615	-0.4936315864
N	3.8798262157	1.6566663298	-0.3982814760
C	4.3613399394	2.7833511677	0.1167539444
C	3.4785930119	3.7358897242	0.5883686619
C	2.1103694895	3.4741486539	0.5015704130
N	-1.9341004123	-0.7595120109	0.1404230078
C	-3.1689289301	-1.4386202925	0.1606002294
N	-4.2700408561	-0.6812122078	0.0162947712
C	-5.6430064171	-1.1909202043	0.0060199802
O	-3.1481707603	-2.6510950469	0.3078830821
H	-2.8695017607	3.4917194575	-0.4434776304
H	-0.4630280539	4.1086671651	-0.4212804363
H	5.4354401129	2.9054940994	0.1450854231
H	3.8440974910	4.6552024237	1.0193353713
H	1.4208724171	4.2158964458	0.8840801209
H	-1.1528438287	-1.3838360351	0.2470592275
H	-4.1125393467	0.3083679362	-0.1032084010
H	-6.2507490866	-0.2938858266	-0.1245431817
C	-5.9101399069	-2.1070460551	-1.1810439159
C	-6.0344776404	-1.8242923384	1.3344864833
O	2.4990894918	0.2291492885	-1.0737614194
H	3.8214462467	0.5280744446	-0.9713252742
H	-5.3330432636	-3.0267737759	-1.1029129566
H	-5.6495987695	-1.6137489364	-2.1176345497
H	-6.9684458580	-2.3677662273	-1.2180983074
H	-5.8582225590	-1.1342610104	2.1597898723
H	-5.4650580491	-2.7337839307	1.5170138166
H	-7.0948664930	-2.0788077316	1.3257383935

1a'-TS-1a"

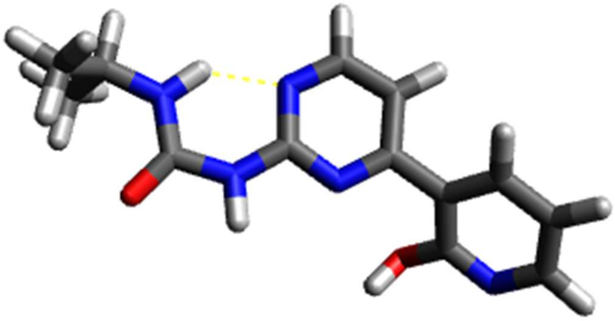

E(a.u.)= -928.0248062

N	0.9841453403	0.0128427132	-1.2032271145
C	0.5408421079	0.2212136437	0.0388780714
C	0.0471426952	-0.2068108852	-2.1224393866
C	-1.3025432341	-0.2257924536	-1.8359029902
C	-1.6570534424	0.0106489019	-0.5064023875
N	-0.7388625763	0.2340025779	0.4296045884
C	-3.0726289430	-0.0118345904	-0.0781054288
C	-3.6362384274	1.0081588710	0.7053883390
N	-4.8763805290	0.9788231635	1.1550142421
C	-5.6405003438	-0.0673741715	0.8398395277
C	-5.2066511610	-1.1113071516	0.0402972450
C	-3.9005672573	-1.0740587871	-0.4220747442
N	1.4424837390	0.4455737436	1.0514231910
C	2.8511074716	0.4971314520	1.1027228489
N	3.4996824691	0.3111122796	-0.0590023837
C	4.9566280003	0.3235269376	-0.2127635978
O	3.3712768317	0.7053803862	2.1880539943
H	0.4006717131	-0.3708308077	-3.1346386928
H	-2.0397870062	-0.3938829369	-2.6056686937
H	-6.6473342034	-0.0631109086	1.2428725837
H	-5.8656428373	-1.9324778618	-0.2024373004
H	-3.5125372844	-1.8818374205	-1.0294156009
H	1.0246932543	0.5898111092	1.9544357983
H	2.9202608620	0.1485423213	-0.8690801333
H	5.1064608499	0.1435799749	-1.2786892290
C	5.5583761657	1.6830936658	0.1167379635
C	5.6262857970	-0.8140669798	0.5471582918
O	-2.9194986251	2.1401425792	0.9474921202
H	-2.4865291881	2.0947695985	1.8022486098
H	5.4340870066	1.9192062213	1.1721300180
H	5.0853618983	2.4693833774	-0.4719894715
H	6.6244753151	1.6825783610	-0.1127023974
H	5.1952179630	-1.7749666539	0.2655380342
H	5.5142981062	-0.6870689569	1.6227790291

H	6.6912574727	-0.8391013135	0.3139250560

1b-TS-1a

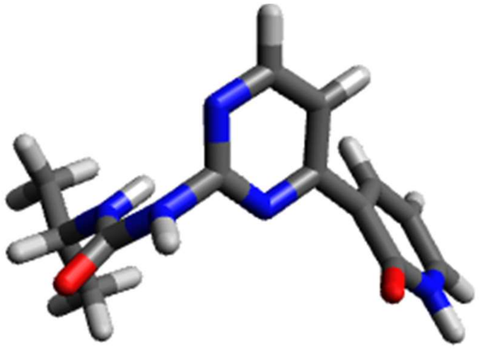

E(a.u.)= -928.0242502

N	0.6877274404	3.3093818147	-0.2848698111
C	1.0025051619	2.0454437681	-0.0151361854
C	-0.6096724833	3.5531168964	-0.4643095487
C	-1.5766460887	2.5687144956	-0.3762195291
C	-1.1385927826	1.2793429283	-0.0822335612
N	0.1625895542	1.0253081527	0.1016239238
C	-2.0838974885	0.1545379366	0.0635973537
C	-1.8505087185	-1.0654389174	-0.6944455837
N	-2.7788290152	-2.0734326085	-0.4189825252
C	-3.8332937194	-1.9756445616	0.4213200044
C	-4.0676493565	-0.8172793601	1.0921775478
C	-3.1693737335	0.2531730129	0.8917905183
N	2.3803332000	1.7667731687	0.1604479620
C	3.0179893356	1.5124421693	1.3611858380
N	2.3191108499	1.8280746126	2.4844583856
C	2.8259050425	1.5520989299	3.8228773503
O	4.1619135400	1.0799538830	1.3776844386
H	-0.8822423402	4.5777903579	-0.6925792399
H	-2.6195021008	2.7911370761	-0.5462881043
H	-4.4531015148	-2.8554667780	0.5156112572
H	-4.9062004238	-0.7329157254	1.7645695120
H	-3.3332468883	1.1764360933	1.4347701527
H	2.8685777507	1.3896451886	-0.6333011219
H	1.3436304697	2.0513057218	2.4024710410
H	3.9088311932	1.6401965716	3.7491330174
C	2.3097199096	2.6076780909	4.7863603396
C	2.4845990812	0.1379355317	4.2732624621
O	-0.9793396471	-1.2581535875	-1.5283356217
H	-2.6274904297	-2.9300313730	-0.9281438539
H	1.2194717293	2.5789290856	4.8560905655
H	2.6051158817	3.6071042793	4.4681766137
H	2.7076362903	2.4347434396	5.7859764595

H	2.8750031786	-0.5949392586	3.5680870628
H	1.4031712217	0.0024328724	4.3460040515
H	2.9177559005	-0.0673939073	5.2531688283

1a''-TS-1d''

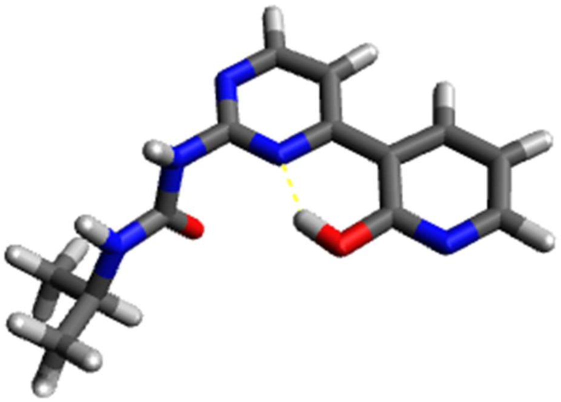

E(a.u.)= -928.0250653

N	-0.8308925571	1.5965112525	0.6170309667
C	-0.4852028447	0.4580042101	0.0291523661
C	0.1871383860	2.3737929148	0.9780341944
C	1.5155684527	2.0437423570	0.7992088463
C	1.7868063129	0.8098447738	0.2019873408
N	0.7658799732	0.0329886364	-0.1886107546
C	3.1545685406	0.3124011827	-0.0332517481
C	3.4251308348	-0.9248143881	-0.6830146035
N	4.6527622099	-1.3789336457	-0.9002496984
C	5.6822482206	-0.6469220730	-0.4926020754
C	5.5510887374	0.5742579331	0.1514181145
C	4.2700372626	1.0426982112	0.3750144519
N	-1.4670685553	-0.4260523036	-0.3585679409
C	-2.7982742605	-0.1016910739	-0.7354722035
N	-3.5807646977	-1.2123175358	-0.7236344005
C	-4.9411477802	-1.4266235157	-1.2386052508
O	-3.1355306985	1.0202897357	-1.0322193352
H	-0.0779695386	3.3138992500	1.4502144442
H	2.2874261332	2.7229247042	1.1209715385
H	6.6667001199	-1.0584736705	-0.6904602965
H	6.4198009943	1.1341437851	0.4653880149
H	4.1413322442	1.9908712677	0.8767355805
H	-1.1180497573	-1.3224730916	-0.6483553632
H	-3.1613247489	-2.0376790631	-0.3346169658
H	-4.8422900173	-2.1231827245	-2.0773357295
C	-5.7694287407	-2.1083140277	-0.1583550506
C	-5.6262531227	-0.1785163519	-1.7623299676
O	2.4558223622	-1.7199223379	-1.1245393810
H	1.5982928795	-1.2882291056	-0.9019175438

H	-5.9175652000	-1.4411006446	0.6918580989
H	-5.2873566953	-3.0188580780	0.2012391301
H	-6.7466006389	-2.3852029539	-0.5541620697
H	-5.0544674564	0.3022609140	-2.5512705609
H	-5.7916780320	0.5523857001	-0.9728731693
H	-6.5947383215	-0.4727102428	-2.1678089789

1d”-TS-1e”

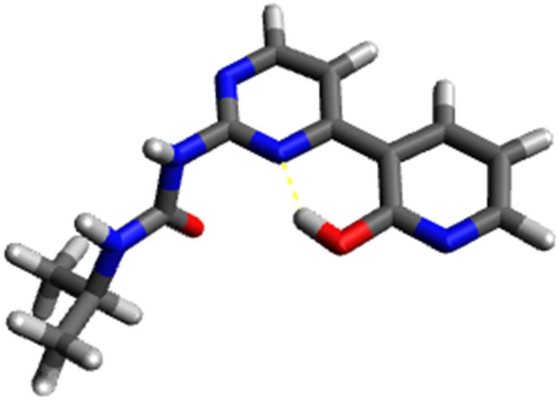

E(a.u.)= -928.0067655

N	-0.5412661092	2.3722504622	0.1167684069
C	-0.5050021225	1.0522465074	0.1503387495
C	0.6510500001	2.9728987964	0.0391122347
C	1.8444080131	2.2876895951	-0.0139713339
C	1.7872199335	0.8903834396	0.0321625567
N	0.5860036028	0.2962560240	0.1208300793
C	2.9775780846	0.0271906514	-0.0011349104
C	2.8996897196	-1.3943816788	0.0578383109
N	3.9705390966	-2.1788760260	0.0402646367
C	5.1698112725	-1.6174638472	-0.0435275272
C	5.3766361117	-0.2474142110	-0.1153633588
C	4.2607425314	0.5665545274	-0.0912294599
N	-1.7497619153	0.3829052492	0.2425634161
C	-2.1903608781	-0.4000498360	-0.8179936718
N	-3.2267966632	-1.2287619444	-0.5094721080
C	-3.9733909994	-1.9494976058	-1.5367138696
O	-1.6843511570	-0.3237407961	-1.9243647142
H	0.6414081949	4.0567217919	0.0152282395
H	2.7716728875	2.8326905545	-0.0833358622
H	6.0141213094	-2.2990493252	-0.0550998041
H	6.3733893513	0.1624582965	-0.1862819097
H	4.3953921980	1.6374277943	-0.1448009427
H	-2.0269651644	0.1227578547	1.1738574522
H	-3.6940445369	-1.0934096441	0.3703088737
H	-3.2423238706	-2.2143383093	-2.2990986295
C	-4.5469559744	-3.2228860227	-0.9392123414

C	-5.0447575927	-1.0737009854	-2.1723533227
O	1.7439174530	-2.0417136269	0.1369690606
H	1.0224394862	-1.3707767428	0.1361297794
H	-5.2610331285	-2.9970442130	-0.1433069842
H	-3.7595814314	-3.8518424915	-0.5247365888
H	-5.0753182302	-3.7936757873	-1.7022595236
H	-4.6049791169	-0.1740843858	-2.6015245327
H	-5.7914246922	-0.7738890011	-1.4335375547
H	-5.5577056630	-1.6138350643	-2.9690528460

1f-TS-1c

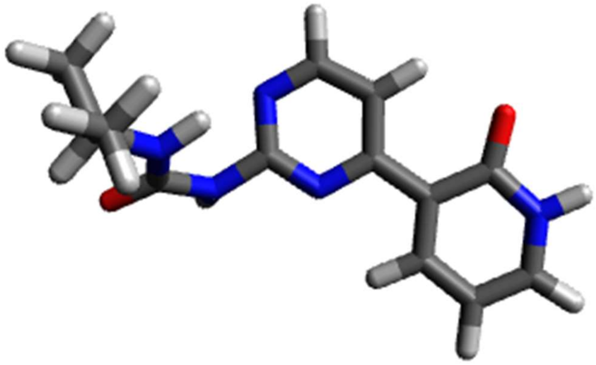

E(a.u.)= -928.0443692

N	-0.8778353468	-0.5079103744	-0.7411270411
C	-0.3050558418	0.6670059984	-0.4812770556
C	-0.0453537525	-1.5484712681	-0.7574834277
C	1.3068644218	-1.4474160647	-0.4899267161
C	1.8023239123	-0.1724347732	-0.2055527268
N	0.9708406965	0.8834643515	-0.2201009192
C	3.2134129010	0.1099109250	0.1178793325
C	4.2166328274	-0.9428709989	0.1338938579
N	5.4989671269	-0.4933279993	0.4680734214
C	5.8508681260	0.7746575765	0.7569835892
C	4.9107188104	1.7596909601	0.7391082487
C	3.5928893703	1.4008532377	0.4149178465
N	-1.1450506164	1.8093757483	-0.4022326423
C	-2.0990284187	2.1737579984	-1.3522226424
N	-1.9231809339	1.6791279635	-2.6048590720
C	-2.8889395261	1.9220000300	-3.6713053333
O	-2.9791694748	2.9676586453	-1.0522646833
H	-0.4868951972	-2.5136722015	-0.9809121491
H	1.9516022610	-2.3085601549	-0.4953019209
H	6.8915199899	0.9426880806	0.9942509817
H	5.1791173114	2.7784787150	0.9682674369
H	2.8252370686	2.1618498297	0.3937755823
H	-1.5015193985	1.9966013467	0.5214459989

H	-1.3473583709	0.8614815768	-2.7044529356
H	-3.2895417745	2.9168758693	-3.4841947990
C	-2.1707512061	1.9226969437	-5.0105516686
C	-4.0360464464	0.9207329407	-3.6297055034
O	4.0656685454	-2.1352008160	-0.1060193813
H	6.1979820836	-1.2193624585	0.4807157061
H	-1.7189875371	0.9491589002	-5.2166293856
H	-1.3833500216	2.6756256105	-5.0324038548
H	-2.8725863063	2.1369409486	-5.8163592082
H	-4.5394227565	0.9491044614	-2.6640074482
H	-3.6721751621	-0.0953119275	-3.7977329032
H	-4.7703973642	1.1478003795	-4.4036885846

1c-TS-1b

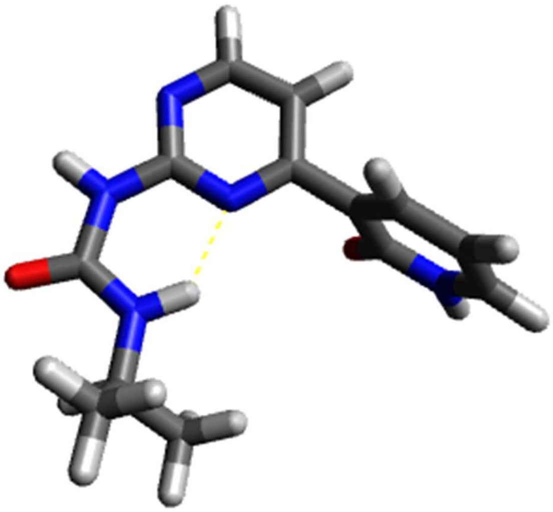

E(a.u.)= -928.0414466

N	0.4192500570	3.2203718242	-0.1144727240
C	0.8492814870	1.9536904209	-0.0386874878
C	-0.8925817840	3.3754934789	-0.2092058205
C	-1.7858633849	2.3134774415	-0.2336750580
C	-1.2327968987	1.0450058798	-0.1543510036
N	0.0857569644	0.8601431040	-0.0571482787
C	-2.0794253363	-0.1743519029	-0.1848180035
C	-2.5543344953	-0.6961377948	1.0812882383
N	-3.3280961668	-1.8480417046	0.9521525344
C	-3.6420585771	-2.4642094073	-0.2129176479
C	-3.1947294327	-1.9636676933	-1.3928700391
C	-2.3980311388	-0.7945883480	-1.3574417666
N	2.2144936517	1.8247396107	0.0579647397
C	3.0719778609	0.7174104620	0.1917875830
N	2.4951490863	-0.4946797845	0.2360197447
C	3.2640476204	-1.7275980241	0.3498539802
O	4.2736107919	0.9288127315	0.2656102903

H	-1.2514507609	4.3976714284	-0.2696642319
H	-2.8513859315	2.4676495332	-0.3067353290
H	-4.2530560927	-3.3515059419	-0.1337584108
H	-3.4421016766	-2.4496928913	-2.3230860290
H	-2.0284534159	-0.3831078859	-2.2892967207
H	2.7136428172	2.6973261981	0.0571425071
H	1.4898397678	-0.5282638582	0.1426032099
H	4.1487789509	-1.4864411403	0.9384177217
C	2.4392654995	-2.7598834709	1.0999038548
C	3.7163840226	-2.2277183013	-1.0158698669
O	-2.3356778388	-0.2205415717	2.1888416943
H	-3.6656929434	-2.2296731169	1.8218191731
H	1.5231790636	-3.0016852336	0.5557455109
H	2.1625400180	-2.3981173291	2.0898653578
H	3.0073303171	-3.6821274040	1.2187195232
H	4.3085537470	-1.4699462784	-1.5275578236
H	2.8573953942	-2.4748850788	-1.6425976419
H	4.3292587570	-3.1239279515	-0.9105817800

1c"-TS-1e"

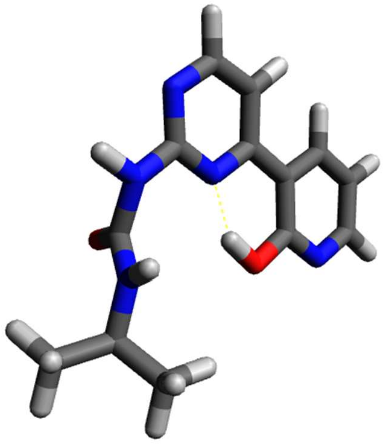

1. DFT calculations (additional discussion)

2 Additional discussion

Before additional discussion we would like to remind the OH/NH forms of 2-hydroxypyridine are known to coexist in complexes where OH and NH forms are in proximity and are forced geometrically to interact.[1],[2]

2.1 The stability of tautomeric/rotameric forms

To have a further insight into the experimental data a set of calculations was performed. First, among 20 forms optimized the most stable is **1f**. In this form the

intramolecular hydrogen bond between H15 and N1 stabilize it. The pyridone ring is rotated in such a way as to avoid close proximity of N3 and O20 (electronic repulsion). On the other hand, forms **1c** and **1b** are only 2.3 and 7.5 kJ/mol higher in energy, respectively. It is worth mentioning that in **1b** two intramolecular hydrogen bonds are present. Taking into account next forms that are a bit higher in energy one sees **1a** and **1a''**. Most probably these forms coexist. The detailed numerical data related to relative energies are collected in Table S1 while the structures are shown in the main text.

Table S1. The relative energy of monomeric forms of **1**[a]

Form	Relative energy [kJ/mol]
**1a**	14,4
**1a'**	30,9
**1a''**	15,2
**1b**	7,5
**1b'**	27,3
**1c**	2,3
**1c'**	24
**1c''**	27,9
**1d**	40,1
**1d'**	57,5
**1d''**	41,6
**1e**	46,2
**1e'**	61
**1e''**	30,4
**1e'' (PT)**[b]	59,9
**1f**	0
**1f'**	23,2
**1g**	29
**1g'**	51,9
**1h**	24,6
**1h'**	48,9

[a] – All forms were divider into three groups. More intense colour represents higher relative energy of respective form,
[b] – proton located at the N3 as in enaminone forms of 2-phenacylquinolines[3] forming N3H…O20=C hydrogen bond. As opposite to the quinoline[3] derivatives the pyrimidine ring does not tend to loose aromatic character. That is why the relative energy of **1e''(PT)** is high.

Note, that in case of **1a''** the hydroxypyridine form is stabilized by intramolecular hydrogen bond between N1 and H20. Still this form is located in the group of forms that have relatively low energy. On the opposite side of the scale the forms that are characterized by

high energy are those without intramolecular hydrogen bonding (**d** and **e** ones). Moreover, in most of those forms electronic repulsion is present.

Since the relative energy is not the only factor that should be taken into account, we studied the hydrogen bonding energy. The QTAIM[4] and Espinosa approach[5],[6] allowed us to calculate the energy of hydrogen bonds in investigated rotamers and tautomers. Thus, the strongest intramolecular hydrogen bonding was found in **1d''**, **1a''**, **1e''** and **1c''**. The values of that energy are as follows: -57.2, -55.9, -51.3 and -39.0 kJ/mol for mentioned forms, respectively. All those interactions come from N···HO bonding. This is in line with Etter's rules[7] telling that more electronegative groups form stronger interactions (OH is more acidic that NH moiety) and intramolecular hydrogen bonding is much more probable that intermolecular one.

2.2 Stability of dimers

In studied molecule only doubly hydrogen-bonded dimers are possible. This is in agreement with low dimerization constant mentioned in the main text. The most stable dimers (composed from three most stable monomeric forms of **1**) are **1f-D2**, **1c-D3** and **1f-D3** with the energy relative to **1f-D2** equal to 0.0, 5.4 and 5.6 kJ/mol. Two of those dimers are held together by two NH···O hydrogen bonds *via* pyridone moiety. The third one is stabilized by NH···O interaction between pyridone and urea moiety. So low relative energy of dimers suggests the coexistence of those forms. Similarly, as in case of monomeric species, the relative low values of energy suggest the energetic potential curve is shallow. On the other hand, the dimer with the highest relative energy is **1b-D3** where two NH···O interactions stabilize it while two strong intermolecular repulsions cause destabilization (O/O and N/N). The strongest intramolecular hydrogen bonds present in the dimeric forms of **1** was found in **1b-D3**, while the strongest intermolecular one in **1c-D2**. In fact, the highest overall stabilization (the sum of energies of hydrogen bonds in the dimer based on QTAIM) that comes from the intermolecular hydrogen bonding was noticed for **1c-D3** (-69.6 kJ/mol), **1f-D2** (-69.3 kJ/mol) and **1f-D3** (-61.3 kJ/mol). Also, for those dimers the intermolecular interactions corrected to basis set superposition error is the highest (Table S2). At this point it is worth to mention that the calculated energies may not reflect the experimental data since the studied compound has low solubility in chloroform. Thus, the low concentration causes low dimerization in moderate polar solvent.

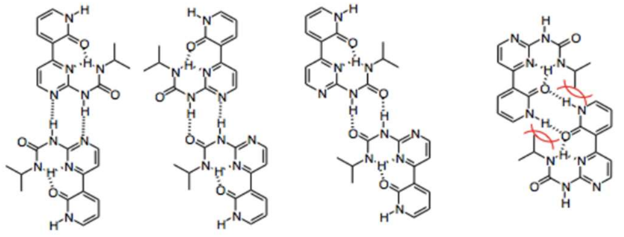

1b - D1 1b - D2 1b -D3 1b - D4

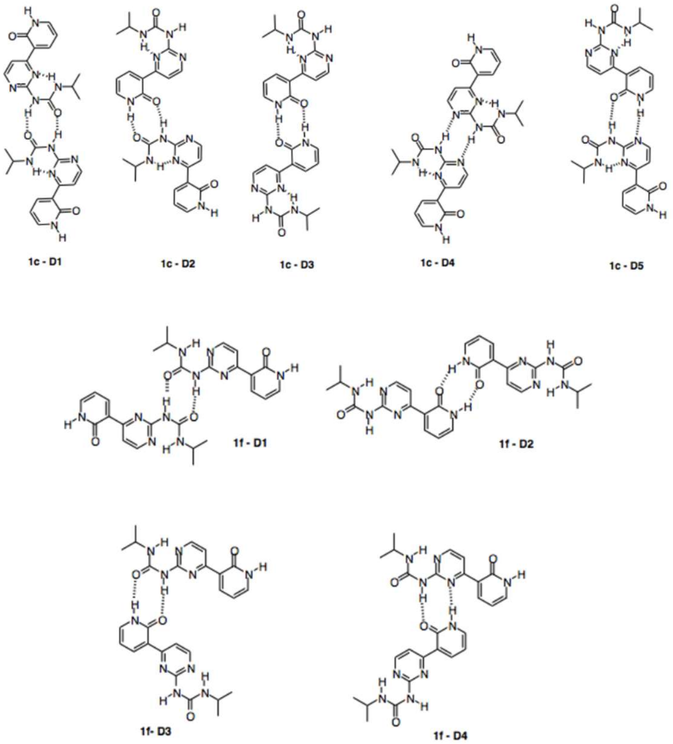

Figure S1. Investigated dimers of **1**

Table S2. The relative and interaction energy obtained for dimers of **1**

Dimer	Relative energy [kJ/mol]	Energy of interaction [kJ/mol]
**1b - D1**	29,5	-31,7
**1b - D2**	37,7	-23,6
**1b - D3**	38,9	-22,5
**1c - D1**	27,3	-23,7
**1c - D2**	14,7	-36,4
**1c - D3**	5,4	-45,7
**1c - D4**	19,5	-31,2
**1c - D5**	22,7	-28,4
**1f - D1**	17,4	-28,5
**1f - D2**	0	-46,5
**1f - D3**	5,6	-40,5
**1f - D4**	21,1	-24,7

2.3 Complexes and their properties

Complexes of 1 with A and B

The complexation of **1** was described in terms of intermolecular interactions with **A** and **B**. The lowest in energy complex of **1** and **A** is the **1h+A** (it also has a high energy of interaction that equals to -38.6 kJ/mol). On the other hand, the highest energy of intermolecular interaction was obtained for **1e''+A** (-41.4 kJ/mol) and its energy related to the most stable is only 3.0 kJ/mol. That, together with the Etter's rules (and experimenal measurements), suggest the existence of **1e''** form in complex with **A** is very probable. In fact, the experimental data confirms that - the observation of chemical shifts clearly shows the drastic change of H9/H20 and H10 positions in ^{1}H NMR spectra. The C=O17···H20(O20)···N3 bifurcation stabilizes the **1e''** form in **1e''+A**. The competitive **1h** form in **1h+A** complex is destabilized by intramolecular electronic repulsion, which is seen in twist of **1h** in the urea part (Fig. S2).

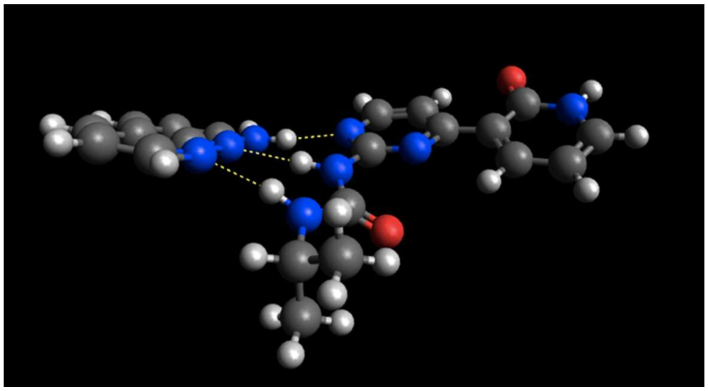

Figure S2. The structure of **1h+A** complex

While **1e''+A** is very close in energy to the most stable form of complex with **A** the path leading from **1f** (the most stable monomer) to **1e''+A** is limited by the energy of the proton transfer. The path is as follows: a) **1f** →TS1→ **1c**, b) **1c** →TS2→ **1b**, c) **1b** →TS3→ **1b'**, d) **1b'** → TS4→ **1e'** / **1b'** →TS5→ **1e''** or d) **1b'**→TS6 and TS7 → **1e''** (in the last possibility – in red in the picture below – transition states refer to rotation of OH and urea part of molecule in two steps without taking into account form **1e'**). The picture (Fig. S3) below shows the detailed path with relative energies.

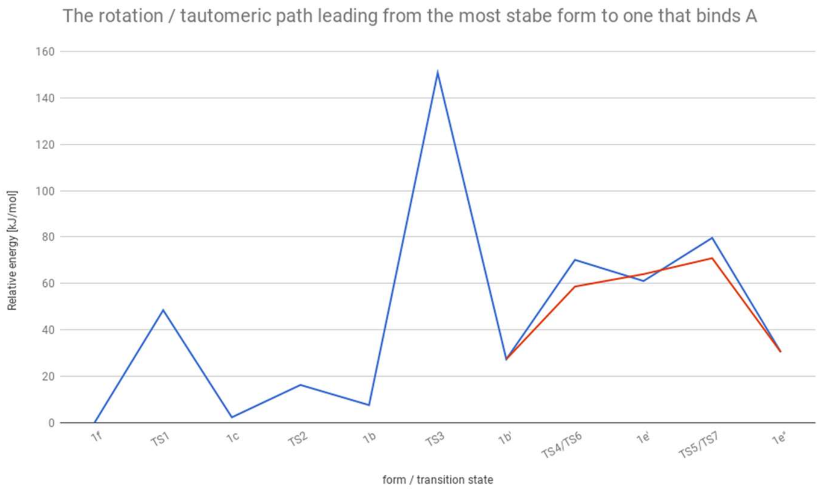

Figure S3. The energy diagram for **1f→1e’** transition

The most important is that the tautomerism in the pyridone/hydroxypyridine system (the energy of TS3) is the limiting step. In other words, any rotational equilibrium is much more probable than breaking and formation of the covalent bond, which is commonly known.

The mechanism of binding of **B** by **1** is much less complicated, *id est*. any formation of intramolecular hydrogen bond by the urea moiety allows easy complexation of **B** by triple hydrogen bonding (Fig. S4 shows the titration curves that confirm that).

The proton transfer that was observed during titration of **1** with **A** (Fig. S4) was supported by DFT calculations.

Guest exchange

The most interesting is that compound **1** remains in OH-tautomeric form (hydroxypyridine) when the counterpart **A** is replaced by **B** within the complex. This is visible in the constant chemical shift of H20 and H10 when **1+A** was titrated by **B**. It is worth to stress the chemical shifts of H13 and weak for H15 (Fig. S8). The said counterpart replacement takes place due to the rotation of the urea “arm” that, in turn, forms intramolecular H15···N3 hydrogen bond. The mentioned form **1c’’** is characterized by two intramolecular, bifurcated hydrogen bonds with N3 as a basic centre. Its relative energy (*vs* **1f**) is equal to 27.9 kJ/mol. This is the value in half-way between the lowest and the highest relative energies for monomeric forms. It is also worth to underline that two intramolecular hydrogen bonds in **1c’’** have the QTAIM-based energy of hydrogen bonding equal to -39.0 and -18.8 kJ/mol giving the sum of -57.8 kJ/mol. These two interactions are higher (but, in fact, very close to) than N···HO hydrogen bond (-57.2 kJ/mol) in **1d’’**. It is fair to mention, however, that those numbers given above referring to intramolecular hydrogen bonds are not the only data suggesting stabilization of **1c’’+B** complex. The intermolecular triple hydrogen bonding also influences stabilization of respective geometric form.

Moreover, the intramolecular hydrogen bonding is enhanced by intermolecular interactions. Thus, taking into account form **1e’’**, the only intramolecular hydrogen bond in this form (according to QTAIM) is formed between N3 and H20 (according to IUPAC recommendations regarding the presence of the hydrogen bond critical point).[8] After complexation with **A**, additionally to mentioned N3···H20 interaction (-48.4 kJ/mol, 1.773 Angstrom), the O17···H20 hydrogen bond is formed in the complex **1e’’+A** characterized by the energy of -13.4 kJ/mol (2.212 Angstrom). The last hydrogen-oxygen distance (O17···H20)

is 0.05 Angstrom shorter in **1e''+A** than that in uncomplexed **1e''**. The similar is observed for **1c''+B**. In **1c''** the intramolecular hydrogen bond has the energy of -39.0 and -18.8 kJ/mol for N···HO and N···HN interaction, respectively. The same hydrogen bonds in the complex are very close in energy, *id est.* -35.3 and -21.4 kJ/mol for N···HO and N···HN contacts. Thus, the energy of N···HN is higher (by -2.6 kJ/mol) in the complex at the expense of N···HO (+3.7 kJ/mol) one suggesting the conformation of the urea is more rigid in the complex that in the free form.

Table S3. The relative and interaction energy obtained for complexes of **1** and **A**/**B**

Complex	Relative energy [kJ/mol]	Energy of interaction [kJ/mol]
**1e+A**	19,9	-40,3
**1e'+A**	37,0	-38,1
**1e"+A**	3,0	-41,4
**1e"+A (PT)**	27,9	-46,1
**1g+A**	10,5	-32,5
**1g'+A**	34,3	-31,6
**1h+A**	0,0	-38,6
**1h'+A**	24,0	-38,9
**1b+B**	4,0	-36,8
**1b'+B**	25,1	-35,5
**1c+B**	0,0	-35,5
**1c'+B**	20,9	-36,3
**1c"+B**	28,2	-33,0

—

3 Figures for titration (and dilution) of **1**

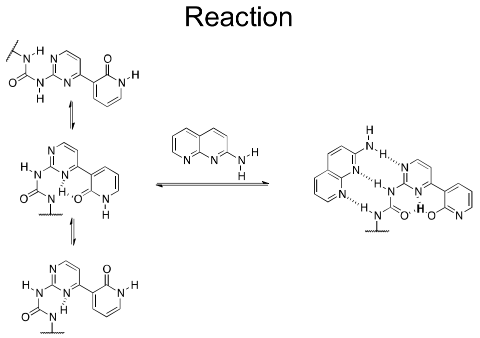

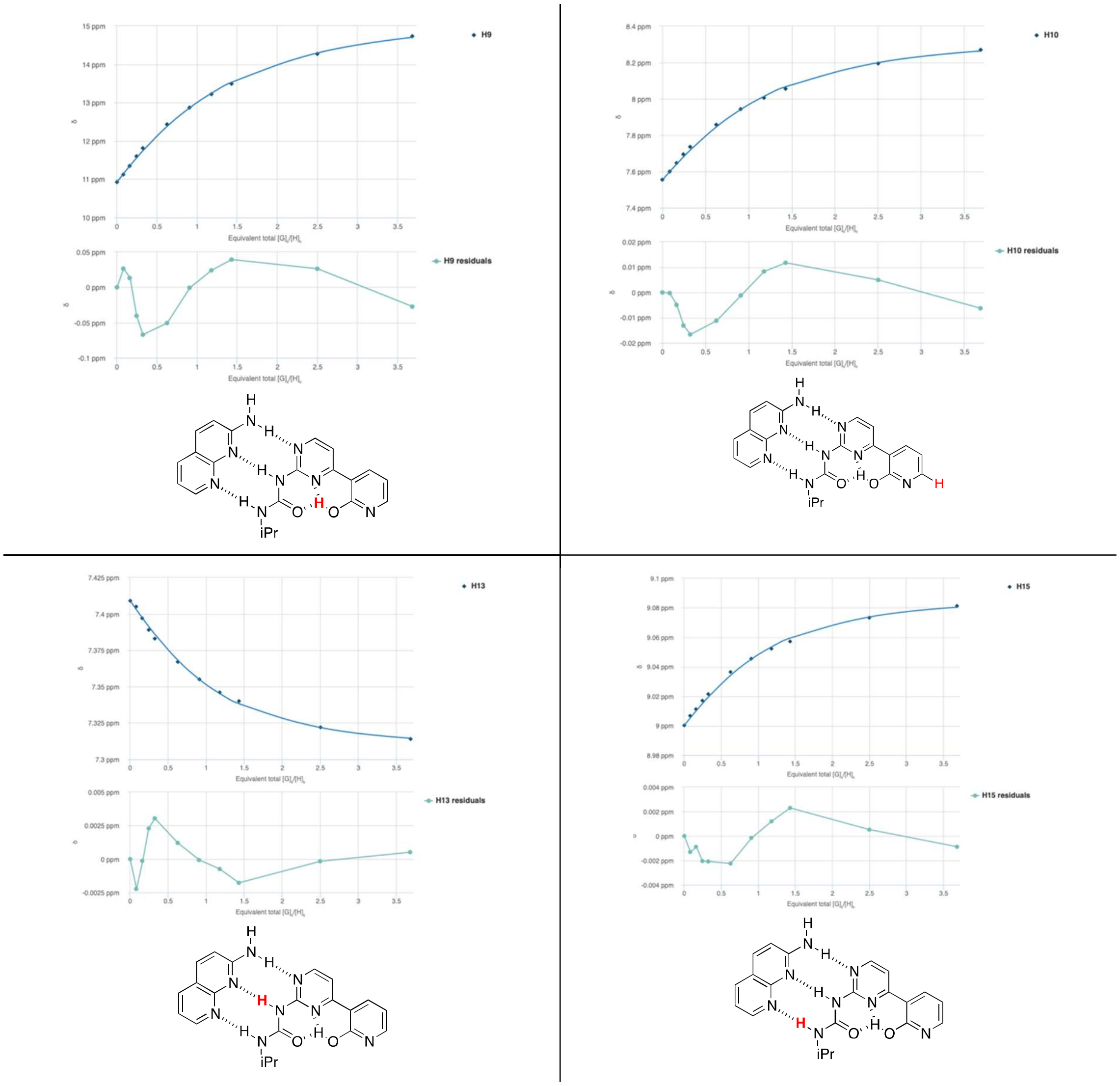

Figure S4. **1+A** 1H NMR titration

Reaction

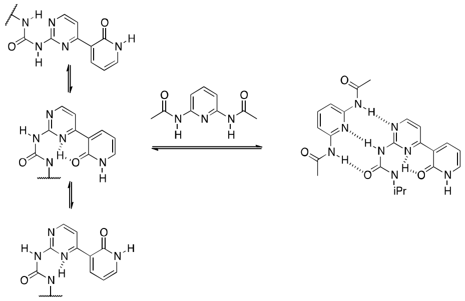

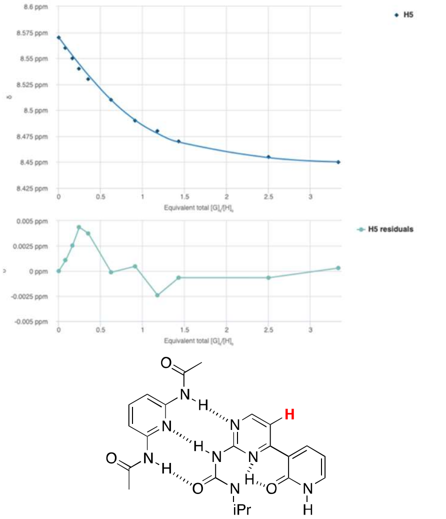

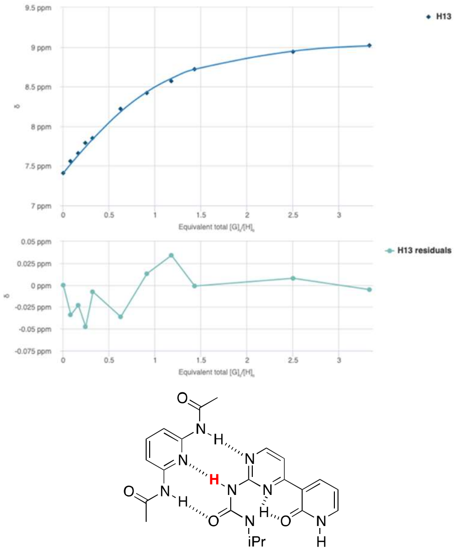

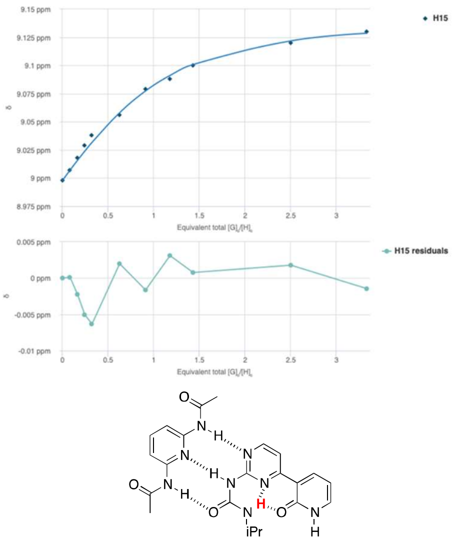

Figure S5. **1+B** 1H NMR titration

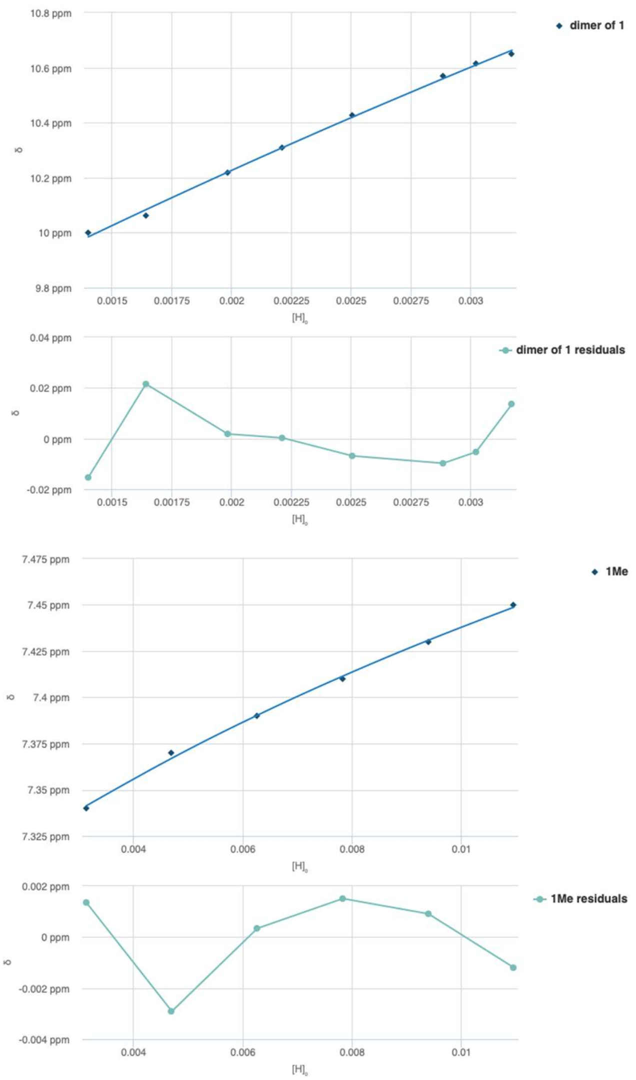

Figure S6. The dilution experiments for **1** (top) and **1Me** (bottom)

Comment: The weak curvature of the fit suggests very weak dimerization. However, it was still possible to find the dimerization constant.

Reaction

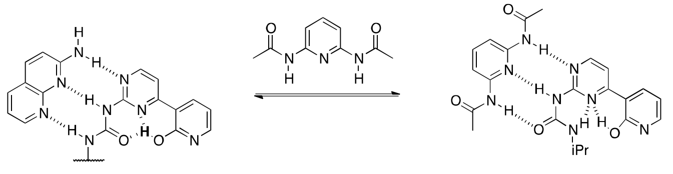

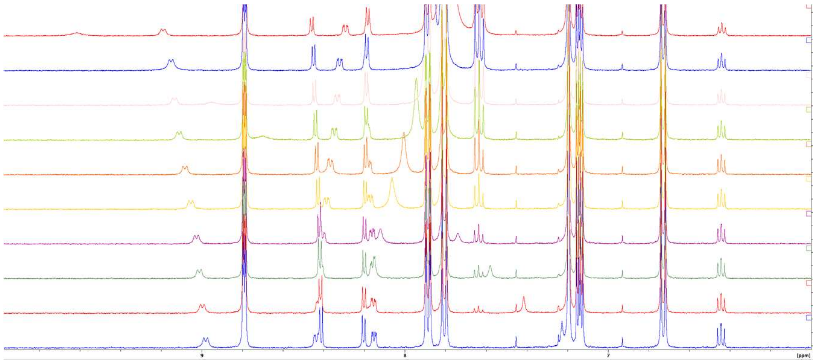

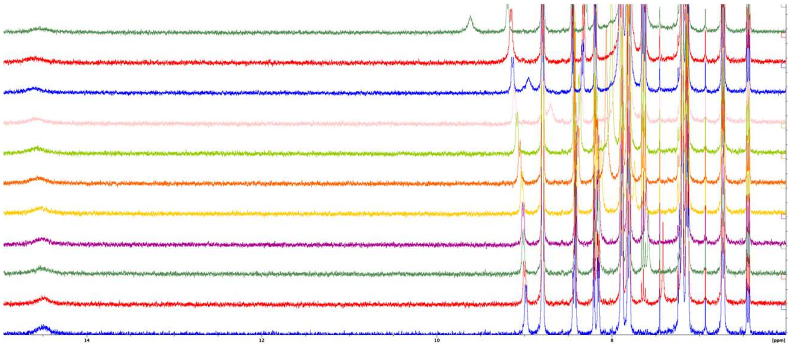

Figure S7. The stacked spectra for titration of **1+A** by **B**

Reaction

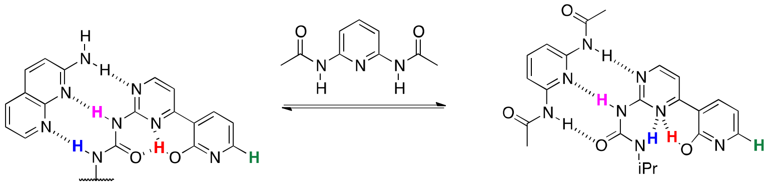

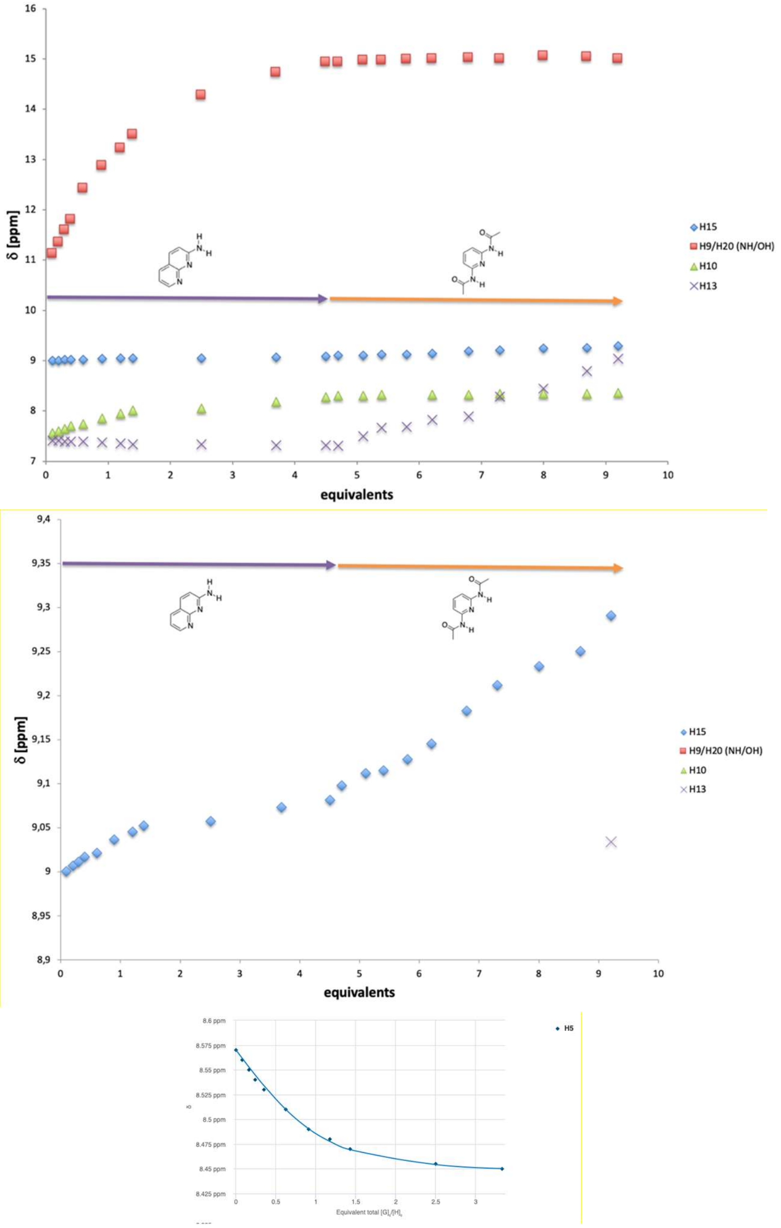

Figure S8. ^{1}H NMR titration of **1+A** with **B**
(compound **B** was added to complex **1+A** at the [**A**]:[**1**]=4.5)

*Note: The [**B**]:[**1**] ratio was equal to 4.7 at the end of experiment. In the X-axis, however, the sum of [**A**] and [**B**] was shown and the part of titration with **A** and **B** guests are marked with violet and orange arrows. The last part of the figure is a repeated titration chart (see Fig. S5) for proton H5 in **1+B** complex. The change of the course (decrease in **1+B** while increase in **1+A+B** experiment) is worth noting.*

Reaction

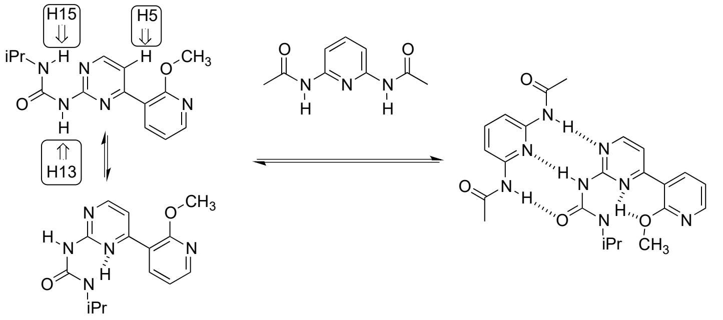

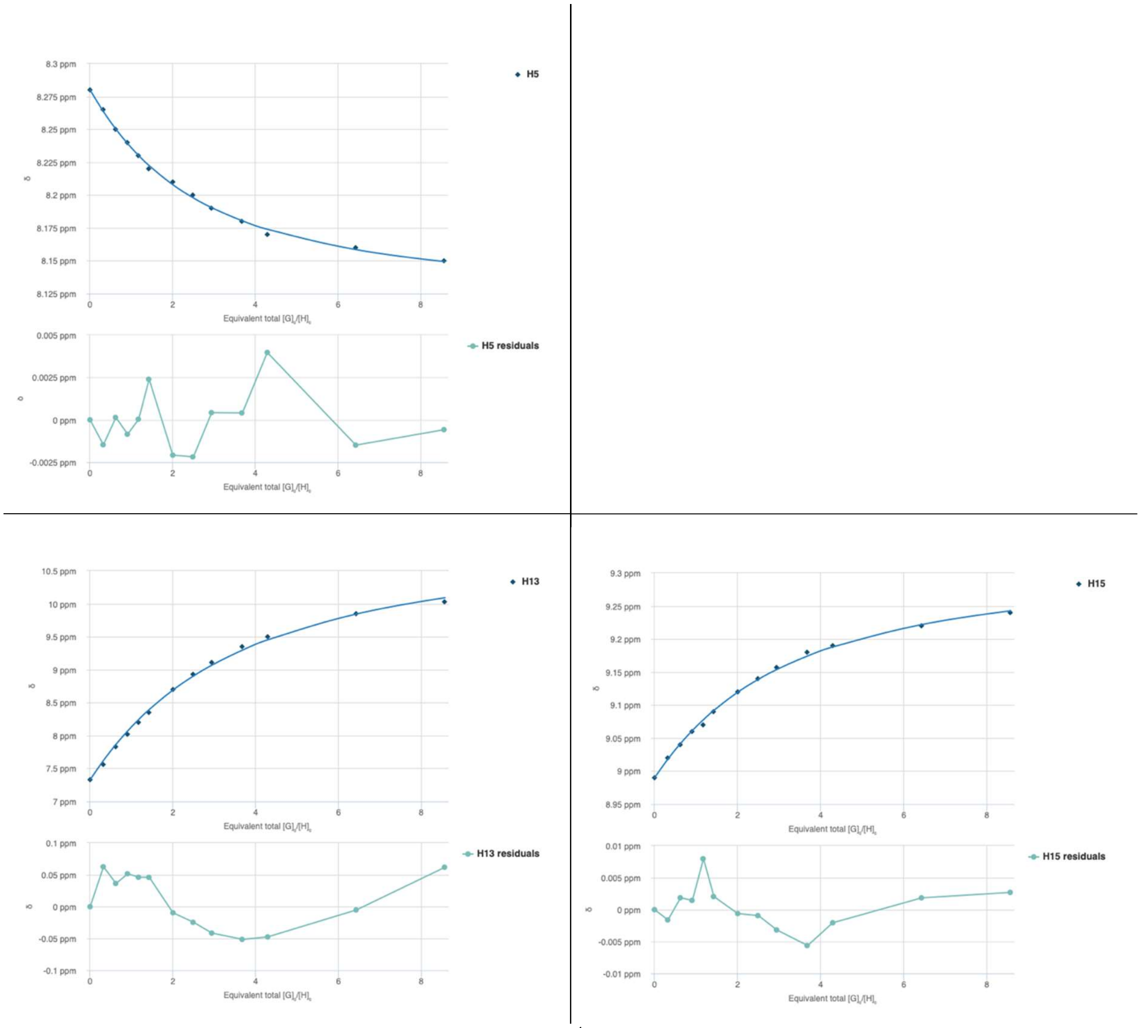

Figure S9. **1Me+B** ^{1}H NMR titration

4 NMR spectra

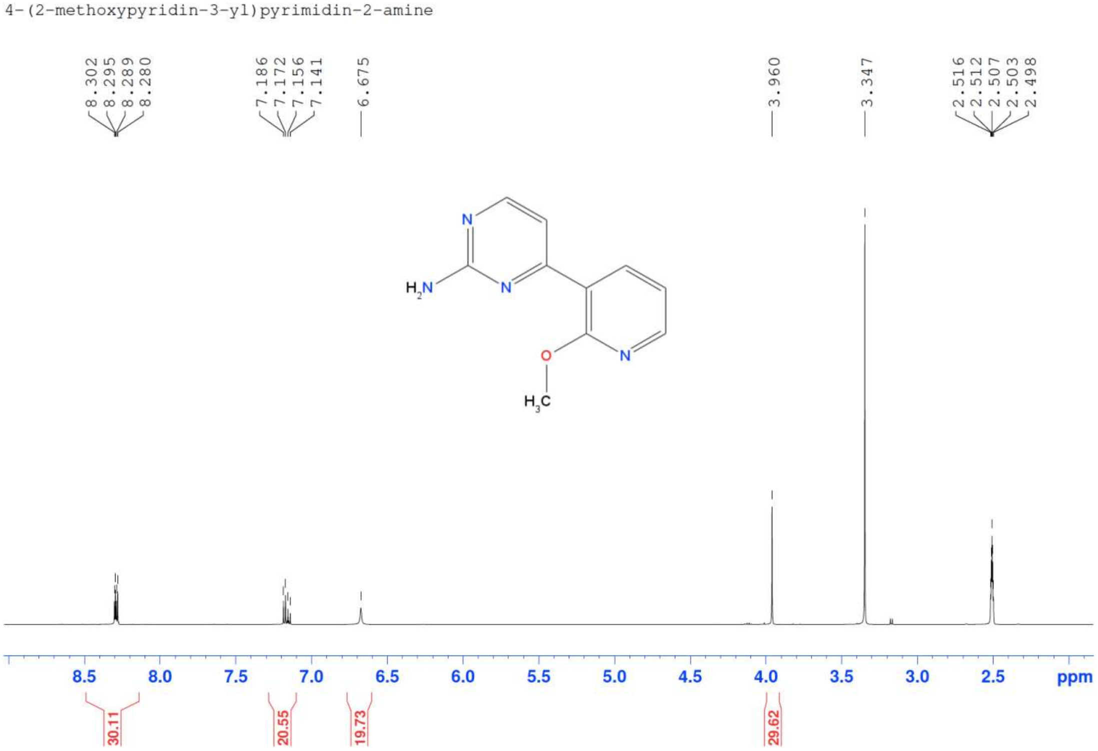

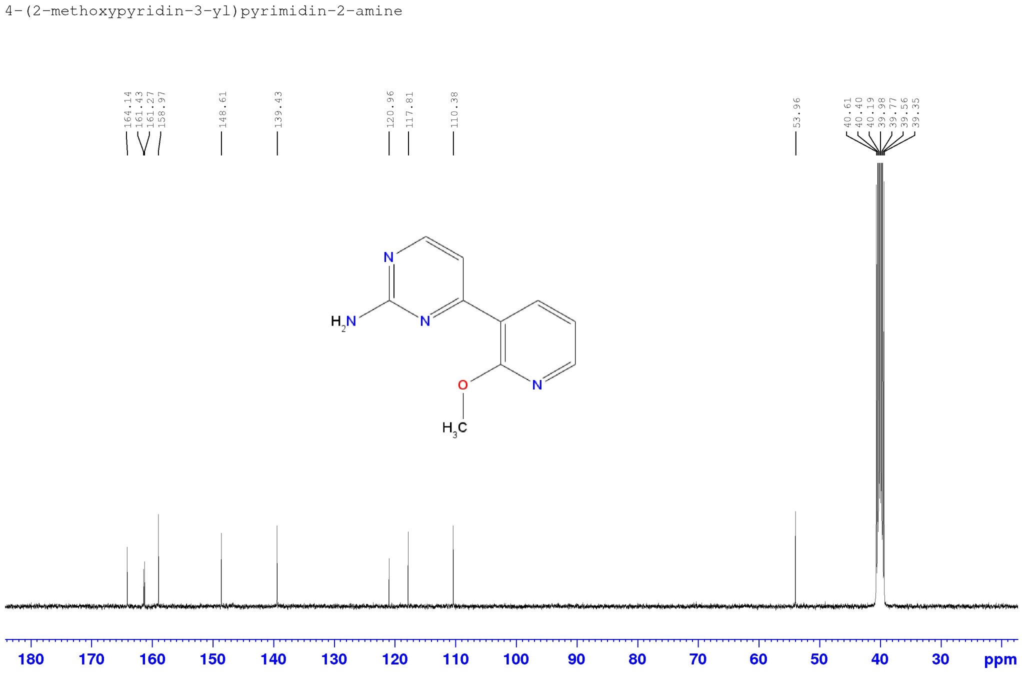

1-[4-(2-methoxypyridin-3-yl)pyrimidin-2-yl]-3-propan-2-ylurea

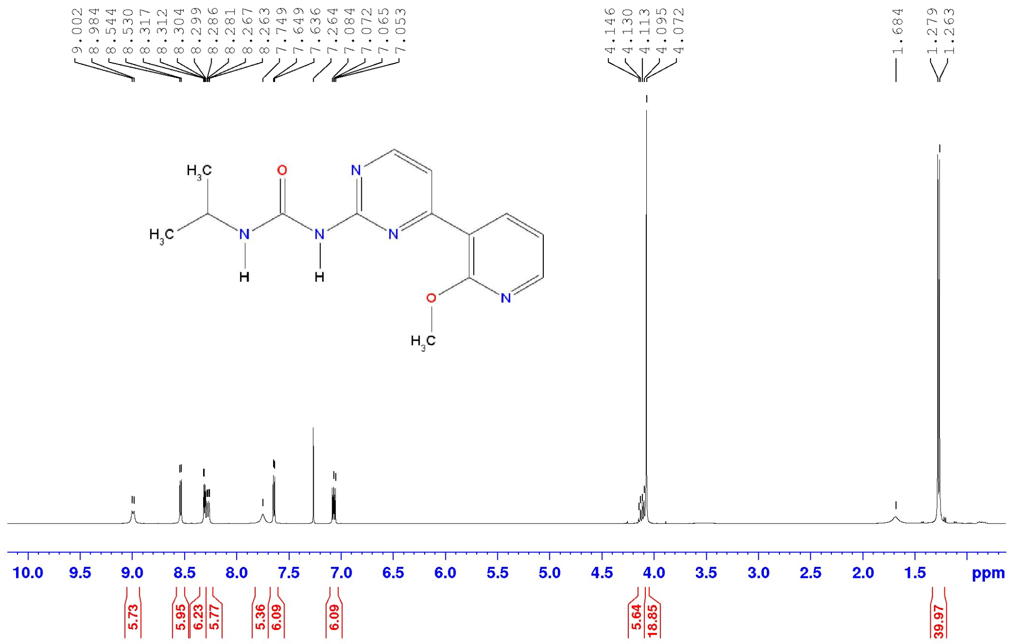

1-[4-(2-methoxypyridin-3-yl)pyrimidin-2-yl]-3-propan-2-ylurea

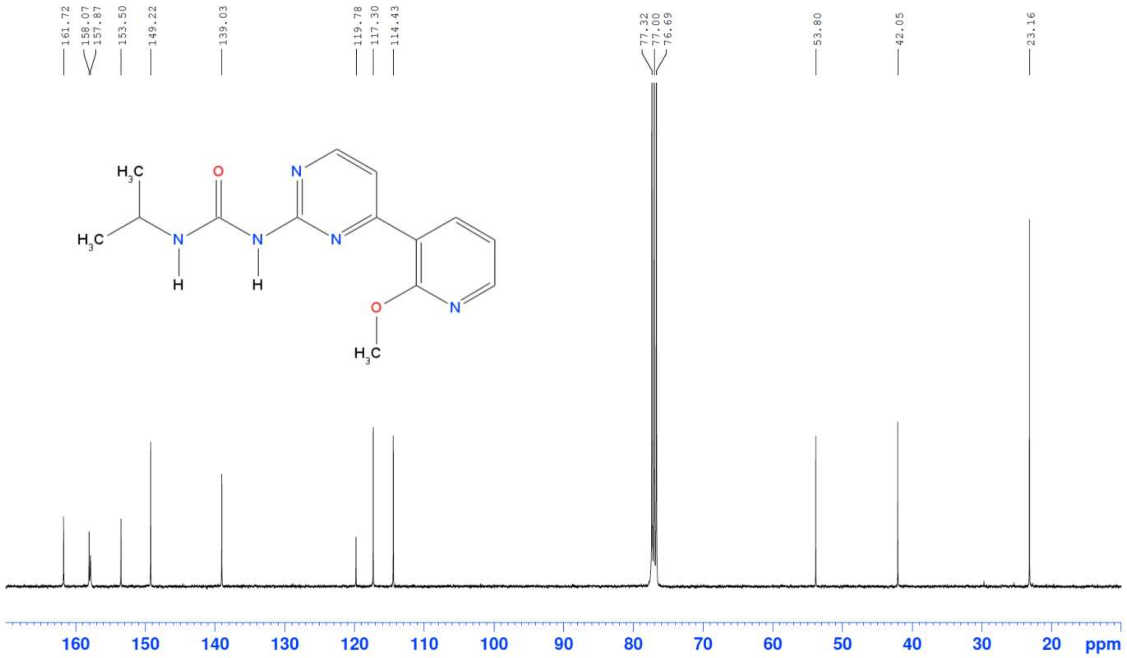

1-[4-(2-oxo-1,2-dihydropyridin-3-yl)pyrimidin-2-yl]-3-propan-2-ylurea

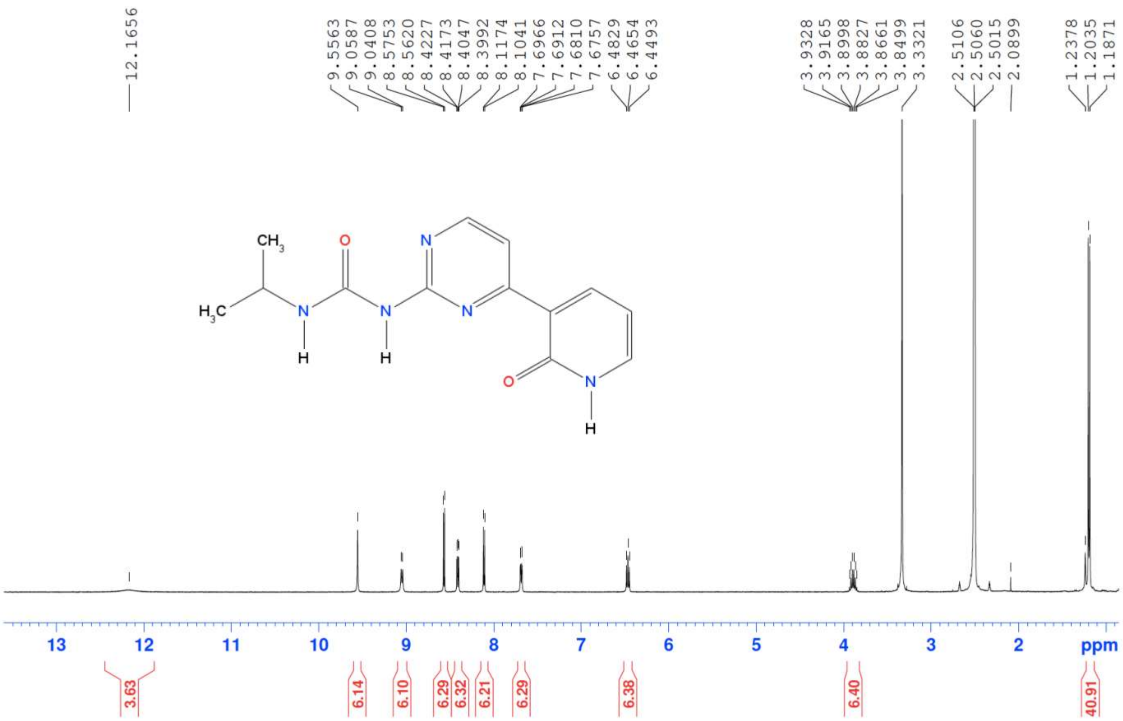

1-[4-(2-oxo-1,2-dihydropyridin-3-yl)pyrimidin-2-yl]-3-propan-2-ylurea

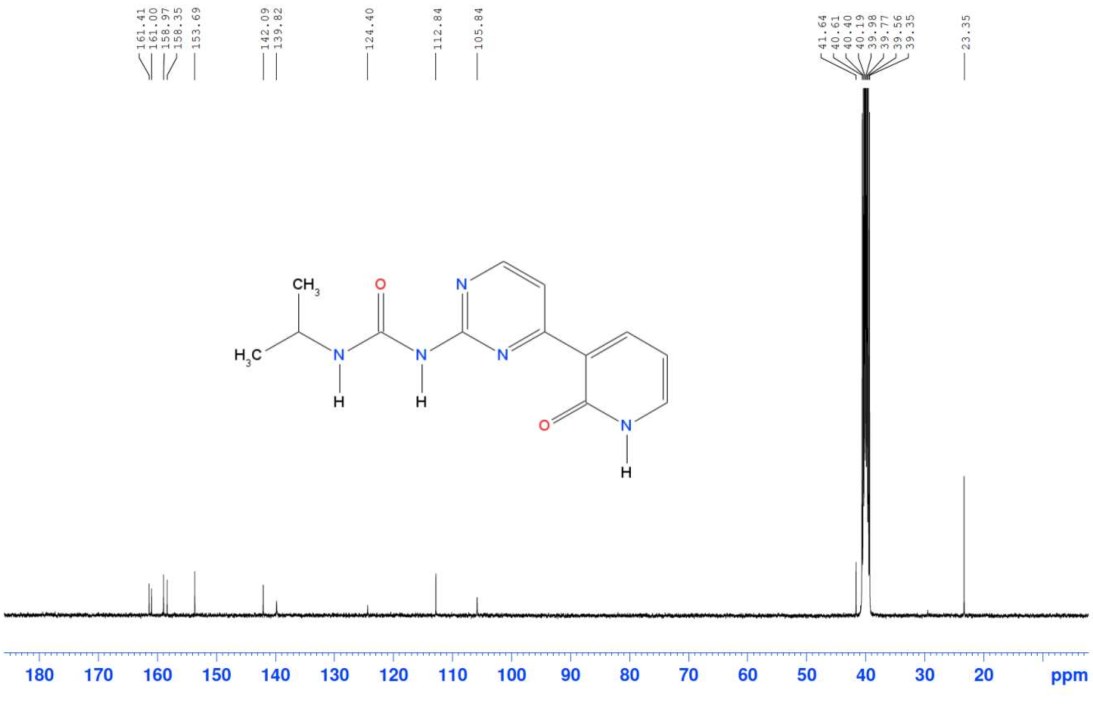

References

1. Huang, J.; Häussinger, D.; Gellrich, U.; Seiche, W.; Breit, B.; Meuwly, M. Hydrogen-

Bond and Solvent Dynamics in Transition Metal Complexes: A Combined Simulation and NMR-Investigation. *J. Phys. Chem. B* **2012**, *116*, 14406–14415.
2. Gellrich, U.; Huang, J.; Seiche, W.; Keller, M.; Meuwly, M.; Breit, B. Ligand Self-Assembling through Complementary Hydrogen-Bonding in the Coordination Sphere of a Transition Metal Center: The 6-Diphenylphosphanylpyridin-2(1H)-one System. *J. Am. Chem. Soc.* **2011**, *133*, 964–975.
3. Kolehmainen, E.; Ośmialowski, B.; Krygowski, T.M.; Kauppinen, R.; Nissinen, M.; Gawinecki, R. Substituent and temperature controlled tautomerism: Multinuclear magnetic resonance, X-ray, and theoretical studies on 2-phenacylquinolines. *J. Chem. Soc. Perkin Trans. 2* **2000**, 1259–1266.
4. Bader, R.F.W. *Atoms in Molecules: A Quantum Theory*; Oxford University Press: New York, 1990;
5. Espinosa, E.; Souhassou, M.; Lachekar, H.; Lecomte, C. Topological analysis of the electron density in hydrogen bonds. *Acta Cryst. B* **1999**, *55*, 563–572.
6. Espinosa, E.; Molins, E.; Lecomte, C. Hydrogen bond strengths revealed by topological analyses of experimentally observed electron densities. *Chem. Phys. Lett.* **1998**, *285*, 170–173.
7. Etter, M.C. Encoding and decoding hydrogen-bond patterns of organic compounds. *Acc. Chem. Res.* **1990**, *23*, 120–126.
8. Arunan, E.; Desiraju, G.R.; Klein, R.A.; Sadlej, J.; Scheiner, S.; Alkorta, I.; Clary, D.C.; Crabtree, R.H.; Dannenberg, J.J.; Hobza, P.; et al. Definition of the hydrogen bond (IUPAC Recommendations 2011). *Pure Appl. Chem.* **2011**, *83*, 1637–1641.
